# Salinity-Induced Palmella Formation Mechanism in Halotolerant Algae *Dunaliella salina* Revealed by Quantitative Proteomics and Phosphoproteomics

**DOI:** 10.3389/fpls.2017.00810

**Published:** 2017-05-23

**Authors:** Sijia Wei, Yangyang Bian, Qi Zhao, Sixue Chen, Jiawei Mao, Chunxia Song, Kai Cheng, Zhen Xiao, Chuanfang Zhang, Weimin Ma, Hanfa Zou, Mingliang Ye, Shaojun Dai

**Affiliations:** ^1^Key Laboratory of Saline-Alkali Vegetation Ecology Restoration in Oil Field, Alkali Soil Natural Environmental Science Center, Ministry of Education, Northeast Forestry UniversityHarbin, China; ^2^Key Laboratory of Separation Sciences for Analytical Chemistry, National Chromatographic R&A Center, Dalian Institute of Chemical Physics, Chinese Academy of SciencesDalian, China; ^3^College of Life and Environmental Sciences, Shanghai Normal UniversityShanghai, China; ^4^Department of Biology, Genetics Institute, Plant Molecular and Cellular Biology Program, Interdisciplinary Center for Biotechnology Research, University of FloridaGainesville, FL, Unites States

**Keywords:** palmella formation, *Dunaliella salina*, salinity stress, quantitative proteomics, phosphoproteomics

## Abstract

Palmella stage is critical for some unicellular algae to survive in extreme environments. The halotolerant algae *Dunaliella salina* is a good single-cell model for studying plant adaptation to high salinity. To investigate the molecular adaptation mechanism in salinity shock-induced palmella formation, we performed a comprehensive physiological, proteomics and phosphoproteomics study upon palmella formation of *D. salina* using dimethyl labeling and Ti^4+^-immobilized metal ion affinity chromatography (IMAC) proteomic approaches. We found that 151 salinity-responsive proteins and 35 salinity-responsive phosphoproteins were involved in multiple signaling and metabolic pathways upon palmella formation. Taken together with photosynthetic parameters and enzyme activity analyses, the patterns of protein accumulation and phosphorylation level exhibited the mechanisms upon palmella formation, including dynamics of cytoskeleton and cell membrane curvature, accumulation and transport of exopolysaccharides, photosynthesis and energy supplying (i.e., photosystem II stability and activity, cyclic electron transport, and C4 pathway), nuclear/chloroplastic gene expression regulation and protein processing, reactive oxygen species homeostasis, and salt signaling transduction. The salinity-responsive protein–protein interaction (PPI) networks implied that signaling and protein synthesis and fate are crucial for modulation of these processes. Importantly, the 3D structure of phosphoprotein clearly indicated that the phosphorylation sites of eight proteins were localized in the region of function domain.

## Introduction

The unicellular algae can develop a vegetative palmella in their life cycle, when exposed to various extreme environment conditions, such as salinity (Takouridis et al., 2015), copper (Sztrum et al., 2012), organic acids (Iwasa and Murakami, 1968), herbicide (Franqueira et al., 2000), oxidative stress (Wang et al., 2004a), and predators (Lurling and Beekman, 2006). The palmella formation in *Chlamydomonas reinhardtii* (Iwasa and Murakami, 1968; Lurling and Beekman, 2006; Sztrum et al., 2012) and *Pediastrum tetras* (Ellis, 1972) have been found to be regulated by free calcium concentration, starch accumulation, carotenoid/chlorophyll ratio, as well as the activities of catalase (CAT) and ascorbate peroxidase (APX). Similarly, for green motile flagellates of *Haematococcus pluvialis*, oxidative stress could induce its green motile cells transforming into enlarged red resting cysts (aplanospores) (Boussiba et al., 1999; Wang et al., 2004a; Han et al., 2012). In *H. pluvialis*, proteomics study revealed that the abundances of several proteins were induced, which were involved in reactive oxygen species (ROS) scavenging [e.g., superoxide dismutase (SOD), CAT and peroxidase (POD)], photosynthesis [e.g., ribulose-1,5-bisphosphate carboxylase/oxygenase large subunit (Rubisco LSU), phosphoglycerate kinase (PGK)], nitrogen assimilation [e.g., glutamine synthetase (GS)], and mitochondrial respiratory (e.g., mitochondrial ATPase β subunit) (Wang et al., 2004a). Moreover, the reduction of chlorophyll content, moderate declines in the maximal photosynthetic rate and the maximum quantum yield of photosystem (PS) II, as well as the significant increase in PS I activity were detected during the transformation of green vegetative cells to red aplanospores in *H. pluvialis* (Han et al., 2012). These indicates that the early stress response involves multiple enzymatic defense processes, which plays a critical role during the transition of green vegetative cells to red cysts (Wang et al., 2004a).

The halotolerant unicellular green algae *Dunaliella salina* is a model photosynthetic organism for studying plant adaptation to high salinity (Katz et al., 2007), which can adapt to a wide range of salinities ranging from about 0.05 M NaCl to saturation (around 5.5 M NaCl) (Liska et al., 2004). During their life cycle, *D. salina* may have a dominant palmella stage formed by a colony-like group of round non-motile cells to cope with extreme conditions (Borowitzka and Siva, 2007). When entering palmella stage, the cells usually lose their flagella and eyespot, become round, and excrete a slime exopolysaccharides (EPSs) layer outside the accumulation of green cells, but when returning to fresh medium at a “normal” salinity, algae cells usually reform their flagella and return to the motile and free-swimming state (Borowitzka and Siva, 2007).

It is reported that high salinity triggers palmella formation in *D. salina* (Montoya and Olivera, 1993), but the molecular mechanism of palmella formation is still unknown. Previous studies mainly focus on the mechanism of remarkable salinity and osmotic adaptability of *D. salina*. It is found that the *de novo* synthesis of compatible solute (i.e., glycerol and β-carotene) (Ben-Amotz and Avron, 1973), higher activity of Na^+^ extrusion systems (Pick, 2002), as well as active photosynthetic and energy metabolism (Liska et al., 2004; Katz et al., 2007; Alkayal et al., 2010) are crucial for salinity-response in *D. salina*. Importantly, proteomic research has provided new insights into the high salinity-responsive strategies in *D. salina*. The abundances of several proteins were salinity-regulated, which were involved in Calvin cycle, starch mobilization, energy production, protein synthesis/degradation, membrane structure stabilization, and signal transduction in *D. salina* (Liska et al., 2004; Katz et al., 2007). However, whether these fine-tuned molecular mechanism also happened in the process of salinity-induced palmella formation is poorly understood.

In this study, the salinity shock-induced palmella formation was analyzed in *D. salina*. By integrative analysis of morphology changes, photosynthesis, antioxidant enzyme activities, as well as protein abundance and phosphorylation level, we highlighted several important mechanisms upon palmella formation, such as photosynthetic modulation, ROS scavenging, gene expression regulation, and protein post-translational modification.

## Experimental procedures

### Cultivation, treatment, and biomass analysis

*D. salina* cells were cultivated in a modified medium containing 1 M NaCl under a 16/8 h light/dark cycle (light intensity 100 μmol photons × m^−2^ × s^−1^) at 26°C with shaking at 96 rpm (Katz and Avron, 1985). Cells in the stationary phase were transferred to fresh medium containing 1 M NaCl (control) and 3 M NaCl (salinity shock), respectively. After treatment, algae cells were used freshly or stored at −80°C for experiments.

The cell growth was evaluated by the absorbance of cultures at 630 nm in a spectrophotometer (Chen et al., 2009). The cell number was counted using a haemacytometer under light microscope. Three independent biological replicates for each sample were conducted for all the experiments. The morphology of *D. salina* cells were observed under Olympus BX53 Microscope (Olympus America Inc., Center Valley, PA, USA) equipped with Olympus DP72 digital camera system (Tokyo, Japan).

### Assessment of cell viability

The cell viability was estimated according to the method of Mendes et al. (2013). 3-(4,5-dimethylthiazol-2-yl)-2,5-diphenyltetrazolium bromide (MTT) stock solution (60 μl; 5 mg/ml) was applied to 250 μl cells for 60 min at 26°C with shaking at 96 rpm, and then the samples were centrifuged at 8,000 × g for 5 min. The formazan crystals were dissolved in 150 μl of dimethylsulfoxide and vortexed for 10 min. After centrifugation at 12,000 × g for 5 min, the supernatants were transferred to 96-well cell culture plate. The absorbance at 490 nm was measured using an iMark™ Microplate Reader (Bio-Rad, Richmond, CA, USA).

### Carbohydrate and glycerol content analysis

Carbohydrates were analyzed according to the method of Li et al. (2000). Ten milliliter culture was centrifuged at 12,000 × g for 10 min, and then 2 ml of supernatant was analyzed by the anthrone reagent method (Pistocchi et al., 1997). Glycerol content was determined according to the method of Chen et al. (2011).

### Chlorophyll content, photosynthesis oxygen evolution, respiration rate, chlorophyll fluorescence and P700 analysis

The chlorophyll contents were determined using a method described by Lichtenthaler and Wellburn (1983). Photosynthesis oxygen evolution and respiration rate were recorded by Clark-type oxygen electrode (Hansatech, UK) according to the method of Ma et al. (2005). The chlorophyll fluorescence parameters and P700 were detected using a pulse-amplitudemodulated chlorophyll fluorometer (Dual-PAM-100; Walz, Effeltrich, Germany) and an emitter-detector-cuvette assembly with a unit 101ED (ED-101US) according to the method of Ma et al. (2008).

### Enzyme activity assay

Antioxidant enzyme assays were performed essentially according to Suo et al. (2015). For the sample preparation of SOD, CAT, POD, APX, monodehydroascorbate reductase (MDHAR), dehydroascorbate reductase (DHAR), glutathione reductase (GR), glutathione S-transferase (GST), and glutathione peroxidase (GPX), 0.5 g of cells was homogenized on ice in 3 ml buffer [50 mM phosphate buffer (pH 7.8), 2% PVP-40, and 2 mM ascorbate (AsA)]. After centrifugation at 15,000 × g for 20 min at 4°C, the supernatants were used for enzyme activity assays according to the method of Zhao et al. (2016).

For glycerol-3-phosphate dehydrogenase (GPDH) activity assay, cells were ground to a fine powder in liquid nitrogen and resuspended in extraction buffer (100 mM Tris, 20 mM ascorbic acid, pH 6.9) (Chen et al., 2009). After being centrifuged at 34,000 × g for 20 min at 4°C, the supernatant was collected for GPDH activity assay. GPDH activity was determined using a plant GPDH assay kit according to manufacturer's instructions (IBL Hamburg, Germany).

Phosphoglucomutase (PGM) activity was determined using a PGM colorimetric assay kit according to manufacturer's instructions (BioVision, USA). For the H^+^-ATPase activity, 0.5 g of cells was used for the preparation of membranes. Plasmalemma vesicles were prepared as described by Kasamo (1988). The H^+^-ATPase activity was measured according to the method of Liang (1999).

### Generation rate of ${\text{O}}_{2}^{-}$ and contents of H_2_O_2_, AsA, DHA, GSSG, and GSH

The generation rate of ${\text{O}}_{2}^{-}$ was measured by monitoring the nitrite formation from hydroxylamine in the presence of ${\text{O}}_{2}^{-}$ and the absorbance in the aqueous solution was read at 530 nm (Zhao et al., 2016). The content of H_2_O_2_ was measured by monitoring the A_410_ of titanium-peroxide complex according to the method of Suo et al. (2015).

For the contents of reduced AsA, oxidized AsA (DHA), oxidized glutathione (GSSG), and reduced glutathione (GSH), 0.5 g of cells was homogenized on ice in 3 ml 5% trichloroacetic acid. After centrifugation at 15,000 × g for 15 min at 4°C, the supernatants were used for substrate content assays. Total AsA and reduced AsA were determined by recording the absorbance changes at 525 nm (Kampfenkel et al., 1995). DHA content was estimated from the difference between assays with and without dithiothreitol (DTT). The extracts were also used for GSH/GSSG assay according to the method of Baker et al. (1990). The concentration of GSH was calculated from the differences between total glutathione and GSSG assays (Haghjou et al., 2009).

### Protein sample preparation

The proteins for differential stable isotope labeling experiments were extracted in the modified extraction buffer (900 mM sucrose, 100 mM Tris-HCl (pH 8.0), 65 mM DTT, 1 mM MgCl_2_, 1% Triton X-100 (v/v), 1 mM phenylmethanesulfonyl fluoride, 1% protease inhibitor cocktail (v/v), 1 mM EDTA, 1 mM EDGA, 1 mM NaF, 1 mM Na_3_VO_4_, 1 mM Na_4_O_7_P_2_, and 1 mM C_3_H_7_Na_2_O_6_P) according to the phenol-methanol methods of Suo et al. (2015). Protein concentration was determined using a Quant-kit according to manufacture instructions (GE Healthcare, USA).

### Protein in-solution digestion, dimethyl labeling and phosphopeptide enrichment

The protein was resuspended in the denaturing buffer containing 8 M urea and 100 mM triethylammonium bicarbonate (pH 8.0) (Bian et al., 2012; Song et al., 2014), and then was reduced by DTT and alkylated by iodoacetamide. After that, the solution was diluted with 100 mM triethylammonium bicarbonate and trypsin was added with an enzyme-to-protein ratio of 1/25 (w/w) and incubated at 37°C overnight.

For the light and heavy dimethyl labeling, 500 μl of CH_2_O (4%, v/v) and CD_2_O (4%, v/v) was added to the tryptic digests from *D. salina* cells cultured in 1 M NaCl (control) or 3 M NaCl (salinity shock for 6 h) medium, respectively, then 500 μl of freshly prepared NaBH_3_CN (0.6 M) was subsequently added. The detailed labeling and desalted procedure were the same as described in the method of Song et al. (2014).

The phosphopeptides from 2.0 mg of labeled protein digestion mixture were enriched by Ti^4+^-immobilized metal ion affinity chromatography (IMAC) microspheres (Zhou et al., 2008, 2013) and then were resuspended in 30 μl 5% formic acid.

### LC-MS/MS analysis

For the reversed-phase (RP) liquid chromatography (LC)-MS/MS analysis, a 12 cm × 75 μm i.d. capillary column packed with C18 AQ particles (3 μm, 120 Å) was used as the separation column, and 0.1% formic acid in water and in acetonitrile was used as mobile phases A and B, respectively (Song et al., 2012).

For the quantitative proteomic analysis, 20 μg tryptic digests were loaded onto the strong cation exchange (SCX) monolithic column and then were eluted by 11 salt stepwise elutions to perform the 2-D RPLC-MS/MS analysis (Wang et al., 2007). Each RPLC-MS/MS elution step was performed in a 72 min gradient from 5 to 35% acetonitrile. For the two-dimensional RPLC-MS/MS phosphoproteomic analysis, a RP-strong cation exchange chromatography (SCX) biphasic column combined with C18 analytical column was used (Bian et al., 2012, 2016). Briefly, the phosphopeptides were manually loaded onto the RP-SCX column and then eluted from the RP segment to SCX segment by a 160 min RP gradient LC-MS/MS (0 mM). Then, a series of stepwise elutions with salt concentrations of 24, 40, 56, 72, 100, 200, and 1,000 mM NH_4_AC were used to elute phosphopeptides from the SCX column to the second dimensional C18 separation column. Finally, the RPLC-MS/MS separation was performed with a 117 min gradient from 5 to 25% acetonitrile.

The LTQ-Orbitrap Velos mass spectrometer (Thermo Fisher Scientific) was operated in data-dependent MS/MS acquisition mode. Full mass spectra were acquired in the Orbitrap at a resolution of 60,000 (*m/z* 400). The 20 most intense precursors were selected for fragmentation via collision induced dissociation in the LTQ. For the phosphopeptide analysis, the multistage activation was enabled. The dynamic exclusion function was set as follows: repeat count 2, repeat duration 30 s, and an exclusion duration of 60 s.

### Protein identification and relative quantification

Protein identification and quantification are based on the MaxQuant software (version 1.1.1.36) according to the standard workflow (Cox and Mann, 2008). Because the m/z of the light and heavy labeled peptides were different at MS1 level, this mass difference can be distinguished by the mass spectrometer with resolution (R > ~10,000). Quantification is achieved by comparing the intensities of the labeled peptides at MS1 level (Boersema et al., 2009). At least 1.5 fold change is consider as differential proteins in response to salinity.

The database search was performed on the Andromeda search engine against the Chlorophyta protein database (from NCBI, containing 156,988 entries) concatenated with reversed sequences for evaluating of FDR (Cox et al., 2011). Carbamidomethylation on cysteine was set as a fixed modification, whereas oxidation on methionine was set as a variable modification. For phosphopeptides, Phospho (STY) was also set as variable modifications. FDR were set to 1% at phosphorylation site, peptide, and protein group level. For protein quantitation, only the proteins with a BenjaminiHochberg corrected *p*-value < 0.05 based on significance B were reported by the MaxQuant software (Cox and Mann, 2008). A more detailed parameters setting for database search was illustrated in our previous studies (Song et al., 2012). The mass spectrometry proteomics data have been deposited to the ProteomeXchange Consortium via the PRIDE (Vizcaino et al., 2014) partner repository with the dataset identifier PXD005443 and PXD005501.

### Phosphorylation site localization

Only the phosphorylation sites with a reported localization probability of at least 0.75 were used for further analysis (Sharma et al., 2014). For quantification analysis, we used the intensity-weighted ratio significance values reported by MaxQuant to determine significantly changed phosphorylation sites.

### Protein classification, subcellular location and protein–protein interaction prediction

Protein functional domains were predicted using the position-specific iterated BLAST and pattern-hit initiated BLAST programs (http://www.ncbi.nlm.nih.gov/BLAST/). Combined BLAST alignments with Gene Ontology and knowledge from literatures, proteins were classified into different categories.

The subcellular locations of proteins were determined using five internet tools: Yloc (http://abi.inf.uni-tuebingen.de/Services/YLoc/webloc.cgi), LocTree3 (https://rostlab.org/services/loctree3/), Plant-mPLoc (http://www.csbio.sjtu.edu.cn/bioinf/plant-multi/.), ngLOC (http://genome.unmc.edu/ngLOC/index.html), and TargetP (http://www.cbs.dtu.dk/services/TargetP/). The confident results have the consistent predictions from at least two tools. For the inconsistent prediction results among five tools, subcellular localizations for corresponding proteins were predicted based on literatures.

The protein–protein interactions (PPI) were predicted using the web-tool STRING 10 (http://string-db.org). The proteins homologs in *Arabidopsis* were analyzed by sequence BLASTing in TAIR database (http://www.arabidopsis.org/Blast/index.jsp), and then the homologs were subjected to the molecular interaction tool of STRING 10 for creating the proteome-scale interaction network (Suo et al., 2015).

### Protein 3D structure analysis and multiple sequence alignment

The protein three-dimensional (3D) structure homology model was pre-calculated in SWISS-MODEL workspace (https://swissmodel.expasy.org) (Arnold et al., 2006). The 3D structures and the phosphorylation site were displayed using the SPDBV (version 4.1) software (Arnold et al., 2006). Multiple sequence alignment was performed with sequences from multiple species using Clustal Omega software (http://www.ebi.ac.uk/Tools/msa/clustalo/).

### Statistical analysis

All physiological results were presented as means ± standard deviation of at least three replicates. Salinity-responsive proteins (SRPs) were quantified in at least two of the three replicates. The data were analyzed by Student's *t*-test using the statistical software SPSS 18.0 (SPSS Inc., Chicago, IL). A *p*-value less than 0.05 was considered statistically significant.

## Results

### Cell growth and viability upon salinity-induced palmella formation

**Figure 1:**
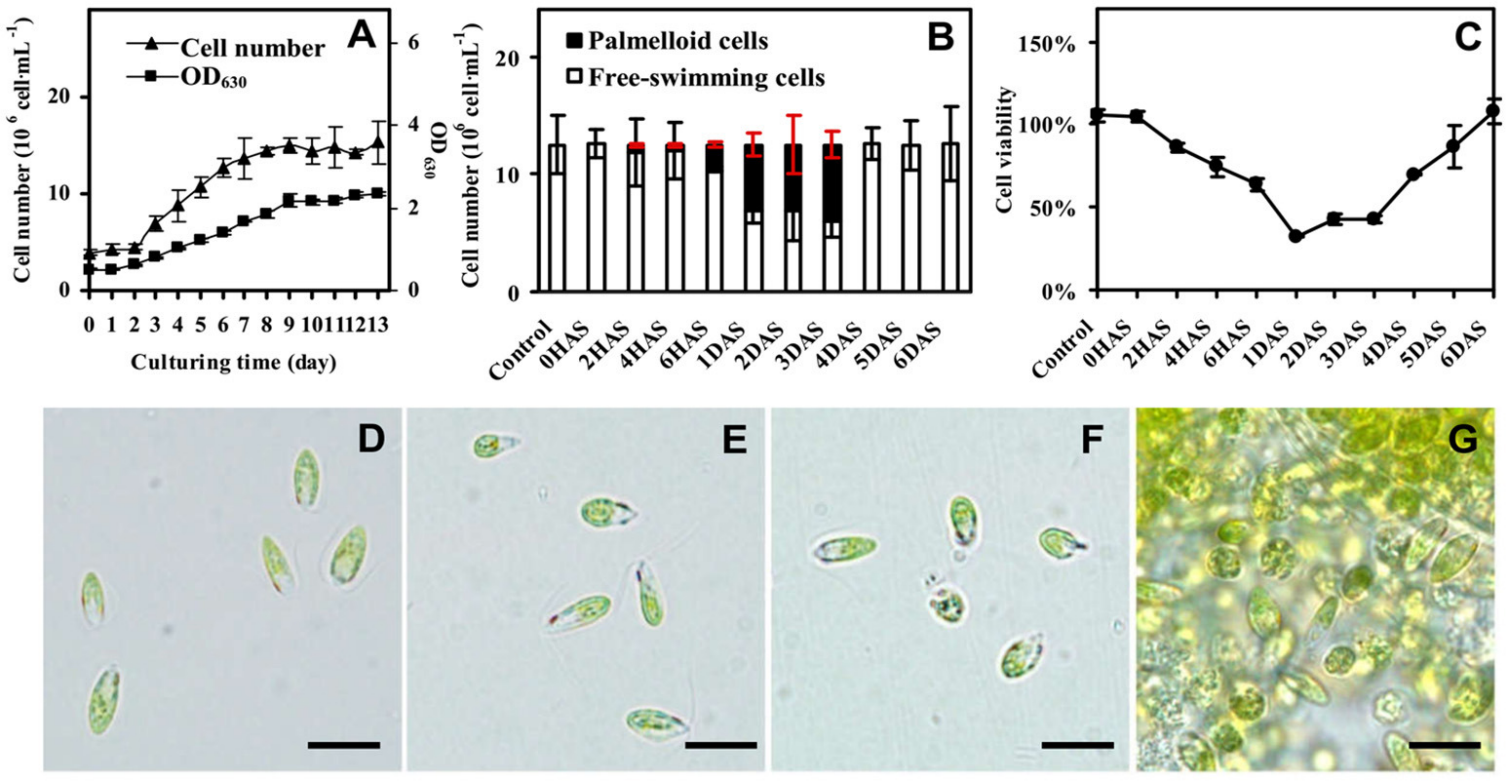
**Growth and salinity-induced palmella formation of ***Dunaliella salina***. (A)** Cell growth curves of *D. salina* grown in medium containing 1 M NaCl. **(B)** The number of palmella cells and free-swimming cells in medium containing 3 M NaCl from 0 h after salinity shock (HAS) to 6 days after salinity shock (DAS). **(C)** Cell viability in 3 M NaCl medium. **(D)** Morphology of cells in 1 M NaCl medium. **(E)** Morphology of cells at 0 HAS. **(F)** Morphology of free-swimming cells at 2 HAS. **(G)** Morphology of palmella cells at 6 HAS. The group of accumulated palmella cells were surrounded by exopolysaccharides. Values of cell number and viability are means ± standard deviation (*n* = 3), bar = 10 μm.

The *D. salina* cells were cultured under medium containing 1 M NaCl (Katz and Avron, 1985). The cell number was increased gradually, and cell growth entered stationary phase after 10 days of culturing (Figure 1A), and then the cells were directly transferred to medium containing 3 M NaCl (Figure 1B and Supplemental Figure S1). The number of palmella cells was about 0.63 ± 0.16 × 10^6^ per milliliter (about 5% of the total cell number) at 2 h after salinity shock (HAS), and reached to 2.08 ± 0.25 × 10^6^ per milliliter (17%) at 6 HAS. The maximum of palmella cell number was detected as 6.09 ± 0.38 × 10^6^ per milliliter (49%) at 1 days after salinity shock (DAS), indicating the *D. salina* cells formed a palmella stage under the salinity induction at 1 DAS. The palmella stage continued for 3 days and reverted to free-swimming cell stage at 4 DAS (Figure 1B). The change of free-swimming cell number was also reflected from the medium color during this course (Supplemental Figure S1). After salinity shock, the medium color turned from green at 0 HAS to light green gradually at 1 DAS, and then reverted to dark green at 6 DAS (Supplemental Figure S1).

Besides, the change of cell viability evaluated by MTT assay was consistent with the number of free-swimming cells (Figure 1C). The cellular viability was gradually reduced at 6 HAS, reached to the lowest value at 1 DAS, and gradually increased from 4 DAS. In addition, the cell morphology has obvious changes during salinity shock-induced palmella formation. The free-swimming cells under 1 M NaCl were green, biflagellate, and rod to ovoid shaped, as well the cell length was about 12 μm (Figure 1D). The cell morphology didn't show obvious changes at 0 HAS (Figure 1E), but some cells started to lose their flagella and changed to round and immotile at 2 HAS (Figure 1F). More than 17% of free-swimming cells formed immotile palmella at 6 HAS, and the accumulated palmella cells lost their flagella and eyespot, appearing round (Figure 1G). All these indicate that 3 M NaCl shock induces the formation of a short palmella stage, but the palmella cells reverted to free-swimming cells at 4 DAS. During this course, there were three morphotypes at 6 HAS, which were predominantly motile cells, motile form with a prominent palmelloid cells (non-motile, mucilage rich), and palmelloid form with a weakly motile cells (Buchheim et al., 2010). Thus, 6 HAS is a critical time point for the transformation from free-swimming cells to palmella cells.

### Osmotic regulation upon salinity-induced palmella formation

**Figure 2:**
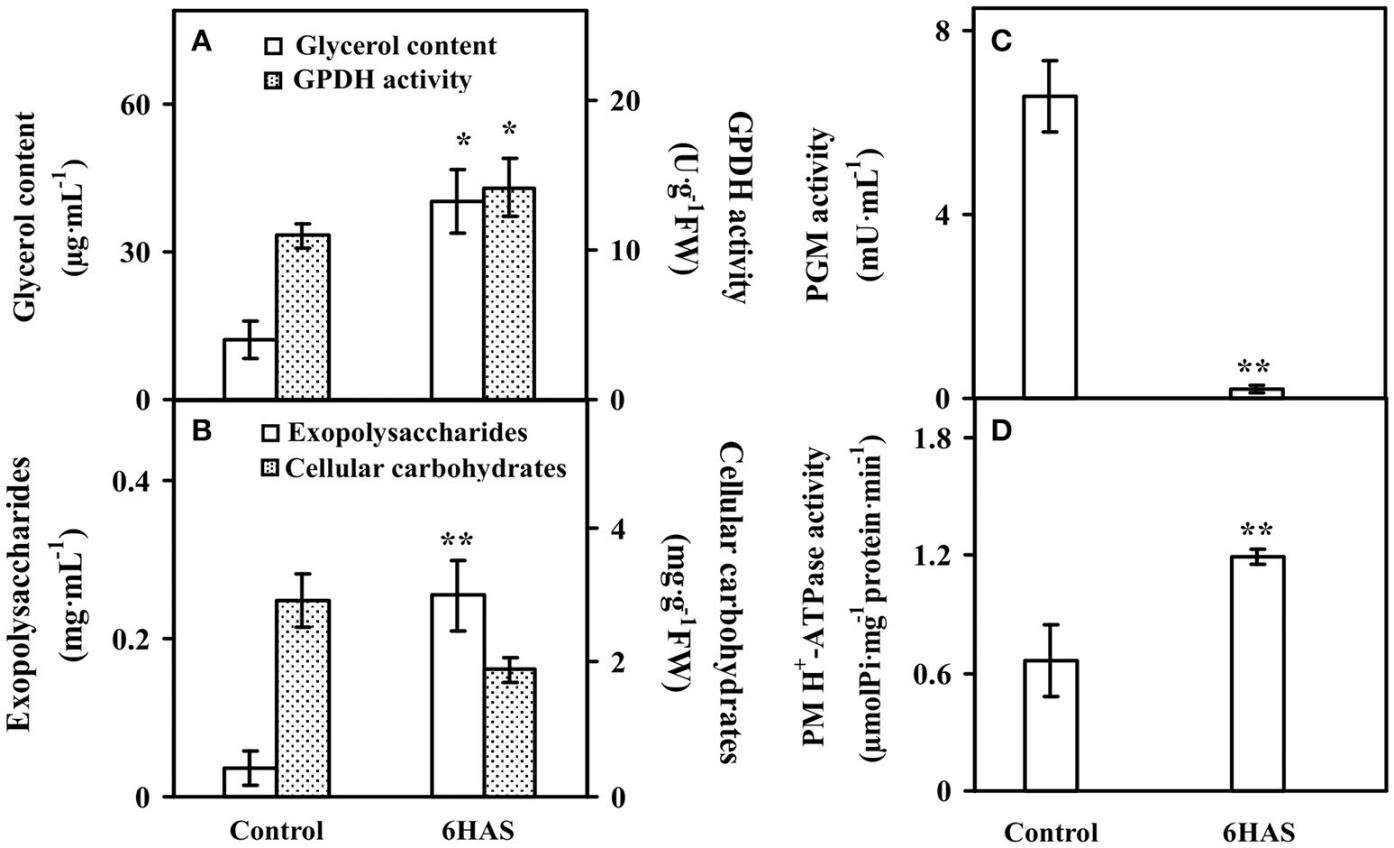
**The contents of (A)** glycerol, **(B)** exopolysaccharides (EPSs) and cellular carbohydrates, and activities of **(A)** glycerol-3-phosphate dehydrogenase (GPDH), **(C)** phosphoglucomutase (PGM), and **(D)** plasma membrane (PM) H^+^-ATPase upon palmella formation of *Dunaliella salina*. Values are means ± standard deviation based on three independent determinations for control and cells for 6 h after salinity shock (HAS), and bars indicate standard deviations. ^*^ and ^**^ indicate values that differ significantly from controls at *p* < 0.05 and *p* < 0.01, respectively, according to Student's *t*-test.

The glycerol content was significantly increased, and the activity of GPDH for glycerol synthesis was also obviously increased at 6 HAS (Figure 2A). Besides, the content of EPSs was significantly induced, but the cellular carbohydrate content was slightly decreased in cells at 6 HAS (Figure 2B). In addition, the activity of a carbohydrate metabolic enzyme PGM, which catalyzes the bidirectional interconversion of glucose-1-phosphate and glucose-6-phosphate, was significantly reduced at 6 HAS (Figure 2C). Importantly, the activity of plasma membrane (PM) H^+^-ATPase was significantly induced at 6 HAS, indicating a proton-motive force generator for sucrose transporter was enhanced (Figure 2D).

### Chlorophyll content and fluorescence parameters

**Figure 3:**
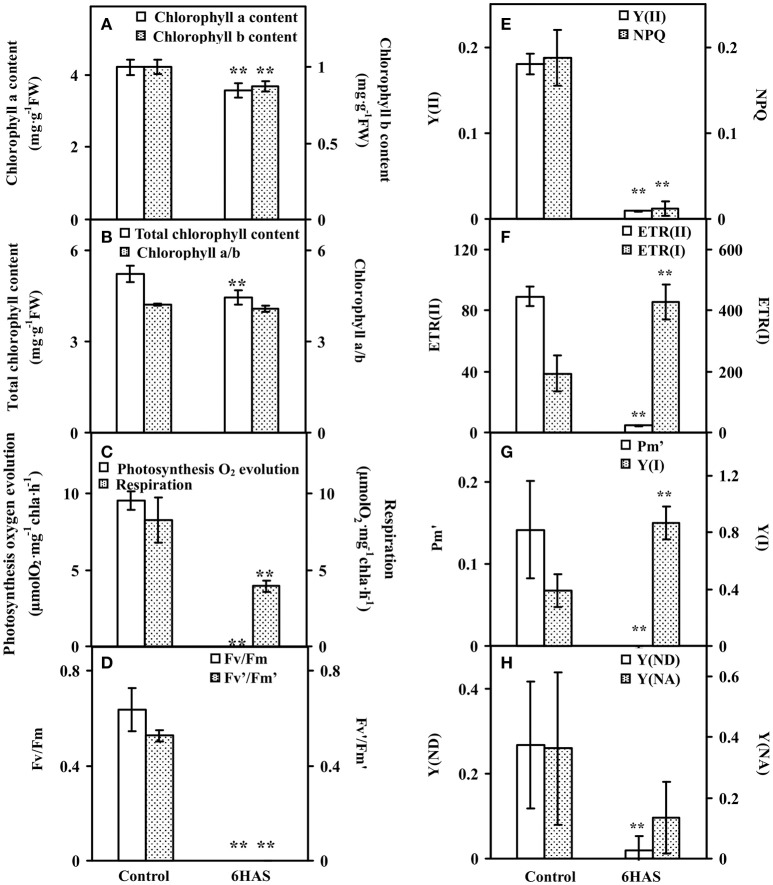
**Chlorophyll contents and photosynthetic parameters upon palmella formation of ***Dunaliella salina***. (A)** The contents of chlorophyll *a* and chlorophyll *b*. **(B)** Total chlorophyll content and chlorophyll *a*/*b*. **(C)** Photosynthesis oxygen evolution and respiration rate. **(D)** PSII maximum quantum yield (Fv/Fm) and the excitation capture efficiency of open centers (Fv'/Fm'). **(E)** Effective PSII quantum yield (Y(II)) and coefficient of non-photochemical quenching (NPQ). **(F)** Electron transport rates of PSII (ETR(II)) and PSI (ETR(I)). **(G)** Maximum P700 change (Pm') and photochemical quantum yield of PSI (Y(I)). **(H)** Non-photochemical quantum yields of PSI caused by donor-side limitation (Y(ND)) and by acceptor-side limitation (Y(NA)). The values are presented as means ± standard deviation (*n* = 3). ^**^ indicates significant differences among control and 6 h after salinity shock (HAS) is *p* < 0.01.

The contents of chlorophylls in control cells and salinity-shocked cells at 6 HAS were detected (Figure 3). The contents of chlorophyll *a* (Figure 3A), chlorophyll *b* (Figure 3A), and total chlorophyll (Figure 3B) were all declined, but the chlorophyll *a*/*b* (Figure 3B) didn't show obvious changes at 6 HAS.

Photosynthetic oxygen evolution and respiration rates were all decreased in the salinity- shocked cells when compared with those in the control cells (Figure 3C). In addition, chlorophyll fluorescence parameters were monitored to evaluate the photosynthetic performance. The PSII related parameters, including PSII maximum quantum yield (Fv/Fm) (Figure 3D), the excitation capture efficiency of open centers (Fv'/Fm') (Figure 3D), the effective PSII quantum yield (Y(II)) (Figure 3E), non-photochemical quenching (NPQ) (Figure 3E), and electron transport rate (ETR(II)) (Figure 3F), were declined in salinity-shocked cells. Furthermore, PSI related parameters, such as maximum fluorescence yield (Pm') (Figure 3G), non-photochemical quantum yields of PSI caused by donor-side limitation (Y(ND)) (Figure 3H) and by acceptor-side limitation (Y(NA)) (Figure 3H), were declined, whereas photochemical quantum yield of PSI (Y(I)) (Figure 3G) and electron transport rate (ETR(I)) were increased in salinity-shocked cells (Figure 3F).

### Antioxidant enzyme activities and metabolite contents upon salinity shock-induced palmella formation

**Figure 4:**
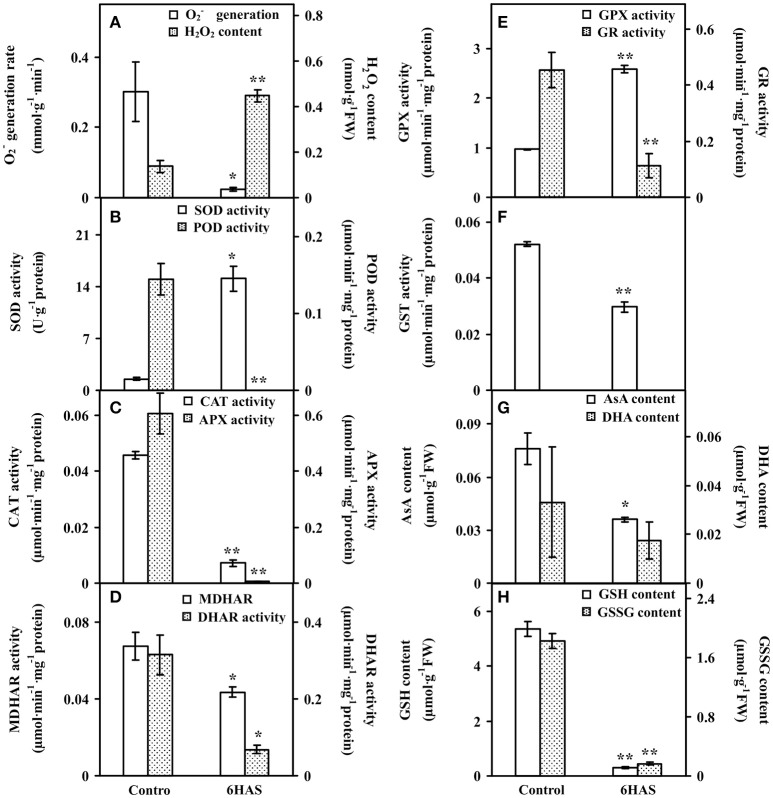
**The ROS production, antioxidant enzyme activities, and contents of metabolites in ***Dunaliella salina***. (A)** ${\text{O}}_{2}^{-}$ generation rate and H_2_O_2_ content. **(B)** Activities of superoxide dismutase (SOD) and peroxidase (POD). **(C)** Activities of catalase (CAT) and ascorbate peroxidase (APX). **(D)** Activities of monodehydroascorbate reductase (MDHAR) and dehydroascorbate reductase (DHAR). **(E)** Activities of glutathione peroxidase (GPX) and glutathione reductase (GR). **(F)** Glutathione S-transferase (GST) activity. **(G)** Contents of reduced ascorbate (AsA) and oxidized ascorbate (DHA). **(H)** Contents of reduced glutathione (GSH) and oxidized glutathione (GSSG). The values are presented as means ± standard deviation (*n* = 3). ^*^ and ^**^ indicate significant differences among control and 6 h after salinity-shock (HAS) are *p* < 0.05 and *p* < 0.01, respectively.

To evaluate the ROS level and the dynamics of ROS scavenging system in cells during palmella formation, the ${\text{O}}_{2}^{-}$ generation rate, H_2_O_2_ content, the activities of nine antioxidant enzymes, as well as the contents of metabolites in ROS scavenging system were analyzed (Figure 4). The ${\text{O}}_{2}^{-}$ generation rate was declined whereas the H_2_O_2_ content and GPX activity were increased at 6 HAS (Figures 4A,E). The activity of SOD was initially increased to catalyze the dismutation of ${\text{O}}_{2}^{-}$ into oxygen and H_2_O_2_ at 6 HAS (Figure 4B). However, the activities of POD and CAT in charge of the conversion of H_2_O_2_ to H_2_O were decreased at 6 HAS (Figures 4B,C). Moreover, the activities of enzymes (i.e., APX, MDHAR, DHAR, and GR) as well as the contents of AsA and DHA in GSH-AsA cycle were also decreased at 6 HAS (Figures 4C–E,G), so did the contents of GSSG and GSH (Figure 4H) and the activity of GST (Figure 4F).

### Proteomic analysis upon salinity shock-induced palmella formation

**Figure 5:**
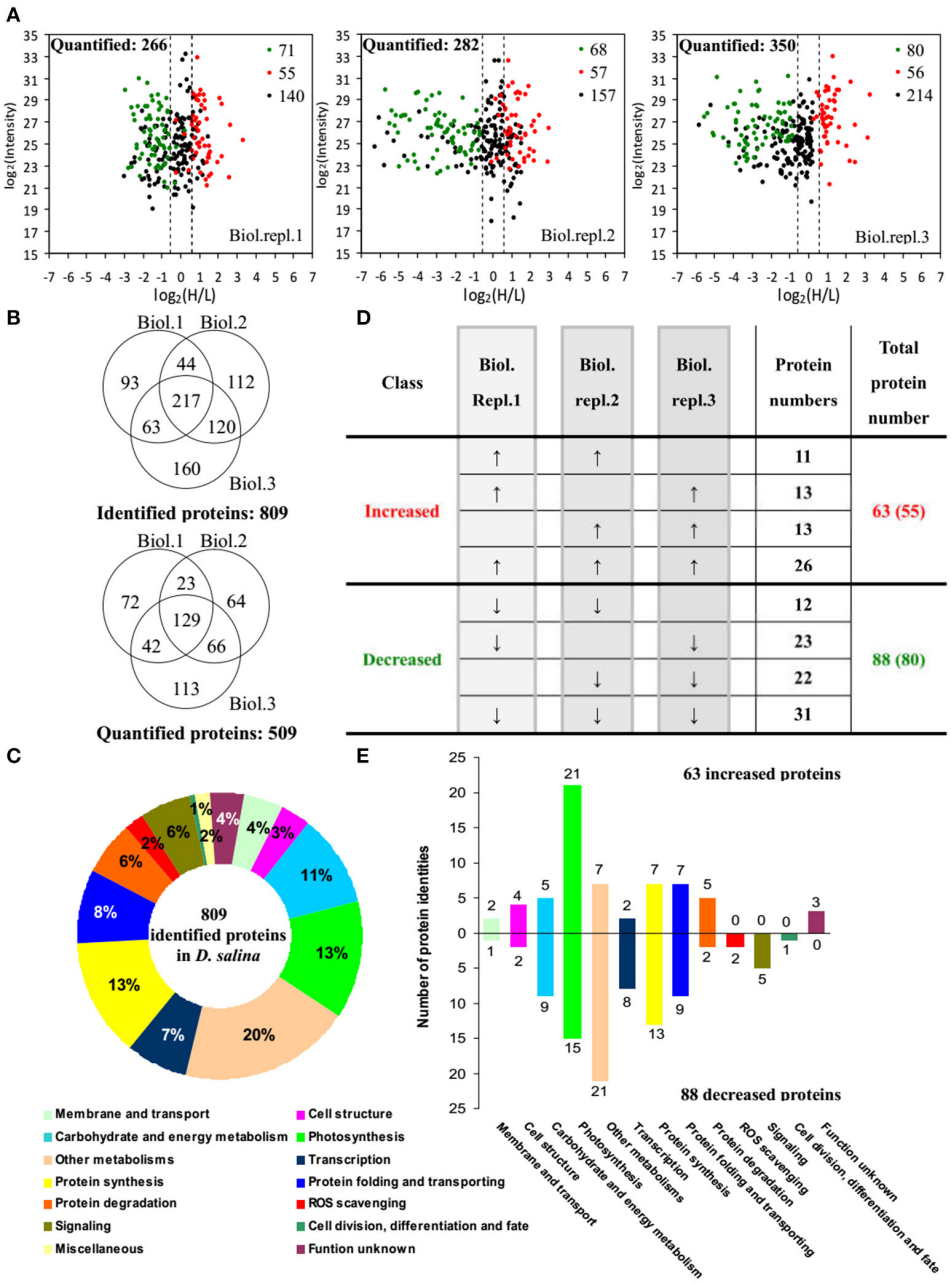
**Comparison of proteome in free-swimming cells and cells upon palmella formation of ***Dunaliella salina***. (A)** The diagrams showing for each quantified protein the change in abundance between heavy (H; salinity shock) and light (L; control) label (H/L; x axis) as a function of the signal intensity (y axis) recorded in the mass spectrometer. The dashed lines mark the border for a decrease or increase in protein by more than a factor of 1.2 [log _2_(H/L) < −0.6 or >0.6]. Most proteins (small black points) did not change in abundance between the two culture conditions. Significantly regulated proteins are depicted as red/green points, and red/green points indicate significantly increased/decreased proteins. **(B)** Overlap among the three biological replicate experiments for all proteins. 809 proteins were identified and 509 proteins were quantified. Three biological replicate experiments for each sample were performed by LTQ-Orbitrap Velos MS/MS analysis. The obtained results were given in Supplemental Table S1. **(C)** Functional category of 809 identified proteins in *D. salina*. **(D)** Classifications of quantitative proteins significantly change in three biological experiments. A down arrow (↓) indicates significant decrease and an up arrow (↑) significant increase upon palmelloid formation. The protein changes with at least two biological replicates is considered as significant changed protein. There are 63 increased protein species (representing 55 unique proteins) and 88 decreased protein species (80 unique proteins). **(E)** Functional category of salinity increased and decreased proteins. The columns above and under the x-axis represent the amounts of increased and decreased proteins, respectively. The number of increased/decreased proteins are marked on the column.

By using differential stable isotope labeling coupled with mass spectrometry approaches, 809 proteins were identified in *D. salina* cells (Figures 5A,B, Supplemental Table S1, and Supplemental Figure S2). This composes the largest protein database of *D. salina* so far (Supplemental Table S1). Among them, 509 proteins were quantified in three biological replicates (Figures 5A,B). On the basis of the Gene Ontology, BLAST alignment, KEGG database and information from the literature, the 809 proteins were classified into 14 functional categories (Figure 5C and Supplemental Table S1). Among them, other metabolism (20%), protein synthesis (13%), photosynthesis (13%), carbohydrate and energy metabolism (11%) were over-represented (Figure 5C).

Among these proteins, 151 SRPs were quantified in at least two of the three replicates, including 63 salinity-increased proteins, and 88 salinity-decreased proteins (Figure 5D, Tables 1, 2, and Supplemental Table S2). They were classified into 13 functional categories: membrane and transport, cell structure, carbohydrate and energy metabolism, other metabolisms, photosynthesis, transcription, protein synthesis, protein folding and transporting, protein degradation, ROS scavenging, signaling, cell division, differentiation and fate, and function unknown (Figure 5E and Supplemental Table S2). Among them, photosynthesis (24%), other metabolism (19%) and protein synthesis (13%) were predominant (Figure 5E). Most proteins involved in other metabolism, ROS scavenging, transcription, protein synthesis, and signaling were salinity-decreased (Figure 5E and Tables 1, 2). Besides, we found three SRPs involved in membrane and transport, and six involved in cell structure were changed upon palmella formation (Tables 1, 2).

**Table 1:** **Salinity shock-increased proteins upon palmella formation of ***D. salina*****.

**Gi number^a^**	**Protein name^b^**	**Protein abbreviation**	**Sub. Loc.^c^**	**Protein function^d^**	**Ratio H/L^e^**
**MEMBRANE AND TRANSPORT (2)**					
1495363	H(+)-transporting ATP synthase	H-ATP	Mit	H^+^ transport	1.51 ± 0.59
144577511	Bin/Amphiphysin/Rvs domain-containing protein^*^	BAR	PM	Membrane curvature	3.47 ± 0.88
**CELL STRUCTURE (4)**					
134142243	Tubulin β chain	TUB^1^	Cyt	Cytoskeleton microtubule	6.92 ± 3.96
135453	Tubulin β chain	TUB^2^	Cyt	Cytoskeleton microtubule	6.65 ± 2.78
116000450	Tubulin β chain	TUB^3^	Cyt	Cytoskeleton microtubule	5.89 ± 2.81
1279362	Striated fiber-assemblin	SFA	Cyt	Flagellar assembly	4.29 ± 0.52
**CARBOHYDRATE AND ENERGY METABOLISM (5)**					
146552013	Mitochondrial NADH: ubiquinone oxidoreductase 19 kDa subunit	NQO	Mit	Respiratory chain	4.65 ± 1.30
300263055	Mitochondrial F1F0 ATP synthase	F1F0-ATP	Mit^#^	ATP synthesis	5.12 ± 0.53
119358798	ATP synthase F1 β subunit	F1-ATPB	Mit	ATP synthesis	1.78 ± 0.15
119350547	ATP synthase F1 β subunit (F1-ATPB)	F1-ATPB	Mit	ATP synthesis	2.29 ± 0.45
307103070	ATP synthase F1 β subunit^*^	F1-ATPB	Mit	ATP synthesis	1.86 ± 0.31
**PHOTOSYNTHESIS (21)**					
115828	PSII Light-harvesting chlorophyll a/b binding protein type I	LHCb^1^	Chl	Light harvesting	1.70 ± 0.90
62199619	PSII Chloroplast Tidi	LHCb^2^	Chl^#^	Light harvesting	2.18 ± 1.08
246880792	PSII protein VI	PsbVI	Chl^#^	PS II stability	5.18 ± 3.36
246880776	PSII protein H	PsbH	Chl	PS II stability	2.28 ± 0.42
11135341	Cytochrome b6-f complex iron-sulfur subunit	Cytb_6_f^1^	Chl	Electron transport	3.22 ± 0.09
246880725	Cytochrome f	Cytb_6_f^2^	Chl	Electron transport	1.89 ± 0.48
225580693	PSI Light-harvesting chlorophyll a/b protein 3	LHCa3	Chl	Light harvesting	1.97 ± 0.55
246880738	PSI subunit VII	PsaVII/PsaC	Chl	PS I stability	2.35 ± 0.26
158274090	PSI reaction center subunit II	PsaII/PsaD	Chl	PS I stability	2.98 ± 1.08
12831160	PSI PsaG-like protein	PsaG	Chl	PS I stability	2.09 ± 0.98
246880800	PSI P700 chlorophyll a apoprotein A1	PsaA	Chl	PS I stability	2.08 ± 0.41
246880764	PSI P700 chlorophyll a apoprotein A2	PsaB	Chl	PS I stability	2.20 ± 0.63
2224380	PSI assembly protein Ycf3	Ycf3	Chl^#^	PS I stability	2.38 ± 0.31
132167	RuBisCO activase	RCA	Chl	Carbon fixation	2.38 ± 0.22
307107355	Phosphoribulokinase precursor	PRK	Chl	Carbon fixation	1.68 ± 0.26
246880751	ATP synthase CF1 β subunit	CF1-ATPB	Chl^#^	ATP synthesis	2.02 ± 0.38
5748664	ATP synthase CF1 β subunit	CF1-ATPB	Chl^#^	ATP synthesis	1.74 ± 0.46
170293993	ATP synthase CF1 β subunit	CF1-ATPB	Chl^#^	ATP synthesis	3.18 ± 1.36
333691279	ATP synthase CF1 γ subunit	CF1-ATPC	Chl	ATP synthesis	2.01 ± 0.07
246880770	ATP synthase CF1 ε subunit	CF1-ATPE	Chl^#^	ATP synthesis	2.62 ± 0.35
109726687	Limiting CO_2_ inducible protein^*^	LCIP	Chl^#^	CO_2_ transport	1.64 ± 0.44
**OTHER METABOLISMS (7)**					
307105635	Acetyl-CoA carboxylase α subunit^*^	ACCA	Chl	Fatty acid biosynthesis	2.04 ± 0.29
300266884	Acetyl-CoA carboxylase α subunit^*^	ACCA	Chl^#^	Fatty acid biosynthesis	1.96 ± 0.57
132270939	Acetyl-CoA carboxylase β subunit	ACCB	Chl	Fatty acid biosynthesis	1.80 ± 0.45
158274018	Cytochrome b5 protein	CYB5	ER^#^	Fatty acid biosynthesis	2.99 ± 0.06
19879330	Nucleoside diphosphate kinase	NDK	Mit	Pyrimidine metabolism	1.77 ± 0.18
300266723	5'-Adenylylsulfate reductase^*^	APR	Chl, Mit	Sulfur metabolism	3.33 ± 1.78
158277365	Glycine cleavage system, T protein	GCST	Mit	Photorespiration	1.54 ± 0.51
**TRANSCRIPTION (2)**					
307104400	G-patch domain-containing protein^*^	G-patch	Nuc	RNA processing	2.20 ± 0.69
300260582	DEAD-box helicases^*^	DBH^1^	Nuc^#^	RNA processing	1.68 ± 0.53
**PROTEIN SYNTHESIS (7)**					
300269048	Ribosomal protein S3^*^	RPS3	Nuc	Protein synthesis	1.50 ± 0.12
246880769	Ribosomal protein S7	RPS7	Chl	Protein synthesis	2.26 ± 0.92
158282426	Ribosomal protein S8	RPS8	Chl^#^	Protein synthesis	2.01 ± 0.45
246880750	Ribosomal protein S19	RPS19	Chl	Protein synthesis	2.57 ± 0.80
246880722	Ribosomal protein L5	RPL5	Chl	Protein synthesis	2.95 ± 0.48
158280854	Ribosomal protein L19	RPL19	Chl^#^	Protein synthesis	2.09 ± 0.74
246880748	Ribosomal protein L23	RPL23	Chl	Protein synthesis	2.34 ± 0.00
**PROTEIN FOLDING AND TRANSPORTING (7)**					
18250906	Heat shock protein 70	HSP70^1^	Chl	Protein folding	1.84 ± 0.36
300264935	Luminal binding protein Bip1	Bip1	ER	Protein folding	10.85 ± 6.56
11131843	Calreticulin	CALR	ER, Sec	Protein folding	17.61 ± 15.04
158280974	Protein disulfide isomerase 1	PDI	ER, Sec	Protein folding	5.71 ± 5.77
307110683	Cyclophilin^*^		Cyt	Protein folding	2.12 ± 0.78
116059330	Preprotein translocase SecY subunit	SecY	Chl^#^	Protein folding	1.91 ± 0.11
300266557	AAA^+^-ATPase^1^*^^		Chl	Protein folding	1.90 ± 0.11
**PROTEIN DEGRADATION (5)**					
300259347	26S proteasome regulatory complex	PRC	Cyt, Nuc	Protein degradation	2.31 ± 0.82
300266918	Peptidase M16^*^	M16	Mit	Protein degradation	2.26 ± 0.56
246880736	ATP-dependent Clp protease proteolytic subunit	CLPP	Chl^#^	Protein degradation	1.89 ± 0.14
144575844	AAA-metalloprotease FtsH, chloroplast precursor	FtsH^1^	Chl	Protein degradation	1.66 ± 0.07
158274577	Membrane AAA-metalloprotease	FtsH^1^	Chl	Protein degradation	1.50 ± 0.22
**FUNCTION UNKNOWN (3)**					
300265561	Hypothetical protein VOLCADRAFT_104196		Nuc	Unknown	17.30 ± 15.88
246880719	Hypothetical chloroplast protein RF1		Chl^#^	Unknown	2.77 ± 1.18
307104420	Hypothetical protein CHLNCDRAFT_138637		Nuc	Unknown	2.29 ± 0.51

a *Database accession number from NCBInr and the functional categories of proteins according to the GO criteria, KEGG and NCBInr database*.

b The name of the proteins identified by LC-MS/MS. Protein names marked with an asterisk (^*^) have been edited by us depending on functional domain searching and similarity comparison according to the Gene Ontology criteria.

c *Protein subcellular localization predicted by software (YLoc, LocTree3, Plant-mPLoc, ngLOC, and TargetP). Pound sign (^#^) indicates the subcellular localizations are predicted based on reference, details in Supplemental Table S2. Chl, chloroplast; Cyt, cytoplasm; ER, endoplasmic reticulum; Mit, mitochondria; Nuc, nucleus; PM, plasma membrane; Sec, secreted*.

d *The molecular function of the identified proteins*.

e *The values are presented as means ± standard deviation of ratio H/L normalized*.

**Table 2:** **Salinity shock-decreased proteins upon palmella formation of ***D. salina*****.

**Gi number^a^**	**Protein name^b^**	**Protein abbreviation**	**Sub. Loc.^c^**	**Protein Function^d^**	**Ratio H/L^e^**
**MEMBRANE AND TRANSPORT (1)**					
158281065	Vacuolar ATP synthase β subunit	V-ATPB	Vac^#^	H^+^ transport	0.56 ± 0.02
**CELL STRUCTURE (2)**					
158282728	Flagellar associated protein	FAP	Cyt^#^	Flagellar assembly	0.39 ± 0.25
284518784	Kinesin-like calmodulin binding protein	KCBP	Cyt	Flagellar assembly	0.46 ± 0.16
**CARBOHYDRATE AND ENERGY METABOLISM (9)**					
290465235	Phosphoglucomutase 1	PGM	Chl	Glycometabolism	0.69 ± 0.10
208463466	Glucose-6-phosphate isomerase	GPI	Cyt	Glycometabolism	0.12 ± 0.07
300260957	Fructose-1,6-bisphosphate aldolase^*^	FBPA	Cyt^#^, Chl	Glycometabolism	0.90 ± 0.65
29650775	Enolase	ENO	Cyt	Glycometabolism	0.33 ± 0.18
290755998	Pyruvate kinase	PK	Cyt, Chl	Glycometabolism/ Pyruvate metabolism	0.63 ± 0.20
61338425	UDP-glucose dehydrogenase	UGDH	Cyt	Starch and sucrose metabolism	0.62 ± 0.05
226524601	Glycosyltransferase family 35 protein	GT35	Chl	Starch and sucrose metabolism	0.16 ± 0.09
333691285	Ribulose phosphate-3-epimerase, chloroplast	RPE	Chl	Pentose phosphate pathway	0.48 ± 0.14
307106735	Transketolase^*^	TK	Chl^#^	Pentose phosphate pathway	0.10 ± 0.11
**PHOTOSYNTHESIS (15)**					
74272689	Chloroplast oxygen-evolving protein 3	OEE	Chl	Oxygen evolution	0.18 ± 0.06
108796935	PS II 47 kDa protein	CP47	Chl	PS II stability	0.68 ± 0.44
226454451	Ferredoxin, chloroplast precursor	Fd	Chl	Electron transport	0.12 ± 0.12
226462438	PEP-utilizing enzyme^*^	PUE	Chl, Cyt	Carbon fixation	0.17 ± 0.21
44890111	Ribulose-1,5-bisphosphate carboxylase/oxygenase small subunit	RuBisCO SSU	Chl	Carbon fixation	0.21 ± 0.27
78058384	Ribulose-1,5-bisphosphate carboxylase/oxygenase small subunit	RuBisCO SSU	Chl	Carbon fixation	0.18 ± 0.21
18461352	Ribulose-1,5-bisphosphate carboxylase/oxygenase large subunit	RuBisCO LSU	Chl	Carbon fixation	0.09 ± 0.10
246880771	Ribulose-1,5-bisphosphate carboxylase/oxygenase large subunit	RuBisCO LSU	Chl	Carbon fixation	0.13 ± 0.14
347516457	Ribulose-1,5-bisphosphate carboxylase/oxygenase large subunit	RuBisCO LSU	Chl	Carbon fixation	0.10 ± 0.12
122226542	Ribulose-1,5-bisphosphate carboxylase/oxygenase large subunit	RuBisCO LSU	Chl	Carbon fixation	0.06 ± 0.06
11134148	Phosphoglycerate kinase	PGK	Chl	Carbon fixation	0.13 ± 0.14
300266390	Fructose-1,6-bisphosphatase	FBPase	Chl	Carbon fixation	0.36 ± 0.19
11467786	Mg-protoporyphyrin IX chelatase	MgC	Chl	Chlorophyll synthesis	0.56 ± 0.29
14582814	Mg-protoporyphyrin IX chelatase subunit I	MgC	Chl	Chlorophyll synthesis	0.51 ± 0.20
158281464	Non-discriminatory gln-glu-trna synthetase	GluRS	Chl^#^	Chlorophyll synthesis	0.27 ± 0.24
**OTHER METABOLISMS (21)**					
307111839	Enoyl acyl carrier protein reductase^*^	EAR	Chl	Fatty acid biosynthesis	0.30 ± 0.16
152957049	Biotin carboxylase	BC	Chl	Fatty acid biosynthesis	0.43 ± 0.16
261362648	Biotin carboxylase	BC	Chl^#^	Fatty acid biosynthesis	0.31 ± 0.15
300257372	Biotin carboxylase^*^	BC	Chl^#^	Fatty acid biosynthesis	0.41 ± 0.17
307103608	Inorganic pyrophosphatase^*^	PPA	Mit, Cyt	Fatty acid biosynthesis	0.19 ± 0.13
144577039	CoA binding domain-containing protein^*^	CoA	Mit	Fatty acid biosynthesis	0.59 ± 0.52
116055622	Argininosuccinate synthase	ASS	Cyt^#^	Amino acid metabolism	0.16 ± 0.18
307103805	Carbamoyl-phosphate synthetase^*^	CPS	Cyt	Amino acid metabolism	0.21 ± 0.15
158282886	S-adenosylmethionine synthetase	SAMS	Cyt	Amino acid metabolism	0.14 ± 0.16
334359307	L,L-diaminopimelate aminotransferase α subunit	DAPAT	Chl	Amino acid metabolism	0.14 ± 0.15
300267382	Threonine synthase	TS	Chl	Amino acid metabolism	0.18 ± 0.22
307107618	Cysteine synthase^*^	CS	Chl	Amino acid metabolism	0.09 ± 0.05
307109471	Phosphoserine aminotransferase^*^	PSAT	Chl	Amino acid metabolism	0.11 ± 0.01
121364	Glutamine synthetase	GS	Mit	Amino acid metabolism	0.52 ± 0.14
158276438	Aspartate semialdehyde dehydrogenase	ASDH	Mit	Amino acid metabolism	0.32 ± 0.21
251826344	1-Deoxy-D-xylulose 5-phosphate synthase	DXPS	Chl	Terpenoid backbone biosynthesis	0.30 ± 0.13
251826346	1-Deoxy-D-xylulose 5-phosphate reductoisomerase	DXR	Chl	Terpenoid backbone biosynthesis	0.22 ± 0.11
223045771	4-Hydroxy-3-methylbut-2-enyl diphosphate reductase	HDR	Chl	Terpenoid backbone biosynthesis	0.24 ± 0.16
158273308	Inosine monophosphate dehydrogenase^*^	IMPDH	Cyt	Purine metabolism	0.26 ± 0.16
226514806	Methylene-tetrahydrofolate dehydrogenase^*^	MTDH	Mit^#^	Metabolism of cofactors and vitamins	0.26 ± 0.21
226518884	Cobalamin synthesis protein cobW^*^	CobW	Cyt^#^	Metabolism of cofactors and vitamins	0.42 ± 0.10
**TRANSCRIPTION (8)**					
307111540	Histone H2A^*^	H2A	Nuc^#^	Chromosome/Nucleosome assembly	0.20 ± 0.06
300269009	Histone H2B	H2B	Nuc	Chromosome/Nucleosome assembly	0.19 ± 0.10
116057937	Histones H3 and H4	H3-H4	Nuc	Chromosome/Nucleosome assembly	0.52 ± 0.09
157043072	Histone H4	H4	Nuc	Chromosome/Nucleosome assembly	0.51 ± 0.16
307105891	Sm protein B	SmPB	Nuc	RNA processing	0.46 ± 0.32
158281072	Nucleolar protein, component of C/D snoRNPs	SnRNP	Nuc	RNA processing	0.25 ± 0.14
158270457	Exon junction complex^*^	EJC	Nuc	RNA processing	0.28 ± 0.12
158274317	DEAD-box helicases^*^	DBH^2^	Nuc	RNA processing	0.56 ± 0.08
**PROTEIN SYNTHESIS (13)**					
158277524	Eukaryotic initiation factor	eIF	Nuc	Protein synthesis	0.40 ± 0.06
158279424	Eukaryotic initiation factor 4A-like protein	eIF4A	Nuc	Protein synthesis	0.30 ± 0.14
158271141	Eukaryotic translation elongation factor 1 alpha 2	EF1A2	Cyt	Protein synthesis	0.18 ± 0.11
158270674	Elongation factor 2	EF2	Cyt	Protein synthesis	0.14 ± 0.01
45356747	Elongation factor Tu	EF-Tu	Chl	Protein synthesis	0.37 ± 0.33
246880727	Elongation factor Tu	EF-Tu	Chl	Protein synthesis	0.20 ± 0.21
13560975	Elongation factor Tu	EF-Tu	Chl	Protein synthesis	0.15 ± 0.17
1173201	Ribosomal protein S14	RPS14	Cyt	Protein synthesis	0.60 ± 0.02
158271646	Ribosomal protein S30	RPS30	Cyt^#^	Protein synthesis	0.09 ± 0.07
144578786	Ribosomal protein L7Ae^*^	RPL7	Nuc	Protein synthesis	0.07 ± 0.01
300266059	Plastid/chloroplast ribosomal protein L7/L12	RPL7/L12	Chl	Protein synthesis	0.28 ± 0.36
246880721	Ribosomal protein L14	RP L14	Chl	Protein synthesis	0.52 ± 0.05
300264335	Nascent polypeptide-associated complex^*^	NAC	Nuc	Protein synthesis	0.12 ± 0.11
**PROTEIN FOLDING AND TRANSPORTING (9)**					
158275086	T-complex protein 1 β subunit	TCP	Cyt	Protein folding	0.33 ± 0.19
158271809	Heat shock protein 70A	HSP70^2^	Cyt	Protein folding	0.23 ± 0.12
219766593	Heat shock protein 70A^*^	HSP70^3^	Cyt	Protein folding	0.19 ± 0.15
158274866	SecA protein	SecA	Chl^#^	Protein folding	0.61 ± 0.05
300268035	GroEL-like type I chaperonin^*^	GroEL	Mit	Protein folding	0.59 ± 0.50
158270541	Chaperonin 60A	CPN60^1^	Chl	Protein folding	0.18 ± 0.09
158272007	Chaperonin 60B1	CPN60^2^	Chl	Protein folding	0.07 ± 0.03
226515307	Chaperonin 60B	CPN60^3^	Chl	Protein folding	0.06 ± 0.04
300262179	AAA^+^-ATPase^2^^*^		Chl	Protein folding	0.65 ± 0.11
**PROTEIN DEGRADATION (2)**					
144580847	Ubiquitin^*^	Ub	Nuc	Protein degradation	0.31 ± 0.22
302393778	Polyubiquitin	pUb	Nuc	Protein degradation	0.25 ± 0.18
**ROS SCAVENGING (2)**					
327506370	2-Cys peroxiredoxin	PrxR	Cyt	ROS homeostasis	0.38 ± 0.21
225322932	Ascorbate peroxidase	APX	Cyt^#^	ROS homeostasis	0.54 ± 0.08
**SIGNALING (5)**					
300266536	Mitogen-activated protein kinase^*^	MAPK	Cyt^#^, Nuc	MAPK signaling	0.56 ± 0.11
1421816	Calmodulin-like protein	CLP	Cyt	Calcium signaling pathway	0.36 ± 0.11
157062258	14-3-3 protein	14-3-3^1^	Cyt	Signal transduction	0.18 ± 0.13
227471982	14-3-3 protein^*^	14-3-3^2^	Cyt	Signal transduction	0.17 ± 0.14
333691281	Protein phosphatase 1	PP1	Cyt, Nuc	Dephosphorylation	0.07 ± 0.04
**CELL DIVISION, DIFFERENTIATION AND FATE (1)**					
226462233	Cell division cycle protein 48	CDC48	Cyt	Cell division	0.33 ± 0.37

a *Database accession number from NCBInr and the functional categories of proteins according to the GO criteria, KEGG and NCBInr database*.

b The name of the proteins identified by LC-MS/MS. Protein names marked with an asterisk (^*^) have been edited by us depending on functional domain searching and similarity comparison according to the Gene Ontology criteria.

c *Protein subcellular localization predicted by software (YLoc, LocTree3, Plant-mPLoc, ngLOC, and TargetP). Pound sign (^#^) indicates the subcellular localizations are predicted based on references, details in Supplemental Table S2. Chl, chloroplast; Cyt, cytoplasm; ER, endoplasmic reticulum; Mit, mitochondria; Nuc, nucleus; PM, plasma membrane; Sec, secreted*.

d *The molecular function of the identified proteins*.

e *The values are presented as means ± standard deviation of ratio H/L normalized*.

In addition, mitochondria ATP synthase, NADH:ubiquinone oxidoreductase, and glycine cleavage system T protein were accumulated in cells at 6 HAS (Table 1). However, nine chloroplast- and/or cytoplasm- located proteins involved in pentose phosphate pathway, glyco-metabolism, pyruvate metabolism, and starch/sucrose metabolism were decreased at 6 HAS (Table 2). Among 36 photosynthetic SRPs, those in charge of light harvesting, PS I stability, electron transport, and ATP synthesis were increased, but the majority of proteins in carbon fixation and chlorophyll synthesis were decreased (Tables 1, 2), which were in line with the changes of photosynthesis and chlorophyll content upon palmella formation. Furthermore, 28 other metabolism-related SRPs were mainly involved in fatty acid biosynthesis, amino acid metabolism, terpenoid backbone biosynthesis, vitamins metabolism, purine and pyrimidine metabolism, and sulfur metabolism (Tables 1, 2).

Additionally, the protein profiles revealed the transcription, translation, as well as protein processing and fate were altered upon palmella formation. Four decreased histones and four RNA processing-related proteins indicate the transcription was inhibited upon palmella formation (Table 2). Besides, 20 SRPs involved in chloroplastic or cytoplastic protein synthesis (Tables 1, 2). In addition, the changes of 16 protein processing-related proteins imply that the protein folding, processing and transport were altered by salinity shock (Tables 1, 2). Furthermore, five protein degradation-related enzymes were increased, which were localized in cytoplasm, chloroplast, or mitochondria, respectively (Tables 1, 2). Besides, five signaling proteins and one cell division-related protein were decreased upon palmella formation (Table 2).

### Phosphoproteomic changes during palmella formation

**Figure 6:**
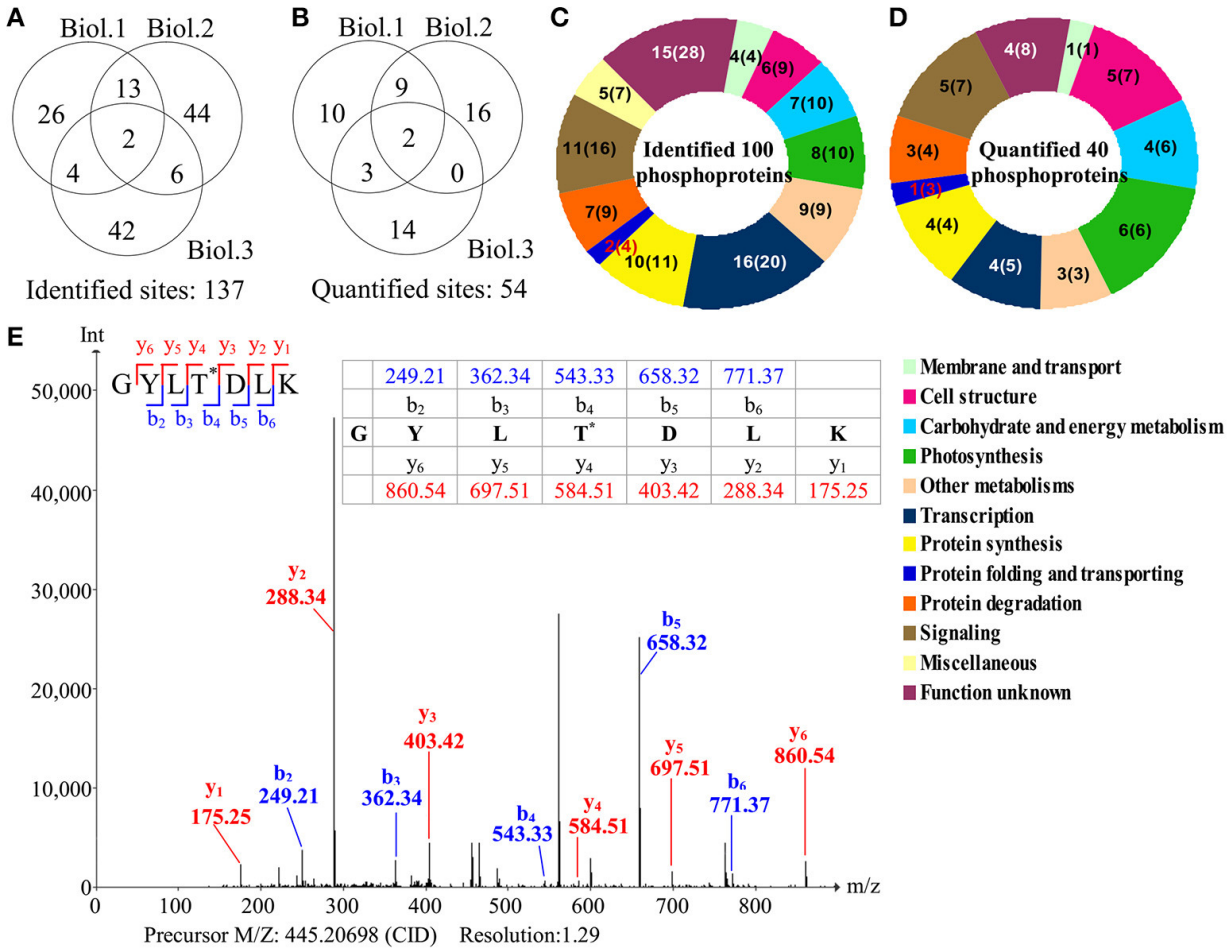
**Overview of phosphoproteome upon palmella formation of ***Dunaliella salina***. (A)** Overlap of phosphorylation sites among three biological replicates. All phosphorylated sites having a reported localization probability of at least 0.75 were considered to be assigned to a specific residue, and we refer to these as phosphorylated sites. 137 phosphorylated sites were identified. **(B)** Among the 137 identified sites, 54 phosphorylated sites were quantified. Three biological replicates for each sample were performed by LTQ-Orbitrap Velos MS/MS analysis, and the obtained results were given in Supplemental Table S3. We compared the overlap of identified phosphorylated sites and quantified phosphorylated sites among the three biological replicates. Biol.1/2/3 represent the first/second/third biological replicate. **(C)** Functional category of 100 identified phosphoproteins (containing 137 identified phosphorylated sites) in *D. salina*. The numbers inside the parentheses shows how many phosphorylated sites identified in phosphoproteins. **(D)** Functional category of 40 quantified phosphoproteins (containing 54 quantified phosphorylated sites). The numbers inside the parentheses show how many phosphorylated sites quantified in phosphoproteins. **(E)** A representative MS/MS spectra of 35 salinity-responsive phosphoproteins. The peptide GYLTDLK from PRP1 splicing factor (gi|307110542).

To investigate the changes of phosphoproteins upon palmella formation, stable isotope dimethyl labeling-based MS approaches were applied to analyze phosphoproteins, 137 phosphorylation sites were identified (Figure 6A), representing 100 phosphoproteins in 12 functional categories (Figure 6C). Among them, 54 phosphorylation sites were quantified (Figure 6B), representing 40 unique phosphoproteins (Figure 6D and Supplemental Tables S3, S4). The amino acid sequences of the 137 identified and 54 quantified phosphorylation sites were shown in Supplemental Table S4. The number of phosphopeptides or phosphorylation sites significantly changed at least a 1.5-fold in three replicates were shown in Supplemental Table S5. Among quantified phosphoproteins, photosynthesis (13%), signaling (13%) and cell structure (13%) were over-represented (Figure 6D). Finally, 35 salinity-responsive phosphoproteins (SRPPs) including 14 salinity-increased phosphoproteins and 22 salinity-decreased phosphoproteins were examined in *D. salina* during palmella formation (Figure 6E, Table 3, Supplemental Table S6, and Supplemental Figure S3). They were involved in photosynthesis, carbohydrate and energy metabolism, other metabolisms, transcription, protein synthesis, protein folding, protein degradation, signaling, and cell structure (Table 3 and Supplemental Table S6).

**Table 3:** **Salinity shock-responsive phosphoproteins upon palmella formation of ***D. salina*****.

**Gi number^a^**	**Protein name^b^**	**Protein abbreviation**	**Sub. Loc.^c^**	**Protein function^d^**	**Sequence with modification^e^**	**Ratio H/L^f^**
**SALINITY-INCREASED PHOSPHOPROTEINS (14)**						
**Cell structure (1)**						
1279362	Striated fiber-assemblin	SFA	Cyt	Flagellar assembly	SSVL**pT^24^**TTGSAIK	5.49 ± 0.86
					S**pS^21^**VLTTTG**pS^28^**AIK	3.66
**Photosynthesis (2)**						
333691283	PSII Light-harvesting chlorophyll a/b binding protein	LHCb^3^	Chl	Light harvesting	VDG**pT^181^**GPAGFSPGVGK	2.18 ± 0.24
					FRVDGTGPAGFSPGVGK	2.07 ± 0.06
246880744	ATP synthase CF1 α subunit	CF1-ATPA	Chl^#^	ATP synthesis	AIEAAAPGIV**pS^139^**R	2.04
**Other metabolisms (2)**						
307106155	Chalcone and stilbene synthases^*^	CHS/SS	Chl^#^	Fatty acid biosynthesis	CF**pS^6^**TIR	1.76
227477517	Glutamine synthetase^*^	GS	Cyt	Amino acid metabolism	HET**pS^331^**SMNDFSWGVANR	1.69
**Transcription (1)**						
300261198	RNA polymerase subunit 8^*^	RPB8	Nuc	Transcription	FSLVLAWTLNLDA**pT^72^**PG**pS^75^**EK	2.07 ± 0.31
**Protein synthesis (4)**						
158283213	Ribosomal protein S3a	RPS3a	Cyt^#^	Protein synthesis	VFEV**pS^69^**LADLQK	2.74
158276036	Ribosomal protein S6	RPS6	Cyt, Nuc	Protein synthesis	KGEQELPGL**pT^127^**DEEKPR	1.56
246880774	Ribosomal protein S9	RPS9	Chl	Protein synthesis	EFPIEN**pS^140^**^*o*^MEESSK	2.08 ± 0.04
158274884	Ribosomal protein L12	RPL12	Cyt	Protein synthesis	VTGGEVGAAS**pS^27^**LAPK	10.35 ± 3.18
**Protein degradation (3)**						
307111867	Lysine motif^*^	LysM	Nuc	Protein degradation	QHA**pT^2264^**EAFAEAWGQQEQQQQQQQQHPELHI**pS^2290^**ADALK	8.82 ± 4.53
300256553	Ubiquitin-conjugating enzyme E2^*^	UBC	Nuc, Cyt	Protein degradation	LF**pS^6^**VKDK	3.62
158279575	Membrane AAA-metalloprotease	FtsH^2^	Chl	Protein degradation	GCLLVGPPG**pT^304^**GK	1.77
**Function unknown (1)**						
226455999	Predicted protein		−	Unknown	F**pS^52^**IM**pT^55^**ALYFAP**pS^62^**ALVPLVAGGV**pS^73^**SLPK	1.57
**SALINITY-DECREASED PHOSPHOPROTEINS (22)**						
**Cell structure (3)**						
226524137	Flagellar associated protein	FAP^2^	Cyt^#^	Flagellar assembly	SL**pS^341^**VEEK	0.65
284518784	Kinesin-like calmodulin binding protein	KCBP	Cyt	Flagellar assembly	AGG**pS^581^**ALGLAQANFGPK	0.50
2625154	Tubulin α chain	TUA	Cyt	Cytoskeleton microtubule	TIQFVDWCP**pT^349^**GFK	0.21
**Carbohydrate and energy metabolism (3)**						
290465235	Phosphoglucomutase 1	PGM	Chl	Glycometabolism	HYGGIIMSASHNPGGPEGDFGIK	0.28 ± 0.00
					HYGGII^*o*^MSASHNPGGPEGDFGIK	0.42 ± 0.03
					KHYGGIIMSApS^163^HNPGGPEGDFGIK	0.56
208463466	Glucose-6-phosphate isomerase	GPI	Cyt	Glycometabolism	DLTSPLHTFEASTLD^*o*^MPTGR	0.34 ± 0.24
300259927	Phosphomannose isomerase type I^*^	PMI	Chl	Glycometabolism	AL**pS^98^**IQSHPDK	0.45
**Photosynthesis (3)**						
246880776	PSII protein H	PsbH	Chl	PS II stability	NS**pT^8^**QTSTSQEPGIVTPLGTLLRPLNSEAGK	0.34 ± 0.08
158277740	Pyruvate phosphate dikinase	PPDK	Chl	Carbon fixation	G^*o*^MYSAEGVLCQLGG^*o*^MTSHAAVVAR	0.38
					G^*o*^MYSAEGVLCQLGG^*o*^M**pT^506^**SHAAVVAR	0.76 ± 0.37
1173346	Sedoheptulose-1,7-bisphosphatase	SBPase	Chl	Carbon fixation	TASCAGTACVN**pS^123^**FGDEQLAVDMVADK	0.15 ± 0.18
					TASCAGTACVN**pS^123^**FGDEQLAVD^*o*^MVADK	0.17 ± 0.23
**Other metabolisms (3)**						
307109275	N-Acyltransferase^*^	NAT	Chl	Fatty acid biosynthesis	GAR**pS^32^**PPR	0.22
227477517	Glutamine synthetase^*^	GS	Cyt	Amino acid metabolism	HETSS^*o*^MNDFSWGVANR	0.37
					HETSSMNDFSWGVANR	0.11 ± 0.09
283139174	Glutamine synthetase II	GSII	Cyt	Amino acid metabolism	HETSS^*o*^MDDFSWGVANR	0.21
**Transcription (3)**						
226455398	Set domain protein	SET	Nuc^#^	Chromosome/Histone modification	ATAPFPPDEP**pS^138^**R	0.26 ± 0.00
300263278	DEAD-box helicases^*^	DBH^3^	Nuc	RNA processing	CHACVGG**pT^147^**SVR	0.13 ± 0.06
307110542	PRP1 splicing factor^*^	PRP1	Nuc	RNA processing	GYL**pT^255^**DLK	0.62 ± 0.11
**Protein folding and transporting (1)**						
300266633	Prefoldin β subunit^*^	PFDb	Nuc	Protein folding	RSA**pS^768^**SLLS**pS^773^**EV**pS^776^**R	0.01
					RSASSLLS**pS^773^**EV**pS^776^**R	0.01
**Signaling (4)**						
158271168	Snf1-like protein kinase	SnRK	Cyt^#^, Nuc	Signal transduction	FQ**pS^173^**APG**pS^177^**R	0.04
300256509	Bsu 1 phosphatase^*^	BSU	Cyt, Nuc	Brassinosteroid signaling	QL**pS^497^**IDQLDNEGR	0.14
300266429	Src homology 2^*^	SH2	Cyt^#^	Phosphotyrosine signaling	DSFDVTVEMLMQQQQPLQYPLQR	0.34
307105164	Glycogen synthase kinase 3^*^	GSK3	Cyt^#^	MAPK signaling	GEPNIS**pY^251^**ICSR	0.49 ± 0.14
**Function unknown (2)**						
226457710	Predicted protein		−	Unknown	GT**pT^109^pS^110^**SQK	0.34
					GT**pT^109^**SSQK	0.61
300267726	Hypothetical protein VOLCADRAFT_86882		Nuc	Unknown	GT**pS^539^**TPDVR	0.34

a *Database accession number from NCBInr and the functional categories of proteins according to the GO criteria, KEGG and NCBInr database*.

b The name of the proteins identified by LC-MS/MS. Protein names marked with an asterisk (^*^) have been edited by us depending on functional domain searching and similarity comparison according to the Gene Ontology criteria.

c *Protein subcellular localization predicted by software's (YLoc, LocTree3, Plant-mPLoc, ngLOC, and TargetP). Pound sign (^#^) indicates the subcellular localizations are predicted based on references, details in Supplemental Table S6. Dash (-) indicate no results were found by software or based on references. Chl, chloroplast; Cyt, cytoplasm; Nuc, nucleus*.

d *The molecular function of the identified proteins*.

e *Peptide sequence with modifications. Bold values show the phosphorylation sites. pX, X is the phosphorylation site. ^o^X, X is the oxidation site. The numbers inside the parentheses show the site location in identified protein sequence*.

f *The values are presented as means ± standard deviation of ratio H/L normalized*.

### Phosphoprotein 3D structure homology model analysis

**Figure 7:**
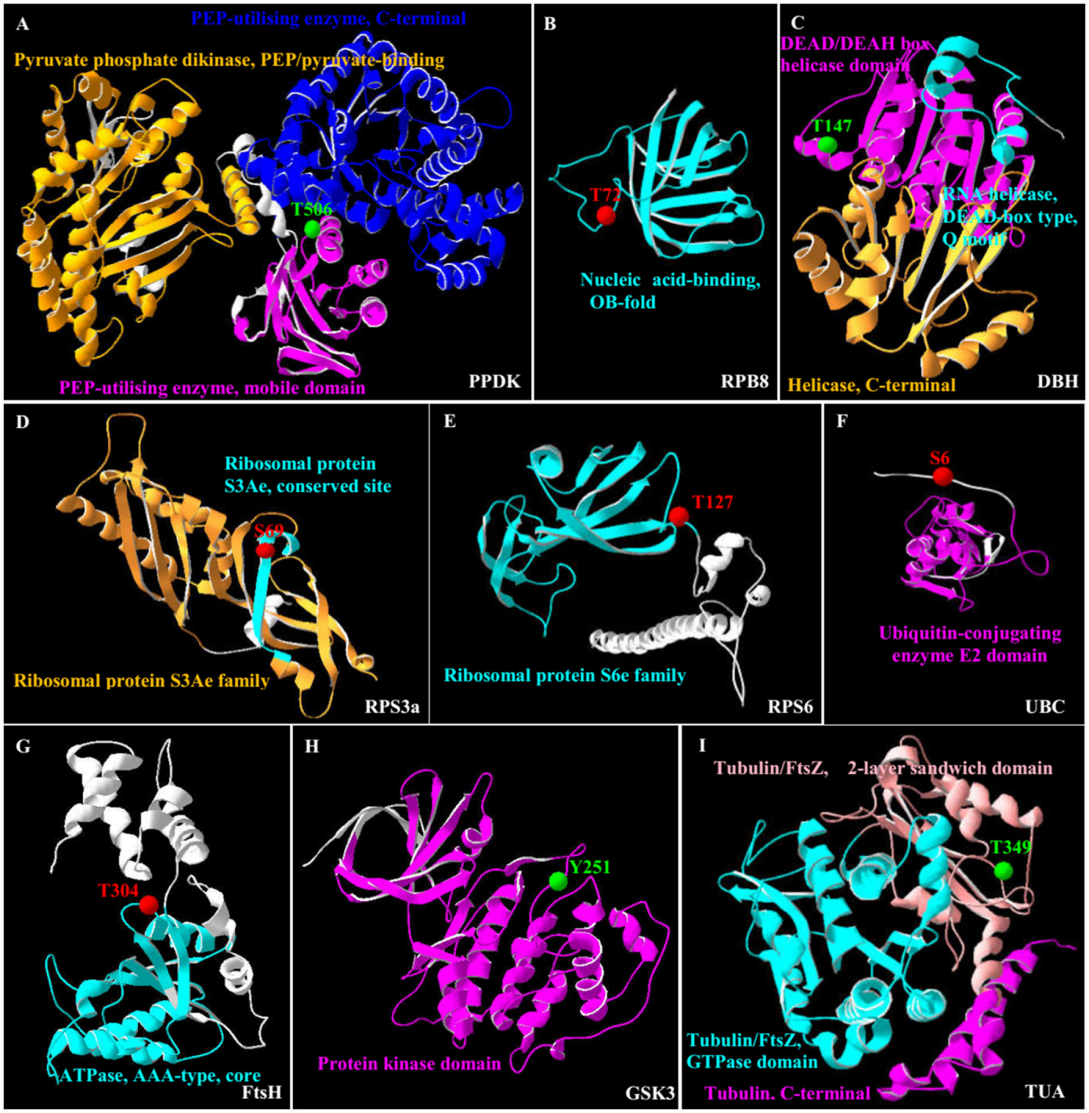
**3D structure of nine salinity-responsive phosphoproteins**. The protein 3D structure model were pre-calculated in SWISS-MODEL workspace (https://swissmodel.expasy.org/) (Arnold et al., 2006) and more detail see in Supplemental Table S7. For 35 salinity-responsive phosphoproteins, nine phosphoproteins were built the most suitable protein structure models. **(A)** Pyruvate phosphate dikinase (PPDK). **(B)** RNA polymerase subunit 8 domain (RPB8). **(C)** DEAD-box helicases (DBH). **(D)** Ribosomal protein S3a (RPS3a). **(E)** Ribosomal protein S6 (RPS6). **(F)** Ubiquitin-conjugating enzyme E2 (UBC). **(G)** Membrane AAA-metalloprotease (FtsH). **(H)** Glycogen synthase kinase 3 (GSK3). **(I)** Tubulin α chain (TUA). The red/green balls showed the increased/decreased phosphorylation sites, respectively, and different colorful ribbon showed the different domain.

In this study, all the 35 SRPPs were subjected to SWISS-MODEL database (https://swissmodel.expasy.org) for building the 3D structure homology model. Among them, nine homologs of SRPPs were aligned to their 3D structure homology models (Figure 7 and Supplemental Table S7).

Importantly, the 3D structure clearly indicated that the phosphorylation sites of eight proteins were localized in the region of function domain (Figure 7), presenting useful information for underlying their specific functions. For pyruvate phosphate dikinase (PPDK), T506 was localized in beta turn, and was part of the phosphoenolpyruvate (PEP)-utilizers enzyme mobile domain (IPR008279), which was a “swiveling” beta/beta/alpha domain for catalyzing the transfer of a phosphoryl group from PEP to a histidine residue (Figure 7A). In RNA polymerase subunit 8 (RPB8), T72 was localized in OB-fold nucleic acid binding domain (IPR012340), which was a part of the five-stranded beta-barrel structure for nucleic acid recognition (Figure 7B). Besides, salinity-decreased T147 was located in DEAD-box helicases (DBH), which was part of DEAD/DEAH box helicase domain involved in various aspects of RNA metabolism (Figure 7C). For ribosomal proteins, the salinity-induced S69 of ribosomal protein S3a (RPS3a) was localized in beta sheet (Figure 7D), while T127 of ribosomal protein S6 (RPS6) was located in beta turn, which were all the conserved site of ribosomal protein S3Ae/S6e family (IPR018281/IPR001377) (Figure 7E). The salinity-induced T304 of membrane AAA-metalloprotease (FtsH) was located in beta turn of AAA^+^ domain (IPR003959), which has conserved alpha-beta-alpha core structure and walker A and B motifs of P-loop NTPase (Figure 7G). The salinity-induced Y251 of glycogen synthase kinase 3 (GSK3) was localized in protein kinase domain (IPR003959), which catalyzes the transfer of the gamma phosphate from ATP to one or more amino acid residues in a protein substrate, resulting in a conformational change affecting protein function (Figure 7H). In addition, salinity-decreased T349 phosphorylation of α-tubulin localized in the carboxy-terminal region which was the alpha domain interface region for motor protein binding (Figure 7I and Supplemental Figure S4). However, the salinity-induced S6 phosphorylation of ubiquitin-conjugating enzyme E2 (UBC) localized outside of its conserved function domain (Figure 7F). Whether the increase of S6 phosphorylation of UBC has an effect on conjugation of ubiquitin to the target protein needs to be confirmed.

### Prediction of salinity-responsive protein–protein interaction upon palmella formation

**Figure 8:**
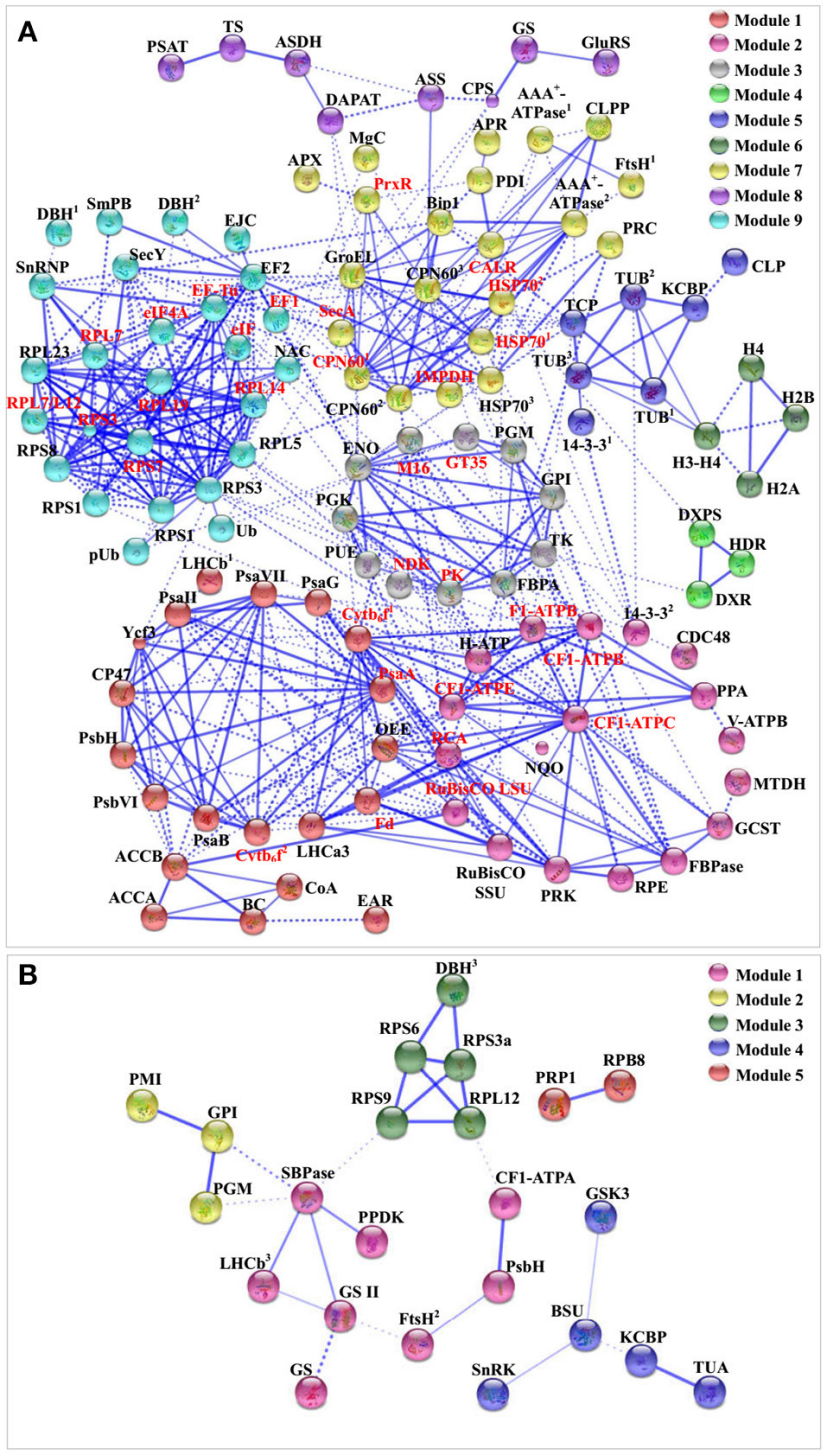
**The protein–protein interaction (PPI) network in ***D. salina*** revealed by STRING analysis. (A)** A total of 151 salinity-responsive proteins represented by 118 unique homologous proteins from *Arabidopsis* are shown in PPI network. Nine main groups are indicated in different colors. **(B)** A total of 35 salinity-responsive phosphoproteins represented by 23 unique homologous proteins from *Arabidopsis* are shown in PPI network. Five main groups are indicated in different colors. The PPI network is shown in the confidence view generated by STRING database. Stronger associations are represented by thicker lines. The abbreviations are referred to Tables 1–3.

To discover the relationship of the 151 SRPs, the PPI networks were generated using the web-tool STRING10 (http://www.string-db.org). After BLASTing in TAIR database (http://www.arabidopsis.org/Blast/index.jsp), 135 homologs in *Arabidopsis* of the 151 SRPs were analyzed, and then subjected to the molecular interaction tool of STRING 10 for creation of proteome-scale interaction network (Supplemental Table S8). Among them, 118 proteins were depicted in the STRING database, and illuminated in nine functional modules with tightly-connected clusters (stronger associations represented by thicker lines) in the network (Figure 8A). Besides, 23 SRPPs were depicted in the STRING database (Figure 8B and Supplemental Table S9). The relationship of proteins in various modules indicates that signaling and protein synthesis/processing/turnover are crucial for the modulation of light harvesting, carbon assimilation and energy apply, as well as the cytoskeleton upon palmella formation. Importantly, the reverse phosphorylation of proteins plays important roles in regulation of proteins interactions.

## Discussion

### Salinity shock-induces cell morphology changes upon palmella formation

*D. salina* has the ability to grow over an extremely wide range of salinity from less than sea water to NaCl saturation (Montoya and Olivera, 1993). The normal cells of *D. salina* are ellipsoid to fusiform, without cell wall, but covered with mucilaginous glycocalyx. The biflagellate motile cells contain a single large cup-shaped posterior chloroplast (Borowitzka and Siva, 2007). The osmotic shock, such as rapid decrease/increase of salinity, would induce palmella formation of *D. salina* (Preetha et al., 2012). The cells in palmella stage lose their flagella and eyespot, become round, and excrete a slime layer to format a multicellular aggregated colonies within a common mucilage (Montoya and Olivera, 1993).

**Figure 9:**
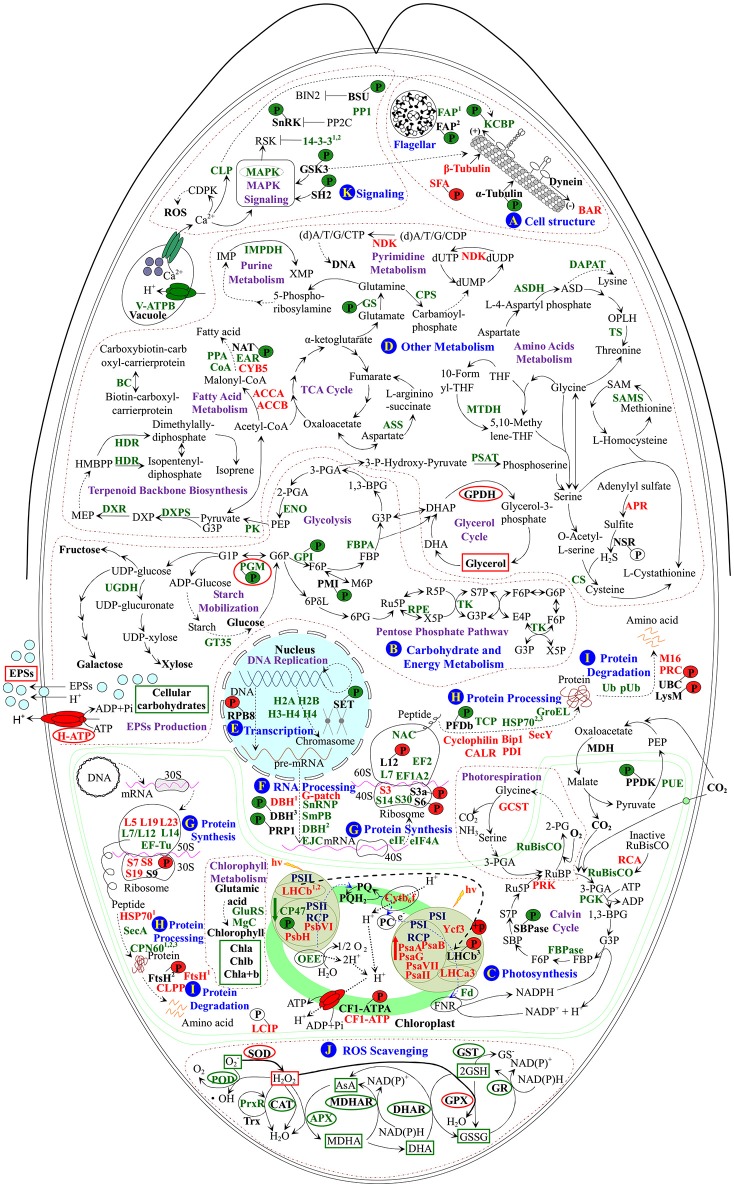
**Schematic presentation of the ***D. salina*** palmella formation mechanisms**. The identified proteins were integrated into subcellular pathways. **(A)** Cell structure. **(B)** Carbohydrate and energy metabolism. **(C)** Photosynthesis. **(D)** Other metabolisms. **(E)** Transcription. **(F)** RNA Processing. **(G)** Protein synthesis. **(H)** Protein processing. **(I)** Protein degradation. **(J)** ROS scavenging. **(K)** Signaling. Protein expression patterns, phosphoprotein expression patterns, enzyme activities, and substrate contents are marked with words, *P* with circles, ellipse, and squares in black (unchanged), red (increased), and green (decreased), respectively. The solid line indicates single-step reaction, and the dashed line indicates multistep reaction. The abbreviations of protein names are referred to Tables 1–3. Other abbreviations used are: 1,3-BPG, 1,3-bisphosphoglycerate; 2-PG, 2-phosphoglycolate; 2-PGA, 2-phosphoglycerate; 30S, chloroplast small ribosomal subunit; 3-PGA, 3-phosphoglycerate; 40S, eukaryotic small ribosomal subunit; 50S, chloroplast large ribosomal subunit; 60S, eukaryotic large ribosomal subunit; 6PG, 6-phosphoguconate; 6PδL, 6-phosphoglucono-δ-lactone; ADP, adenosine diphosphate; AsA, ascorbate; ASD, aspartate-4-semialdehyde; ATP, adenosine triphosphate; BIN2, brassinosteroid insensitive 2; CAT, catalase; CDPK, calcium-dependent protein kinase; CF1-ATPA, ATP synthase CF1 α subunit; Chla/b/a+b, chlorophyll a/b/a+b; (d)A/T/G/C/UDP, (deoxy)adenosine/thymidine/guanosine/deoxycytidine/uridine 5′-diphosphate; (d)A/T/G/C/UTP, (deoxy)adenosine/thymidine/guanosine/deoxycytidine/uridine 5'-triphosphate; DHA, hydroascorbate; DHAP, dihydroxyacetone phosphate; DHAR, dehydroascorbate reductase; DNA, deoxyribonucleic acid; dUMP, deoxy-uridine monophosphate; DXP, 1-deoxy-D-xylulose 5-phosphate; E4P, erythrose 4-phosphate; EPSs, exopolysaccharides; F6P, fructose 6-phosphate; FBP, fructose-1,6-bisphosphate; FNR, ferredoxin-NADP^+^ reductase; G1P, glucose-1-phosphate; G3P, glyceraldehyde 3-phosphate; G6P, glucose-6-phosphate; GPDH, glycerol-3-phosphate dehydrogenase; GPX, glutathione peroxidase; GR, glutathione reductase; GSH, reduced glutathione; GSSG, oxidized glutathione; GST, glutathione S-transferase; HMBPP, 1-hydroxy-2-methyl-2-butenyl 4-diphosphate; hv, light energy; IMP, inosine-5′-monophosphate; M6P, D-mannose 6-phosphate; MDH, malate dehydrogenase; MDHA, monohydroascorbate; MDHAR, monodehydroascorbate reductase; MEP, 2-C-methyl-D-erythritol 4-phosphate; mRNA, messenger ribonucleic acid; NSR, nitrite/sulfite reductase; OPLH, O-phospho-L-homoserine; P, phosphorylation site; PC, plastocyanin; PEP, phosphoenolpyruvate; Pi, inorganic phosphate; POD, peroxidase; PP2C, protein phosphatase 2C; PQ, plastoquinone; PQH_2_, reduced plastoquinone; pre-mRNA, precursor messenger ribonucleic acid; PSI, photosystem I; PSII, photosystem II; PSII-RCP, photosystem II reaction center protein; PSI-RCP, photosystem I reaction center protein; R5P, ribose-5-phosphate; ROS, reactive oxygen species; RSK, ribosomal S6 kinase; Ru5P, ribulose 5-phosphate; RuBP, ribulose-1,5-bisphosphate; S7P, sedoheptulose 7-phosphate; SAM, S-adenosylmethionine; SBP, sedoheptulose-1,7-bisphosphate; SOD, superoxide dismutase; TCA, tricarboxylic acid; THF, tetrahydrofolate; Trx, thioredoxin; X5P, xylulose-5-phosphate; XMP, xanthosine-5′-phosphate.

Upon palmella formation, a salt-induced Bin/Amphiphysin/Rvs domain-containing protein would facilitate to bind membrane for membrane curvature modulation in *D. salina* (Figure 9A and Table 1; Rao and Haucke, 2011). Osmotic and salt stresses cause microtubule disassembly and reorganization (Komis et al., 2002; Shoji et al., 2006). In this study, the salt-induced β-tubulin implies that the tubulin accumulation in cytoplasmic pool of palmella cells may prepare for the flagella reformation of free-swimming cells (Figure 9A), while the salinity-decreased phosphorylation of T349 would facilitate the binding of α-tubulin with motor proteins to regulate the microtubule stability upon palmella formation (Figures 7I, 9A, and Supplemental Figure S4; Ban et al., 2013; Wang et al., 2013).

We found the flagella formation was modulated in response to salinity (Figure 9A). Flagellar associated proteins function as a molecular chaperon, and their declined abundance and phosphorylation level would contribute to the flagella lost during the palmella formation. Consistently, the abundance and phosphorylation at S581 of kinesin-like calmodulin binding protein (KCBP) were also decreased, but the normalized phosphorylation was unchanged upon palmella formation (Figure 9A). KCBP was tightly connected with tubulin in PPI network (Figure 8). The highly conserved KCBP is mainly localized near the basal body in *D. salina* (Shi et al., 2013) and *C. reinhardtii* (Dymek et al., 2006), which has a myosin tail homology-4 region in the N-terminal tail and a calmodulin-binding region following the motor domain (Dymek et al., 2006). KCBP plays an important role in microtubule-based intracellular motility. Therefore, the decrease of the abundance and phosphorylation of KCBP implies its motor activity may be inhibited upon palmella formation (Figure 9A and Table 3), but we cannot confirm its activity was regulated by phosphorylation. In addition, the increased abundance and phosphorylation level of striated fiber-assemblin would facilitate to regulate the flagellar root microtubule stability upon palmella formation (Figure 9A; Lechtreck et al., 2002).

### Accumulation of exopolysaccharides and ROS homeostasis are crucial upon palmella formation

The *Dunaliella* cells were enclosed in a thin elastic plasma membrane surrounded by mucus “surface coat”, but lack rigid polysaccharide cell wall (Ben-Amotz and Avron, 1990). It is known that accumulation of glycerol in cells was crucial for osmotic homeostasis in *D. salina* in response to salinity (Pick, 2002; Liska et al., 2004). In this study, salinity shock-induced accumulation of glycerol and increase of the activity of glycerol metabolic enzyme are important for palmella formation (Figure 2A).

Similarly, total EPS contents were all increased in halotolerant cyanobacterium *Microcoleus vaginatus* (Chen et al., 2006), halotolerant bacterium *Rhodopseudomonas acidophila* (Sheng et al., 2006), and medicinal mushroom *Phellinus linteus* (Zou et al., 2006). In this study, EPS slime layer surrounded *D. salina* cells was enhanced (Figure 1G), due to the content of EPSs was increased upon palmella formation (Figure 2B). The salinity-induced EPSs would facilitate the accumulation of water and the reduction of ion influx, protecting the membrane system upon palmella formation. The EPSs around *D. salina* cells were excreted from cells through plasma membrane sugar transporters, which were energy-dependent H^+^-symporters (Büttner and Sauer, 2000). Its energization was via the proton-motive force generated by the PM H^+^-ATPase, and the modulation of H^+^-ATPase activity would immediately affect the sugar transport kinetics (Doidy et al., 2012). In this study, the abundance and activity of PM H^+^-ATPase were all increased to activate the efflux of sugars upon palmella formation (Figures 2D, 9B, and Table 1; Carpaneto et al., 2010). We also found ten carbohydrate metabolism-related proteins were all salinity-decreased upon palmella formation, which were involved in glycolysis, pentose phosphate pathway, as well as starch mobilization and glucose metabolism, respectively (Figure 9B and Tables 2, 3). The normalized phosphorylation level and activity of PGM were all decreased upon salinity-induced palmella formation (Table 3 and Supplemental Table S1), which implies that the phosphorylation-dependent activity of PGM is inhibited in *D. salina* under salinity-induced oxidative stress. Moreover, the phosphorylation level of glucose-6-phosphate isomerase and phosphomannose isomerase were also reduced upon palmella formation. It has been reported that glycolytic enzymes were oxidized and inactivated when cells were subjected to oxidative stress, leading to a metabolic reshuffling of glucose equivalents through the pentose phosphate pathway for providing a necessary reducing power (NADPH) for antioxidant defense mechanism in cells (Shanmuganathan et al., 2004). These indicate that carbohydrate metabolism may be reduced in cells upon salinity-induced palmella formation, similar to the salinity-decreased total carbohydrates in halotolerant cyanobacterium *M. vaginatus* (Chen et al., 2006).

In addition, ROS homeostasis is crucial for rapid metabolism transition upon salinity-shock palmella formation. In this study, the salinity shock-decreased activities of most ROS scavenging enzymes (i.e., POD, APX, MDHAR, DHAR, GR, and GST), the abundances of 2-Cys peroxiredoxin (PrxR) and APX (Figures 4B–F, 9J and Table 2), as well as the contents of GSSG and GSH (Figure 4H) imply that GSH-AsA cycle, PrxR/thioredoxin pathway, and APX pathway might be inhibited under salinity shock. However, the salinity-increased activities of SOD and GPX indicate that the dismutation of superoxide into oxygen and H_2_O_2_ and subsequently reduction in GPX pathway are enhanced for ROS scavenging upon palmella formation (Figures 4B,E, 9J). Importantly, the abundance of SOD was oxidative stress-increased during the early transition of green vegetative cells to red cysts in *H. pluvialis* (Wang et al., 2004a). Thus, the enhanced SOD and GPX pathways may be the key strategy for ROS homeostasis upon salinity-induced palmella formation.

### Photosynthesis modulation upon palmella formation

Upon salinity shock-induced palmella formation, the photosynthesis of *D. salina* was significantly inhibited, being reflected from the obvious decline of chlorophyll content and photosynthesis oxygen evolution. A similar phenomenon (Figures 1D–G) also occurred upon copper-induced palmella formation of *C. reinhardtii* (Sztrum et al., 2012), and the transformation process of green vegetative cells into red aplanospores of *H. pluvialis* (Scibilia et al., 2015).

The excess light energy absorbance was reduced. The decline of the abundances of chlorophyll biosynthesis-related enzymes (i.e., Mg-protoporyphyrin IX chelatase, and gln-glu-trna synthetase) indicated that the chlorophyll biosynthesis may be inhibited upon palmella formation (Figure 9C and Table 2), which was also observed in *H. pluvialis* in response to nitrogen starvation (Scibilia et al., 2015). Besides, the decrease of chloroplast oxygen-evolving protein and PSII 47 kDa protein would attribute to the salt-inhibited photosynthesis oxygen evolution activity, which was supposed to facilitate the reduction of excess light energy absorbance in palmella cells (Figure 9C and Table 2).

The stability and activity of PSII were modulated by salinity shock. The PSII photochemistry activity [e.g., Fv/Fm, Fv'/Fm', and Y(II)] and linear electron flux were all obviously reduced upon palmella formation, which was similar with what happened in high light-stressed *D. salina* (Gu et al., 2014). However, the abundances of two PSII assembly-related proteins [PSII protein H (PsbH) and PSII protein VI] were increased upon palmella formation (Figure 9C and Table 1). PsbH is a 10-kDa phosphoprotein associated with the inner antenna PSII 47 kDa protein, which is conserved and essential for assembling PSII in algae and higher plants (Summer et al., 1997). The *psbH* mutants appeared a PSII deficient phenotype and lack of a functional PSII complex (Summer et al., 1997). Interestingly, we also found T8 phosphorylation of PsbH was decreased upon palmella formation (Figure 9C and Table 3). However, a T2 knockdown mutant of PsbH in *C. reinhardtii* has similar phonotype of wild-type strains, indicating that T2 phosphorylation of PsbH probably doesn't affect its function (O'Connor et al., 1998). Besides, PSII protein VI is the beta subunit of cytochrome b559 essential for PSII assembly, whose increase would enhance the cyclic electron transport (CET) or in a side path of electron flow for protecting PSII from photoinhibition (Figure 9C and Table 1; Burda et al., 2003).

Enhancement of CET would facilitate ATP synthesis. The salinity-suppressed ETR(II) and salinity-increased ETR(I) abundance implied that CET was tend to enhanced upon palmella formation in *D. salina*. The increased abundances of electron carrier protein cytochrome b_6_/f complex and various PSI complex proteins (i.e., P700 chlorophyll a apoprotein A1, P700 chlorophyll a apoprotein A2, PsaG, PSI reaction center subunit II, PSI subunit VII, assembly protein Ycf3, and light-harvesting chlorophyll a/b protein 3) would contribute to the induced CET (Figure 9C and Table 1). This would facilitate funneling excess electrons to generate ATP without increasing oxygen evolution (Zhang et al., 2010; Gu et al., 2014). Interestingly, the normalized phosphorylation level of light harvesting chlorophyll a/b binding proteins (LHCII) of PSII (LHCb) was enhanced upon palmella formation (Figure 9C and Table 3). The reversible phosphorylation of LHCII is generally considered as an adaptation mechanism to balance energy distribution between PSII and PSI for regulating redox homeostasis in chloroplasts (Grieco et al., 2012). Our proteomic results revealed that the abundances of several enzymes in Calvin cycle were decreased upon salinity shock-induced palmella formation, such as ribulose-1,5-bisphosphate carboxylase/oxygenase (RuBisCO), phosphoglycerate kinase, and fructose-1, 6-bisphosphatase (Figure 9C and Table 2). The photosynthetic CO_2_ fixation was inhibited due to the down-regulation of transcript levels of Calvin cycle enzymes in *D. salina* under high salt stress (Liska et al., 2004). Similarly, the decreased RuBisCO was also examined in *H. pluvialis* during the early transition of green vegetative cells to red cysts under oxidative stress (Wang et al., 2004a). Additionally, the abundances of chloroplastic ribulose phosphate-3-epimerase and transketolase also imply that the Calvin cycle may be inhibited in *D. salina* under salinity shock (Figure 9B and Table 2).

We also found the phosphorylation level of chloroplast-localized PPDK was decreased upon palmella formation (Figure 9C and Table 3), and PPDK was tightly connected with sedoheptulose-1,7-bisphosphatase in PPI network (Figure 8B). PPDK catalyzes the formation of PEP that is the initial acceptor of CO_2_ in C4 pathway. In *U. linza*, the elevated PPDK transcription and enzyme activity enhanced C4 carbon metabolism under high salinity stress (Xu et al., 2013). In this study, the phosphorylation site T506 of PPDK is localized in beta turn of PEP-utilizers enzyme mobile domain (IPR008279) region (Figure 7A). It is reported that the reverse phosphorylation of PPDK (T456 in maize) would regulate the switch of its active (dephosphorylation) and inactive (phosphorylation) states (Chastain et al., 2002). Thus, the decreased phosphorylation at T506 of PPDK (homologous site with maize) would induce its activity, resulting in enhanced C4 pathway activity upon the salinity-induced palmella formation.

### Several basic metabolisms are reduced in dormant palmella cells

The photosynthetic oxygen evolution, PSII activity, and photosynthetic CO_2_ fixation were all reduced after free-swimming cells lost their flagella to be immotile (Figures 1, 3). Additionally, the abundances of several enzymes involved in fatty acid metabolism, amino acids metabolism, terpenoid backbone biosynthesis, and purine/pyrimidine metabolism were decreased (Figure 9D and Tables 2, 3), implying these pathways may be inhibited upon palmella formation. Similarly, proteins involved in hydrophobic fatty acid biosynthesis and amino acid synthesis were decreased in *C. reinhardtii* in response to salt stress for less than 24 h (Mastrobuoni et al., 2012) and nitrogen starvation for 6 h (Longworth et al., 2012). Besides, amino acid metabolism-related genes were down-regulated in brown alga *Ectocarpus siliculosus* under hypersaline conditions (Dittami et al., 2011). In addition, several nitrogen assimilation and pyruvate kinase metabolism-related proteins were decreased during the oxidative stress-induced early transition of green vegetative cells to red cysts in *H. pluvialis* (Wang et al., 2004a). All these indicate that the palmelloid cells become dormant under salinity shock condition. However, the energy production was induced upon palmella formation. This is consistent with what happened during oxidative stress-induced early transition of green vegetative cells to red cysts in *H. pluvialis* (Wang et al., 2004a), as well as in hypersaline-stressed brown alga *E. siliculosus* (Dittami et al., 2011), which is crucial for energy supply in dormant or stressed algae cells.

### Nuclear and chloroplastic gene expression regulation upon palmella formation

The metabolic changes were regulated by gene expression pattern upon palmella formation. We found the abundances of ten transcription-related proteins and phosphorylation level of RPB8 were changed (Figure 9E and Tables 1–3). Three decreased RNA processing-related proteins (i.e., Sm protein B, nucleolar protein snoRNP, and exon junction complex) indicates the pre-mRNA splicing and localization are probably reduced (Figure 9F and Table 2; Tange et al., 2004). However, G-patch domain-containing protein, which functions in RNA recognition, RNA binding or splicing, was increased upon palmella formation (Figure 9F and Table 1). In addition, DBH is involved in various aspects of RNA metabolism (e.g., nuclear transcription, pre-mRNA splicing, and RNA decay) was salinity-altered, In our results, the salinity-reduced DBH is tightly connected with other protein synthesis-related proteins (Figure 8), and its phosphorylation site T147 located in helices (Figure 7C), implying the phosphorylation is perhaps involved in the regulation of DBH function (Figure 9F).

Besides, the phosphorylation at T72 of RPB8 was salinity-induced upon palmella formation, and T72 is localized in OB-fold nucleic acid binding domain (IPR012340) for nucleic acid recognition (Figures 7B, 9E and Table 3). Similarly, the S2 phosphorylation of RNA polymerases (RNA pol) II was increased in wild-type fission yeast under nitrogen starvation (Sukegawa et al., 2011), and RNA pol α subunit were also stress-increased in other algae, such as copper-stressed marine alga *Scytosiphon gracilis* (Contreras et al., 2010), salt-treated *Bifidobacterium longum* NCIMB 8809 (Sánchez et al., 2005), and acid (low pH)-stressed *Streptococcus mutans* (Len et al., 2004). Although the function of RPB8 phosphorylation remains unknown, its increase would affect RNA pol III assembly and catalytic function upon palmella formation of *D. salina* (Voutsina et al., 1999). Additionally, RPB8 was tightly connected with PRP1 splicing factor (PRP1) in PPI network (Figure 8B). Interestingly, the phosphorylation at T255 of PRP1 was decreased upon palmella formation (Figure 9F and Table 3). The N-terminus highly-conserved site of PRP1 can be phosphorylated by PRP4 during the spliceosome activation (Lützelberger et al., 2010). The decline of PRP1 phosphorylation implies that spliceosome activity is probably salinity reduced upon palmella formation.

*De novo* protein synthesis plays an important role in abiotic stress adaptation in plants. We found that eukaryotic initiation factor (eIF), eIF 4A-like protein, elongation factor (EF) 1 alpha 2 and EF2 were all reduced (Figure 9G and Table 2), indicating that translation initiation and peptide elongation are decreased upon palmella formation. Importantly, protein phosphorylation is involved in the initiation of protein translation (Jackson et al., 2010). We found that the phosphorylation levels of RPS6 (T127), RPS3a (S69), and ribosomal protein L12 (RPL12) (S27), and other ribosomal proteins (RPS3 and RPL23) were increased, but the abundances of ribosomal proteins S14 and ribosomal proteins L7 were decreased during palmella formation (Figure 9G and Tables 1–3). Among them, RPS6 phosphorylation in plants leads to the selective recruitment of ribosomal mRNAs to polysomes, regulating in the growth pattern of plants in response to environment changes (Reinbothe et al., 2010). In addition, the phosphorylation sites of RPS6 and RPS3a were all the conserved site of ribosomal protein family (Figures 7D,E). Therefore, the induced phosphorylation of T127 in RPS6 could be responsible for the elevated level of protein synthesis upon palmella formation. Besides, RPS3 is crucial in translation initiation via participation in rearrangements of the 40S subunit structure and promotion of subsequent recognition of the start codon and interaction with mRNA (Valášek, 2012). In our studies, the increased RPS3 abundance and the phosphorylation at S69 of RPS3a would contribute to translation initiation upon palmella formation. In addition, the RPL12 is a member of ribosomal L7/12 stalk, which functions in restoring the biological activity of “core” ribosomal particles (Ilag et al., 2005). Although the phosphorylation of RPL12 was reported, its exact function is still not clear.

### Chloroplast and cytoplasm protein processing and turnover upon palmella formation

Under stress conditions, the misfolded proteins accumulated in algae cells should be refolded or removed. We found that 23 proteins and four phosphoproteins were involved in protein processing and degradation upon palmella formation (Figure 9H and Tables 1–3). The phosphorylation at S768 and S773 of prefoldin β subunit (PFDb) was decreased upon palmella formation (Figure 9H and Table 3). PFD is a cofactor of the group II chaperonins for capturing and transferring the unfolded actin and tubulin for correct folding (Millán-Zambrano and Chávez, 2014). Furthermore, the decrease of cytosolic chaperonin T-complex protein 1 β subunit (TCP) was examined upon palmella formation (Figure 9H and Table 2), which assists in the folding of newly synthesized actin, tubulin, and other polypeptides *in vivo* (Sternlicht et al., 1993). Previous proteomics results have showed that the cold-decreased PFD in cold-tolerant *Zoysia japonica* (Xuan et al., 2013) and salt-decreased TCP in *Thellungiella halophila* (Pang et al., 2010), indicating the protein folding is inhibited under stress. In our studies, the decrease of PFDb phosphorylation and the abundance of TCP might be related with microtubule organization upon palmella formation.

Besides, three endoplasmic reticulum-located chaperones, calreticulin (CALR), protein disulfide isomerase 1, and luminal binding protein Bip1, were all obviously increased upon palmella formation in *D. salina* (Figure 9H and Table 1). CALR is involved in the folding and quality control of newly synthesized proteins and glycoproteins, which is highly conserved and crucial for plant development and stress response (Garg et al., 2015). In addition, protein disulfide isomerase aids in the formation of proper disulfide bonds during protein folding in the endoplasmic reticulum (Appenzeller-Herzog and Ellgaard, 2008), which were salinity-increased in salt-tolerant *Medicago sativa* (Rahman et al., 2015), barley (Mostek et al., 2015), rice (Ghaffari et al., 2014), and halotolerant yeast (*Rhodotorula mucilaginosa*) (Lahav et al., 2004). Additionally, luminal binding protein Bip1 functions in precursor protein import and translocation (Wang et al., 2004b), which was high salt-induced in halotolerant yeast (Lahav et al., 2004). All these imply that salinity shock might enhance the protein folding and importation for maintaining the correct conformation and subcellular location of the oxidative proteins upon palmella formation.

In addition, two cytoplasmic heat shock protein 70s (HSP70) were decreased, but one chloroplast HSP70 was increased upon palmella formation (Figure 9H and Tables 1, 2). HSP70s assists the folding of *de novo* synthesized polypeptides and the translocation of precursor proteins in response to osmotic stress (Wang et al., 2004b), which was reported to increase in salinity-treated *D. salina* (Liska et al., 2004; Katz et al., 2007), and upon oxidative stress-induced early transition from green vegetative cells to red cysts in *H. pluvialis* (Wang et al., 2004a). This implies that the transportation and processing of certain newly synthesized peptide into chloroplasts are enhanced upon palmella formation of *D. salina*. Moreover, the chloroplast-located preprotein translocase SecY subunit is a component of the SecYEG translocon, being driven by the chloroplast ATPase SecA. Both SecY and SecA are essential for protein transportation from cytoplasm to chloroplasts (du Plessis et al., 2011). Their abundance changes may imply that the nuclear encoding proteins are necessary for chloroplasts in salinity shock-stressed *D. salina* (Figure 9H and Tables 1, 2). The increase of cyclophilin in *D. salina* would enhance the protein folding upon palmella formation (Figure 9H and Table 1; Zhang et al., 2012). The decrease of a mitochondrial GroEL and three chloroplastic chaperonin 60s would inhibit protein folding of RuBisCO, and the abundance of chaperonin 60s correlate positively with RuBisCO in plant cells (Figure 9H and Table 2; Holland et al., 1998).

Ubiquitin-dependent selective degradation of proteins is crucial upon palmella formation in *D. salina* (Ciechanover, 1998). In this study, salinity-induced abundance of 26S proteasome regulatory complex, as well as phosphorylation at S6 of UBC indicate that ubiquitin-dependent protein degradation in cytoplasm is enhanced upon palmella formation (Figure 9I and Tables 1, 3). Pervious proteomic study reported the increased 26S proteasome regulatory complex in *D. salina* under long-term salt stress (Liska et al., 2004). The increased phosphorylation at S6 of UBC located in the kinase motifs of CK2 implies that the conjugation of ubiquitin to the target protein may be enhanced (Supplemental Table S6).

The damaged proteins in chloroplasts need to be degraded through an efficient proteolytic system during chloroplast biogenesis, maintenance, and stress response (Ramundo et al., 2014). Upon palmella formation, the abundance and phosphorylation level of FtsH, as well as the abundance of ATP-dependent Clp protease proteolytic subunit were salinity-increased in chloroplasts in *D. salina* (Figure 9I and Tables 1, 3). FtsH can efficiently degrade proteins with low thermodynamic stability, and was increased in short-term salinity-stressed *D. salina*, osmotic shock-treated *Oenococcus oeni* (Bourdineaud et al., 2003), and heat/light-stressed cyanobacterium *Synechocystis* PCC 6803 (Silva et al., 2003). Additionally, FtsH was tightly connected with photosynthesis-related proteins in PPI network (Figure 8B). Therefore, the increase of T304 phosphorylation in AAA^+^ domain of FtsH would facilitate its assembly/disassembly with photosynthetic proteins to perform its peptidase function (Figures 7G, 9I and Table 3). Besides, the chloroplast gene encoding Clp protease is essential for cell viability (Ramundo et al., 2014), which was increased in *D. salina* under long-term salt stress (Katz et al., 2007). All these would enhance the removal of abnormal, modified, and mistargeted proteins in chloroplasts.

The mitochondria peptidase M16 was increased in palmella cells (Figure 9I and Table 1) and in *D. salina* cells under long-term salt stress (Liska et al., 2004). The peptidase M16 was also increased in suspension cells of *A. thaliana* (Ndimba et al., 2005), gametophore of *Physcomitrella patens* (Wang et al., 2008), and roots of *O. sativa* (Li et al., 2010) under salt stress. These results suggest the proteolytic reaction in mitochondrial processing may be important for stress-induced palmella formation.

### Salinity-responsive signaling pathways are reduced upon palmella formation

The palmella formation is regulated by a sophisticate signaling network in *D. salina*. The salinity-decreased protein abundance and/or phosphorylation level imply that several crucial salinity-responsive signaling pathways (e.g., mitogen-activated protein kinase (MAPK) signaling, brassinosteroid (BR) signaling, and Snf1-like protein kinase (SnRK) signaling) tend to be inhibited upon salinity shock-induced palmella formation (Figure 9K and Tables 2, 3). Among them, MAPKs are highly conserved serine/threonine kinases in combination with their upstream activators, which convey osmotic stress signals to appropriate effectors and contribute to adaptation to the high salt stress (Moustafa et al., 2014). Additionally, SH2 domains are crucial for protein docking to phosphorylated tyrosine residues on other proteins, which are common in adapter proteins that aid in the signal transduction of receptor tyrosine kinase pathways (Koytiger et al., 2013). The decrease of MAPK and SH2 domain containing protein indicated that the active salt-responsive MAPK signaling might be inhibited in the dormant palmelloid cells. Moreover, in the MAPK signaling pathway, GSK3 can phosphorylate MAP1B to regulate flagellar length and assembly (Wilson and Lefebvre, 2004). The phosphorylation of Y218 in *C. reinhardtii* GSK3 regulated its active states (Wilson and Lefebvre, 2004). The GSK3 in *D. salina* shared the conserved phosphorylation site with that in *C. reinhardtii*. This conserved phosphorylation site was localized in protein kinase domain (Figure 7H). Thus, the decreased phosphorylation of Y251 in GSK3 of *D. salina* would inhibit its activity to reduce the flagellar stability upon palmella formation.

The salinity-decreased calmodulin-like protein and KCBP would contribute to inhibition of microtubule-binding activity of motor domain during palmella formation of *D. salina* (Shi et al., 2013). The decreased phosphorylation level at S173 and S174 of SnRK indicates that SnRK-related signaling pathway for regulation of ion homeostasis and ROS production may be reduced upon palmella formation (Gong et al., 2002; Diedhiou et al., 2008; Mao et al., 2010; Kulik et al., 2012). The decreased phosphrylation level at S497 of Bsu 1 phosphatase would reduce its activity, leading to the inhibition of nuclear transcription factors to repress BR-responsive gene expression upon palmella formation (Mora-García et al., 2004).

## Conclusions

In the life cycle of unicellular algae, palmella stage is critical for cell surviving in various stress conditions. Salinity-induced palmella formation is a fine-tuned cellular process. By integrating analysis of physiological, quantitative proteomics, and phosphoproteomics data, we revealed the specific molecular mechanisms upon palmella formation (Figure 9). They mainly include (1) cell membrane curvature and cytoskeleton dynamics are modulated for cell morphological changes, (2) accumulations of glycerol and EPSs are enhanced for protection of membrane system, (3) SOD and GPX pathways are specific for ROS scavenging, (4) the activities of photosynthesis oxygen evolution and PSII are inhibited, but the CET and C4 pathway are enhanced, (5) nuclear and chloroplastic gene expression are regulated in response to salinity, and (6) chloroplast and cytoplasm protein processing and turnover are enhanced. All these provide novel insights into the underlying salinity shock-induced palmella formation.

## Author contributions

SW and SD designed research. SW, YB, QZ, CS, KC, ZX, CZ, and SD performed research. HZ, MY, and WM contributed new reagents or analytic tools. SW, QZ, JM, and SD analyzed data. SW, SD, and SC wrote the paper.

### Conflict of interest statement

The authors declare that the research was conducted in the absence of any commercial or financial relationships that could be construed as a potential conflict of interest.

## Abbreviations

3D: three-dimensional
APX: ascorbate peroxidase
AsA: ascorbate
BR: brassinosteroid
CALR: calreticulin
CAT: catalase
CET: cyclic electron transport
DAS: days after salinity shock
DBH: DEAD-box helicases
DHA: hydroascorbate
DHAR: dehydroascorbate reductase
DTT: dithiothreitol
EF: elongation factor
eIF: eukaryotic initiation factor
EPSs: exopolysaccharides
FtsH: membrane AAA-metalloprotease
GPDH: glycerol-3-phosphate dehydrogenase
GPX: glutathione peroxidase
GR: glutathione reductase
GSH: reduced glutathione
GSK3: glycogen synthase kinase 3
GSSG: oxidized glutathione
GST: glutathione S-transferase
HAS: hours after salinity shock
HSP70: heat shock protein 70
KCBP: kinesin-like calmodulin binding protein
IMAC: Ti^4+^-immobilized metal ion affinity chromatography
LC: liquid chromatography
LHCb: light harvesting chlorophyll a/b binding proteins of photosystem II
LHCII: light harvesting chlorophyll a/b binding proteins
MAPK: mitogen-activated protein kinase
MDHAR: monodehydroascorbate reductase
MS: mass spectrometry
MTT: 3-(4,5-dimethylthiazol-2-yl)-2,5-diphenyltetrazolium bromide
PEP: phosphoenolpyruvate
PFDb: prefoldin β subunit
PGM: phosphoglucomutase 1
PM: plasma membrane
POD: peroxidase
PPDK: pyruvate phosphate dikinase
PRP1: PRP1 splicing factor
PrxR: 2-Cys peroxiredoxin
PS: photosystem
PsbH: photosystem II protein H
RNA pol: RNA polymerases
ROS: reactive oxygen species
RP: reversed-phase
RPB8: RNA polymerase subunit 8
RPS3: ribosomal protein S3
RPS3a: ribosomal protein S3a
RPS6: ribosomal protein S6
S: serine
SCX: strong cation exchange chromatography
SnRK: Snf1-like protein kinase
SOD: superoxide dismutase
SRPPs: salinity-responsive phosphoproteins
SRPs: salinity-responsive proteins
T: threonine
TCP: T-complex protein 1 β subunit
UBC: ubiquitin-conjugating enzyme E2
RuBisCO: ribulose-1,5- isphosphate carboxylase/oxygenase
RPL12: ribosomal protein L12
Y: tyrosine.
