# Supplementary material for: Salinity-Induced Palmella Formation Mechanism in Halotolerant Algae Dunaliella salina Revealed by Quantitative Proteomics and Phosphoproteomics
Source: Front Plant Sci. 2017 May 23;8:810. doi: 10.3389/fpls.2017.00810 (PMC5441111; doi:10.3389/fpls.2017.00810)
Supplement: Supplementary file 4 [file Table4.DOC]

**Supplemental Table S4**

Summary of phosphoproteins and phosphorylation sites upon palmella formation of *D. salina*.

|  | **Unique Protein** | **Total**  **Sites** | **Phospho(STY) Numbers** | | | **Phospho(STY) Ratio** | | |
| --- | --- | --- | --- | --- | --- | --- | --- | --- |
|  |  |  | **S** | **T** | **Y** | **S** | **T** | **Y** |
| Identified sites | 100 | 137 | 78 | 52 | 7 | 0.57 | 0.38 | 0.05 |
| Quantified Sites | 40 | 54 | 33 | 19 | 2 | 0.61 | 0.35 | 0.04 |
| Increased Sites | 14 | 20 | 13 | 7 | 0 | 0.65 | 0.35 | 0 |
| Decreased Sites | 18 | 21 | 15 | 5 | 1 | 0.71 | 0.24 | 0.05 |
| Unchanged Sites | 10 | 12 | 4 | 7 | 1 | 0.34 | 0.58 | 0.08 |
| Uncertain Sites | 1 | 1 | 1 | 0 | 0 | 1.00 | 0 | 0 |

The numbers of phosphorylation sites and unique phosphoproteins identified/ quantified in *D. salina* upon palmella formation was shown in this table. 137 phosphorylation sites were identified, representing 100 phosphoproteins. Among them, 54 phosphorylation sites were quantified, representing 40 unique phosphoproteins. Finally, 20 salinity-increased phosphorylation sites were representing 15 phosphoproteins, 20 salinity-decreased phosphorylation sites were representing 17 phosphoproteins, 12 no-change phosphorylation sites were representing 10 phosphoproteins, and 2 uncertain phosphorylation sites were representing two phosphoproteins. The distribution of pS, pT and pY sites were also shown. All phosphorylation events having a reported localization probability of at least 0.75 were considered to be assigned to a specific residue, and we refer to these as phosphorylation sites, corresponding to unique phosphoproteins with FDR < 1% at peptide level. S, Serine, T, Threonine, Y, Tyrosine (details in Supplemental Table S3).
