# Supplementary material for: Salinity-Induced Palmella Formation Mechanism in Halotolerant Algae Dunaliella salina Revealed by Quantitative Proteomics and Phosphoproteomics
Source: Front Plant Sci. 2017 May 23;8:810. doi: 10.3389/fpls.2017.00810 (PMC5441111; doi:10.3389/fpls.2017.00810)
Supplement: Supplementary file 5 [file Table5.DOC]

**Supplemental Table S5**

Comparison of phosphoproteomic data in the three biological replicate experiments upon palmella formation of *D. salina*.

| **Quantitative information** | **Salinity shock-increased** | | |  | **Salinity shock-decreased** | | |  | **Salinity shock-responsive** | | |
| --- | --- | --- | --- | --- | --- | --- | --- | --- | --- | --- | --- |
|  | **Pro** | **Pep** | **Sites** |  | **Pro** | **Pep** | **Sites** |  | **Pro** | **Pep** | **Sites** |
| >＝1 | 14 | 16 | 21 |  | 22 | 29 | 25 |  | 35(1)^a^ | 45 | 46 |
| >＝2 | 6 | 7 | 8 |  | 9 | 12 | 6 |  | 15 | 19 | 14 |
| >＝3 | 1 | 1 | 1 |  | 1 | 2 | 1 |  | 2 | 3 | 2 |

The numbers of salinity shock-responsive phosphoproteins/phosphopeptides/phosphorylation sites with sample change trend in each biological replicate experiment were shown in this table. >=1, at least one biological replicate experiment; >=2, at least two biological experiments; >=3, both in three biological experiments. Pro, phosphoproteins; Pep, phosphopeptide; Sites, phosphorylation sites. ^a^, one phosphoprotein of 35 unique phosphoproteins is redundant (details in Supplemental Table S3).
