# Supplementary material for: Salinity-Induced Palmella Formation Mechanism in Halotolerant Algae Dunaliella salina Revealed by Quantitative Proteomics and Phosphoproteomics
Source: Front Plant Sci. 2017 May 23;8:810. doi: 10.3389/fpls.2017.00810 (PMC5441111; doi:10.3389/fpls.2017.00810)
Supplement: Supplementary file 10 [file Image1.PDF]

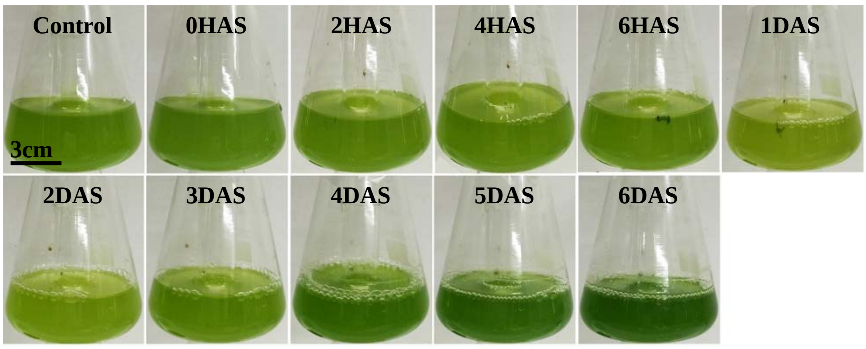

Supplemental Figure S1. Culture and salinity shock treatment of *Dunaliella salina*. The control sample of *D. salina* was cultivated in the medium containing 1 M NaCl under 8/16 h light/dark cycle with shaking at 100 rpm at 26 ºC. For salinity shock treatment, cells were transferred to medium containing 3 M NaCl from 0 hour after salinity shock (HAS) to 6 days after salinity shock (DAS).
