# Supplementary material for: Salinity-Induced Palmella Formation Mechanism in Halotolerant Algae Dunaliella salina Revealed by Quantitative Proteomics and Phosphoproteomics
Source: Front Plant Sci. 2017 May 23;8:810. doi: 10.3389/fpls.2017.00810 (PMC5441111; doi:10.3389/fpls.2017.00810)
Supplement: Supplementary file 11 [file Image2.PDF]

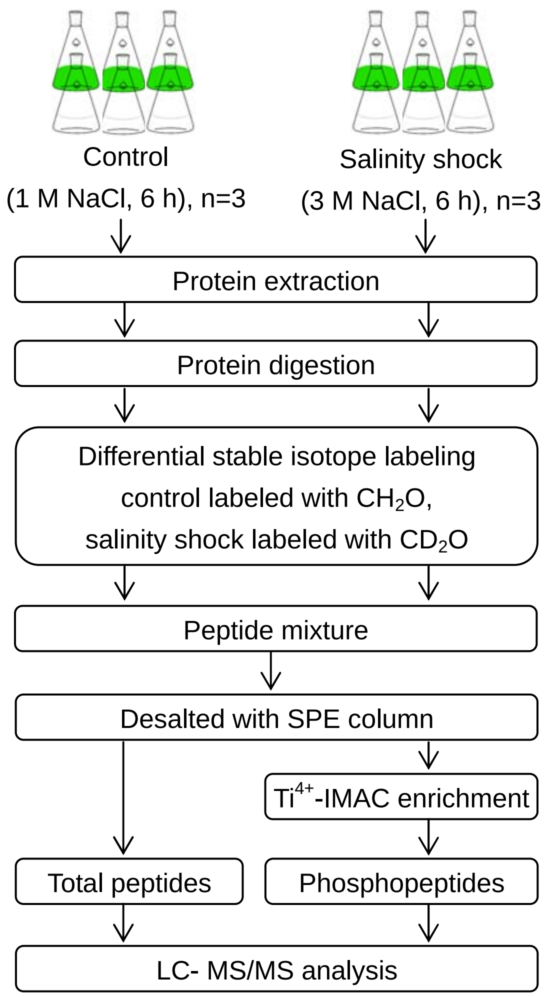


**Supplemental Figure S2.** Schematic overview of quantitative proteomic and phosphoproteomic approach for investigation of *Dunaliella salina* cells under control (1 M NaCl) and salinity shock (3 M NaCl for 6 h) conditions. The control sample of *D. salina* was cultivated in the medium containing 1 M NaCl under 8/16 h light/dark cycle with shaking at 100 rpm at 26 °C. For salinity shock treatment, cells were transferred to medium containing 3 M NaCl for 6 h. The extracted proteins were analyzed by in-solution digestion followed by differential stable isotope labeling and LC-MS/MS for quantitative proteomic analysis, by differential stable isotope labeling followed titanium immobilized metal ion affinity chromatography ($Ti^{4+}$-IMAC) phosphopeptides enrichment and LC-MS/MS for quantitative phosphoproteomic analysis. The methods in detail were described in Materials and Methods. All cultures were divided into three groups.
