# Supplementary material for: Salinity-Induced Palmella Formation Mechanism in Halotolerant Algae Dunaliella salina Revealed by Quantitative Proteomics and Phosphoproteomics
Source: Front Plant Sci. 2017 May 23;8:810. doi: 10.3389/fpls.2017.00810 (PMC5441111; doi:10.3389/fpls.2017.00810)

Supplemental Figure S3. MS/MS spectra of 35 salinity-responsive phosphoproteins upon palmella formation of *Dunaliella salina*. Ph indicates the phosphorylation site.

Protein:	gi|1279362|emb|CAA62413.1| Striated fiber-assemblin
Score:	132.26
Source:	phosphopeptide-24mM-2
Scan number:	14531

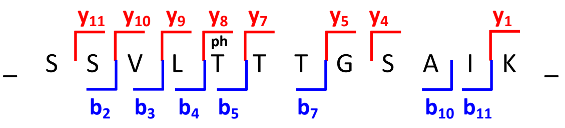

	207.15	306.33	419.41	502.40		704.74			919.72	1130.76	
	b_2	b_3	b_4	b_5*		b_7*			b_{10}*	b_{11}	
**S**	**S**	**V**	**L**	**T^{ph}**	**T**	**T**	**G**	**S**	**A**	**I**	**K**
	y_{11}	y_{10}	y_9	y_8	y_7		y_5	y_4			y_1
	1189.81	1102.85	1003.65	890.61	709.53		507.46	450.50			179.20

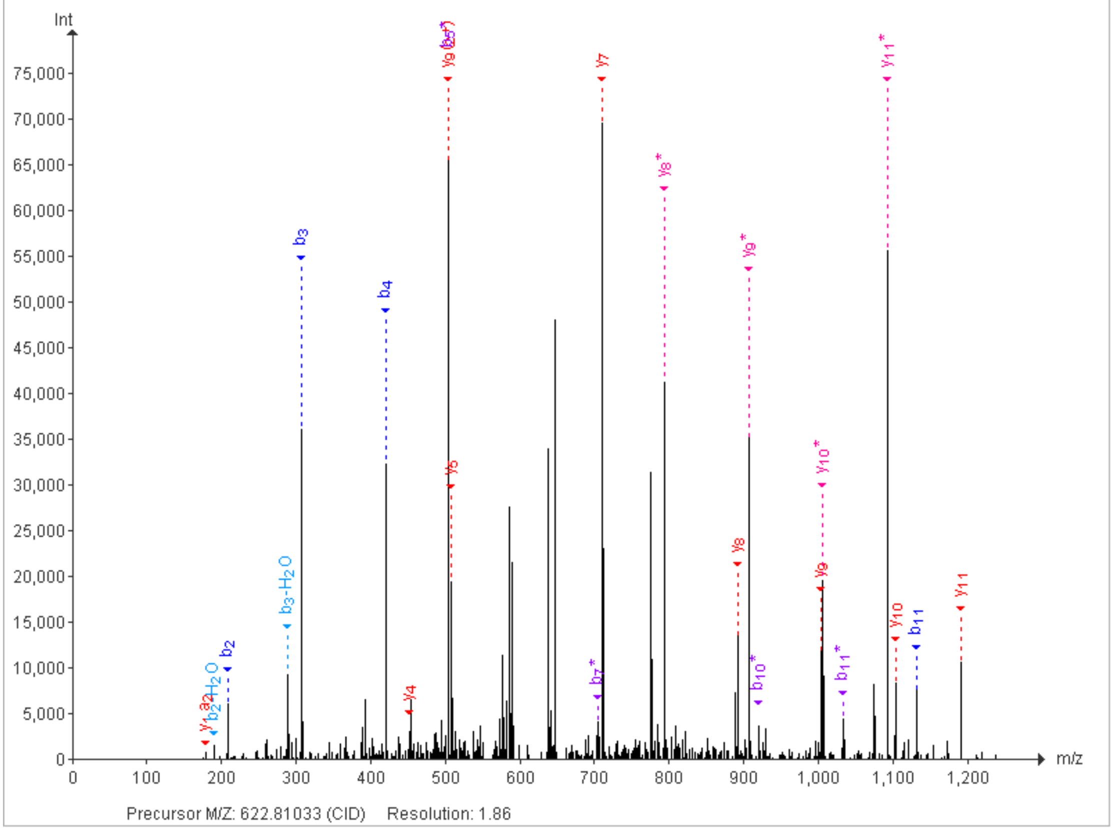

Protein: gi|1279362|emb|CAA62413.1| Striated fiber-assemblin
Score: 64.364
Source: phosphopeptide-0mM-b2
Scan number: 19864

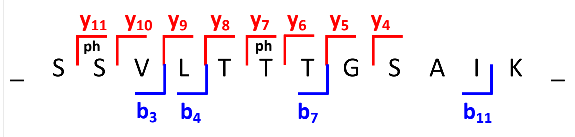

		386.41	499.37			686.52			919.72	1210.76	
		b_3	b_4			b_7*				b_{11}	
**S**	**S^{ph}**	**V**	**L**	**T^{ph}**	**T**	**T**	**G**	**S**	**A**	**I**	**K**
	y_{11}*	y_{10}	y_9	y_8	y_7^{2+}	y_6	y_5	y_4			
	1171.98	1102.70	1003.72	890.63	395.31	608.31	507.45	450.56			

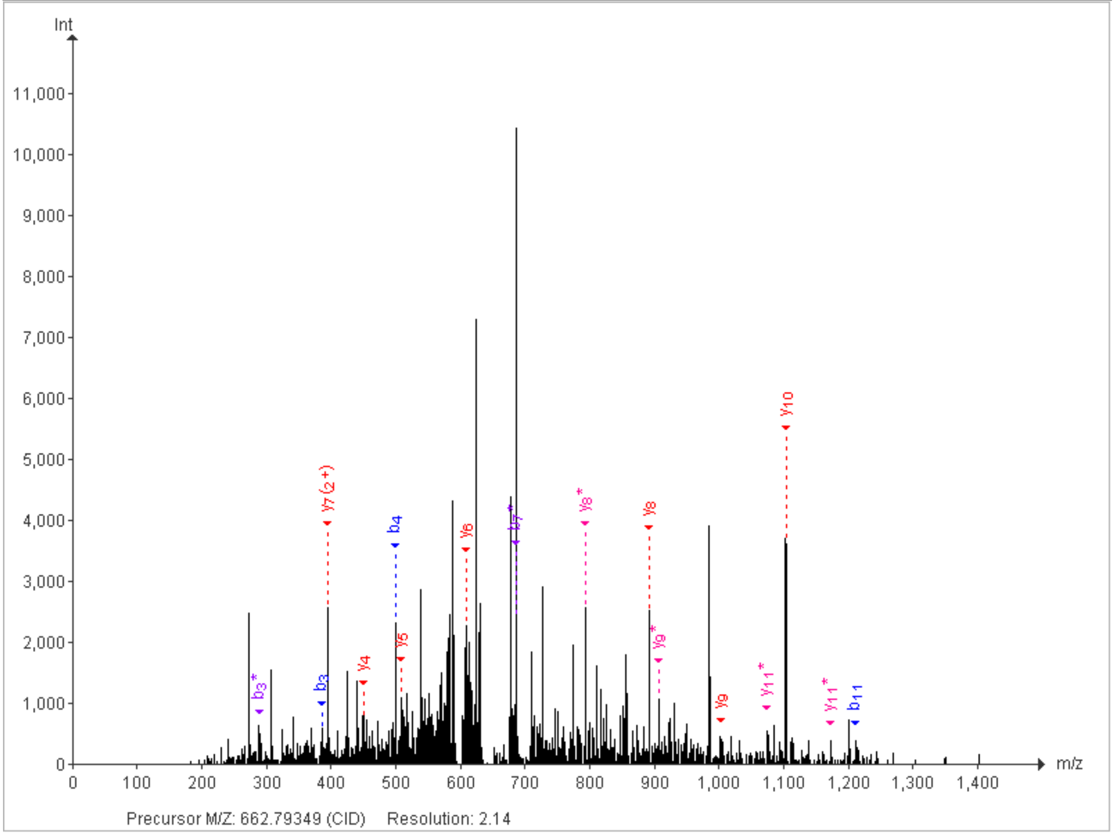

Protein:	gi|226524137|gb|ACO70118.1| flagellar associated protein
Score:	136.37
Source:	phosphopeptide-40mM-2
Scan number:	12607

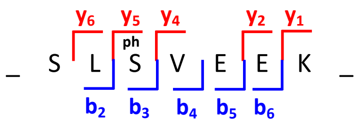

	229.34	396.29	397.58	624.37	753.46	686.52
	b_2	b_3	b_4*	b_5	b_6	b_7
**S**	**L**	**S^{ph}**	**V**	**E**	**E**	**K**
	y_6	y_5	y_4		y_2	y_1
	812.55	699.43	532.48		304.33	175.17

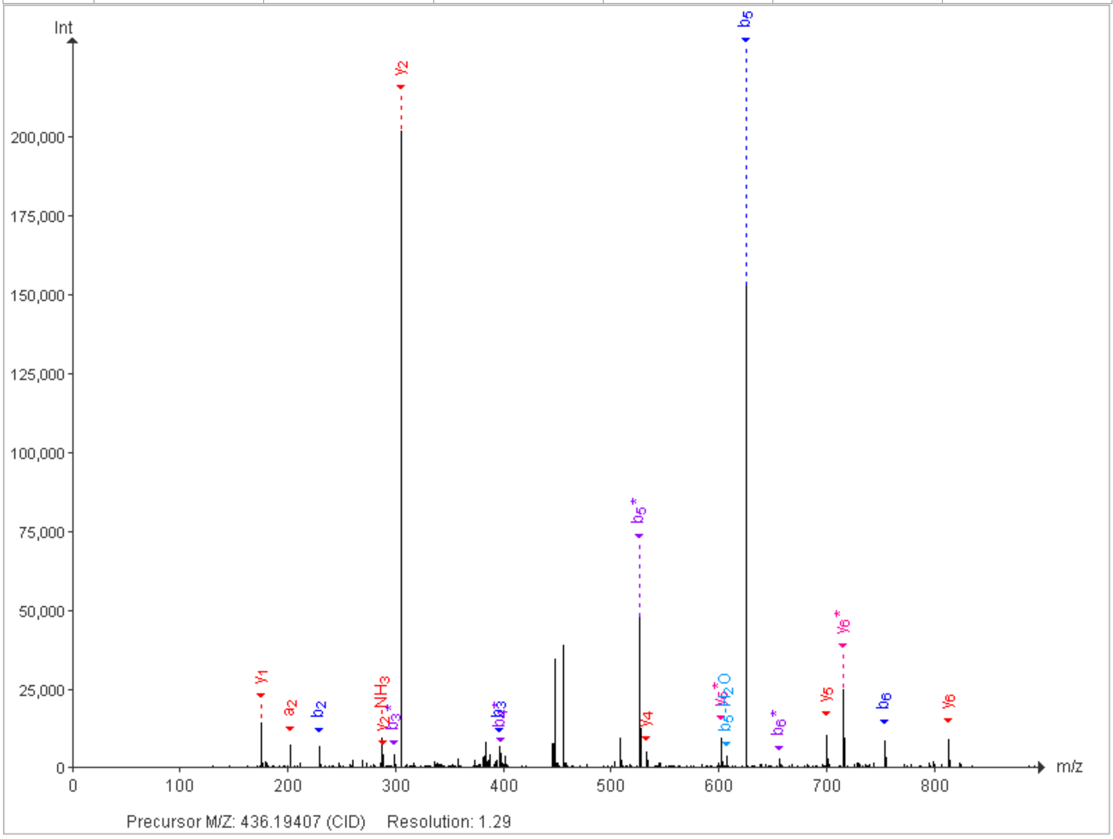

Protein: gi|2625154|gb|AAB86648.1| tubulin alpha chain

Score: 243.31

Source: phosphopeptide-56mM-2

Scan number: 26386

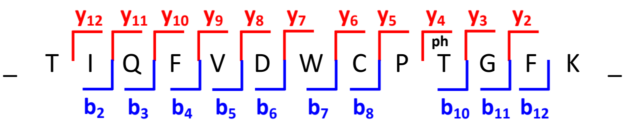

	243.32	371.35	518.32	617.45	732.50	918.57	1078.59		1258.68	1315.74	1560.77	
	b_2	b_3	b_4	b_5	b_6	b_7	b_8		b_{10}*	b_{11}*	b_{12}	
**T**	**I**	**Q**	**F**	**V**	**D**	**W**	**C**	**P**	**T^{ph}**	**G**	**F**	**K**
	y_{12}	y_{11}	y_{10}	y_9	y_8	y_7	y_6*	y_5	y_4	y_3	y_2	
	1605.86	1492.81	1364.67	1217.59	1118.57	1003.46	719.51	657.39	560.44	379.31	322.58	

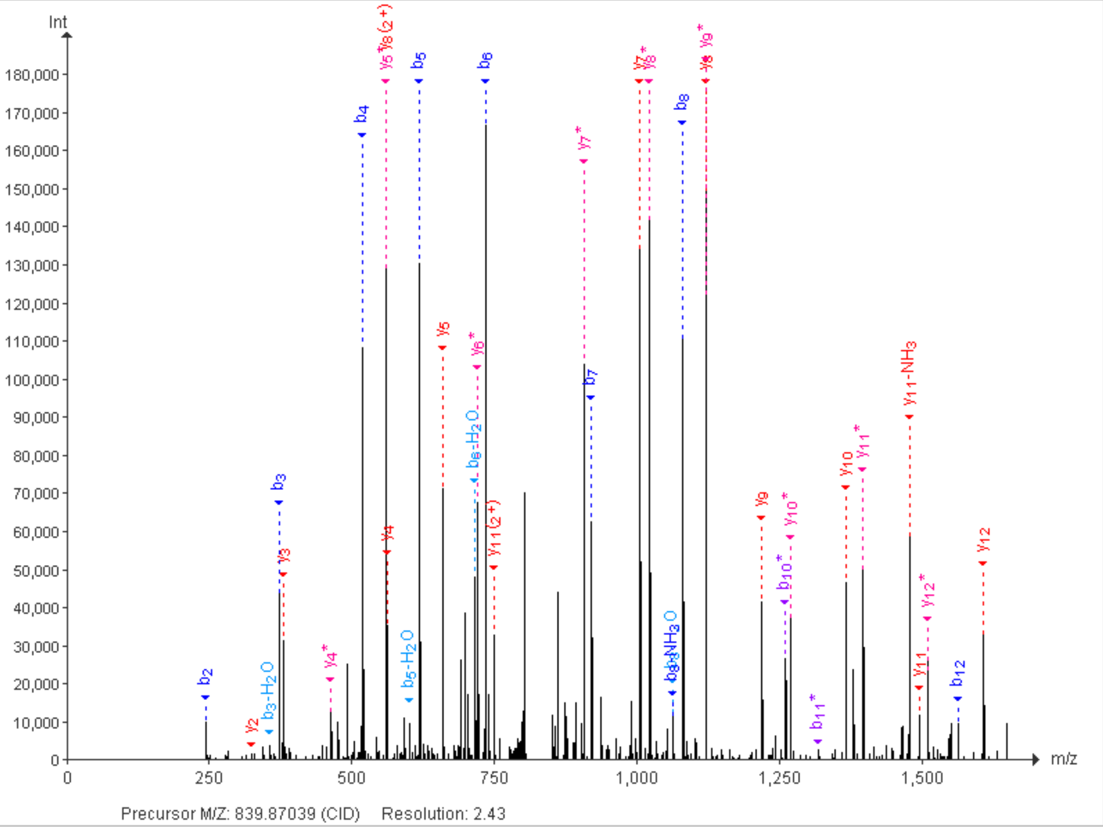

Protein:	gi|284518784|gb|ADB92502.1| kinesin-like calmodulin binding protein
Score:	207.39
Source:	phosphopeptide-24mM-B3
Scan number:	18942

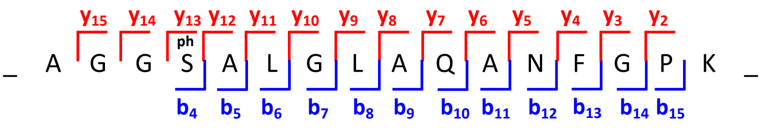

			381.45	452.28	565.33	622.33	637.46	708.51	934.54	1005.58	1119.55	1266.68	1323.75	1323.02	
			b_4	b_5	b_6	b_7	b_8*	b_9*	b_{10}	b_{11}	b_{12}	b_{13}	b_{14}	b_{15}*	
**A**	**G**	**G**	**S^{ph}**	**A**	**L**	**G**	**L**	**A**	**Q**	**A**	**N**	**F**	**G**	**P**	**K**
	y_{15}	y_{14}	y_{13}*	y_{12}	y_{11}	y_{10}	y_9	y_8	y_7	y_6	y_5	y_4	y_3	y_2	
	1495.91	1438.90	1283.87	1214.87	1143.79	1030.63	973.67	860.57	789.55	661.46	590.39	476.38	329.35	272.41	

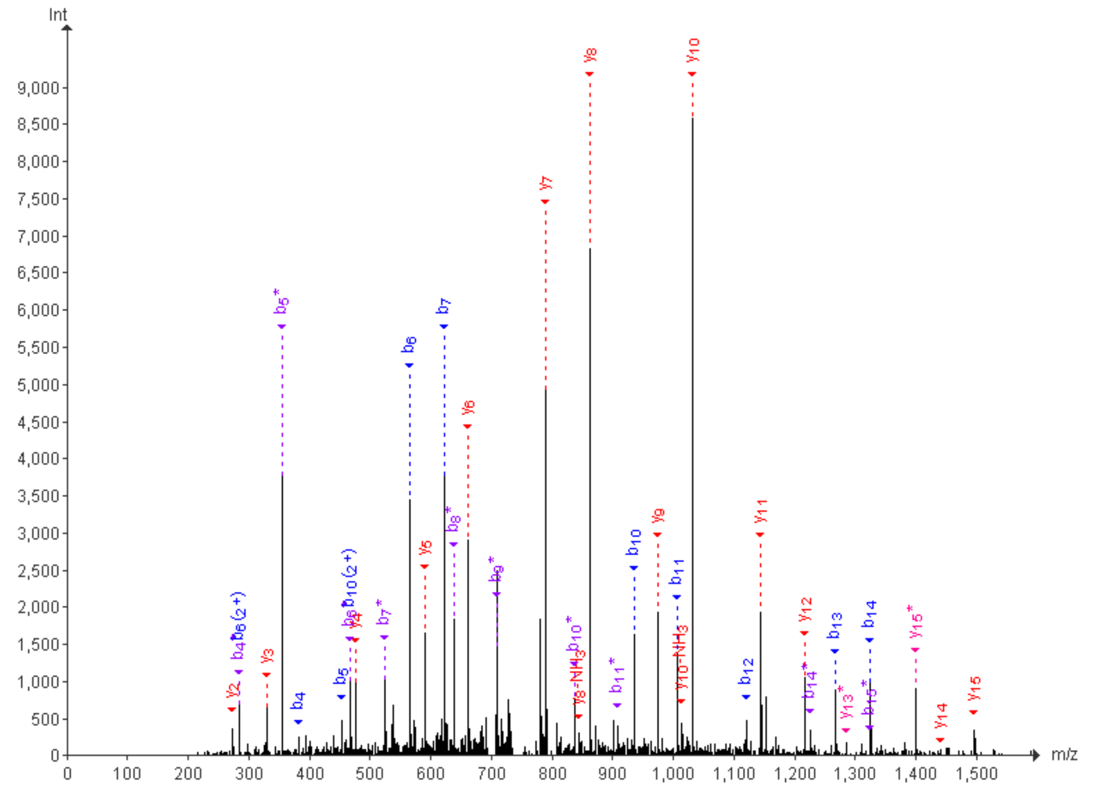

Precursor M/Z: 769.87178 (CID)   Resolution: 2.29

Protein:	gi|290465235|gb|ADD25038.1| phosphoglucomutase 1
Score:	151.98
Source:	phosphopeptide-1000mM-2
Scan number:	17948

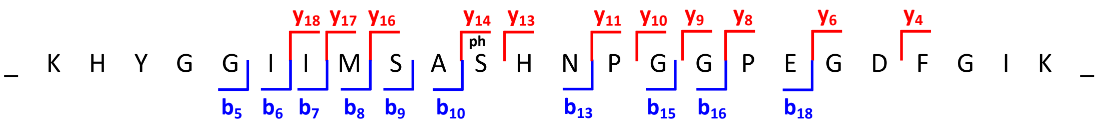

				599.46	712.66	825.71	956.70	1043.73	1114.75			1532.96		1588.92	1744.00		1872.15						
				b_5	b_6	b_7	b_8	b_9	b_{10}			b_{13}		b_{15}*	b_{16}		b_{18}*						
K	H	Y	G	G	I	I	M	S	A	S^{ph}	H	N	P	G	G	P	E	G	D	F	G	I	K
						y_{18}*	y_{17}*	y_{16}*		y_{14}*	y_{13}		y_{11}	y_{10}	y_9	y_8		y_6		y_4			
						1824.23	1710.92	1579.90		1421.84	1352.86		1101.67	1004.58	947.69	890.62		664.44		492.37			

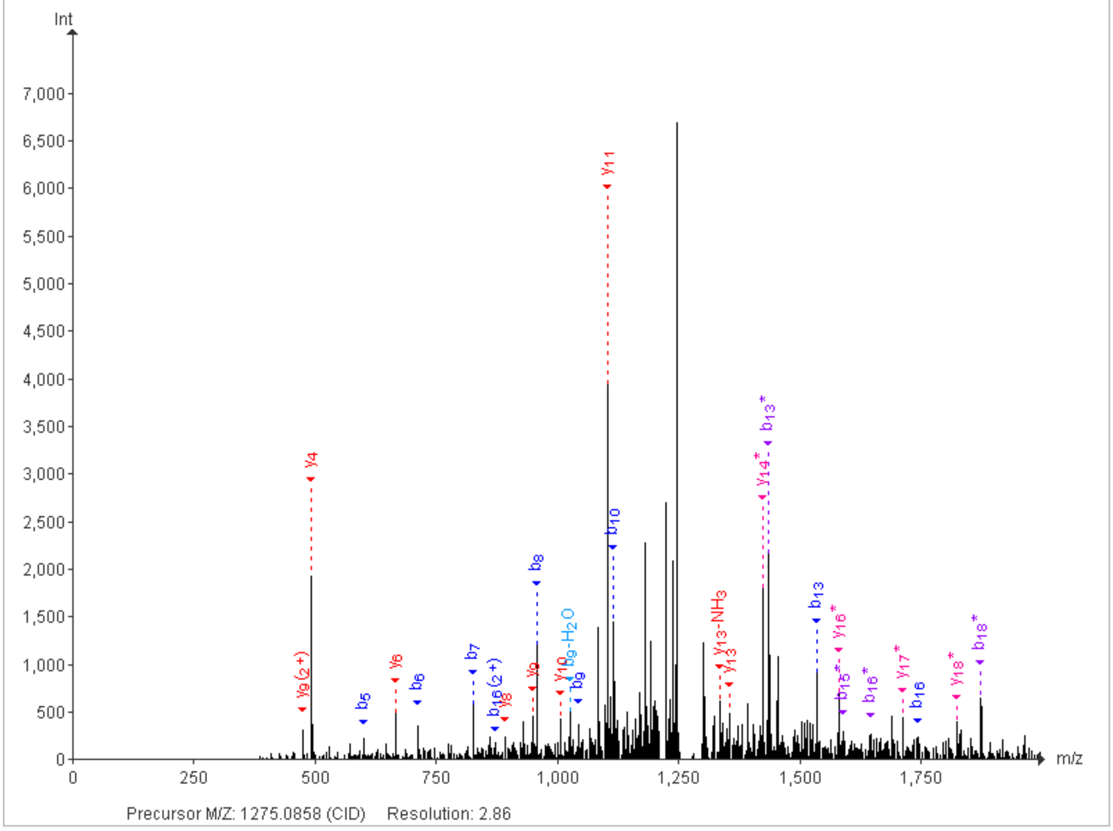

Protein:	gi|290465235|gb|ADD25038.1| phosphoglucomutase 1
Score:	93.669
Source:	phosphopeptide-1000mM-B2
Scan number:	21964

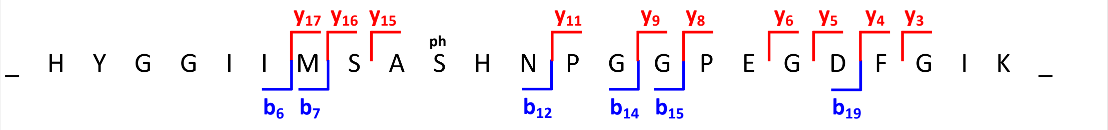

					669.56	400.97					689.11		766.17	794.71				993.52				
					b_6	b_7^{2+}					b_{12}^{2+}		b_{14}^{2+}	b_{15}^{2+}				b_{19}^{2+}				
**H**	**Y**	**G**	**G**	**I**	**I**	**M**	**S**	**A**	**S^{ph}**	**H**	**N**	**P**	**G**	**G**	**P**	**E**	**G**	**D**	**F**	**G**	**I**	**K**
						y_{17}^{2+}	y_{16}^{2+}	y_{15}^{2+}				y_{11}		y_9	y_8		y_6	y_5	y_4	y_3		
						905.24	839.60	795.52				1101.84		947.76	890.66		664.49	607.50	492.27	345.56		

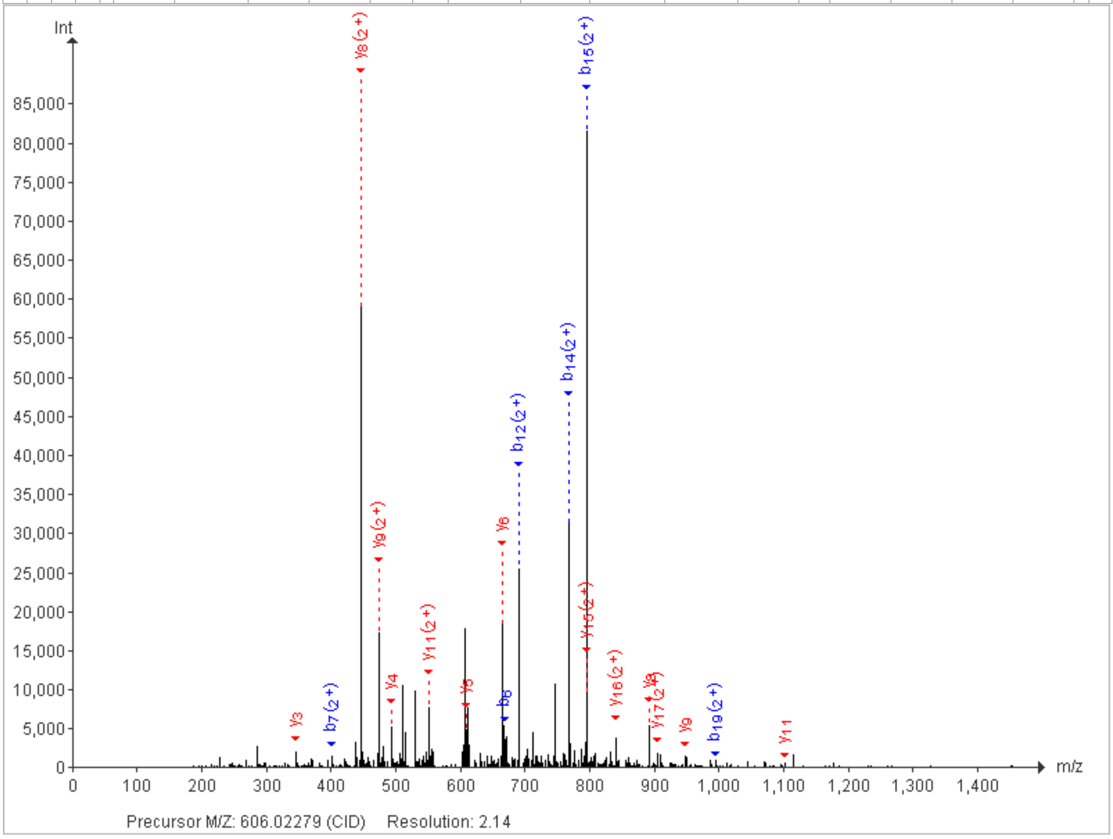

Protein: gi|208463466|gb|ACI29026.1| glucose-6-phosphate isomerase
Score: 123.11
Source: phosphopeptide-56mM-2
Scan number: 22878

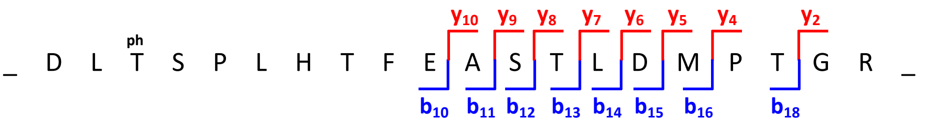

									1249.74	1320.71	1407.80	754.56	1621.94	1736.94	942.70		1041.55		
									b_{10}	b_{11}	b_{12}	b_{13}^{2+}	b_{14}	b_{15}	b_{16}^{2+}		b_{18}^{2+}		
**D**	**L**	**T^{ph}**	**S**	**P**	**L**	**H**	**T**	**F**	**E**	**A**	**S**	**T**	**L**	**D**	**M**	**P**	**T**	**G**	**R**
										y_{10}	y_9	y_8	y_7	y_6	y_5	y_4		y_2	
										1064.55	993.60	906.62	805.52	692.34	577.38	430.39		232.31	

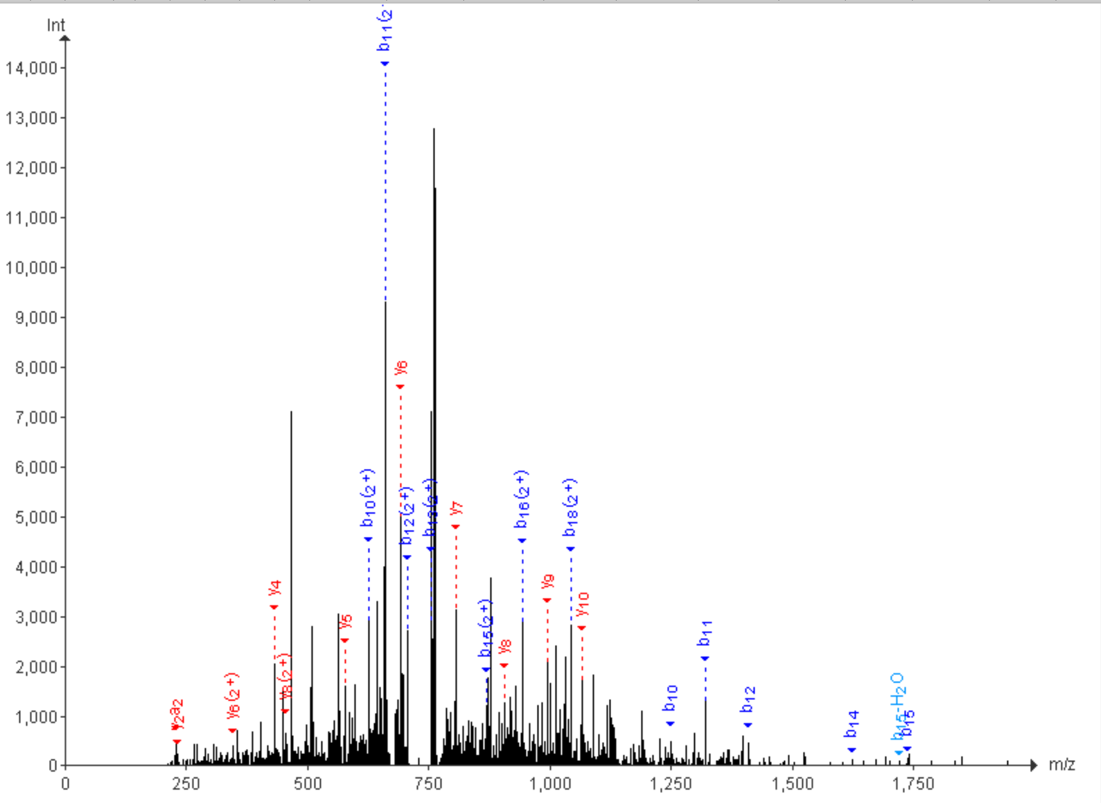

Precursor M/Z: 762.34337 (CID) Resolution: 2.86

Protein:	gi|300259927|gb|EFJ44150.1| hypothetical protein VOLCADRAFT_65163, containing pfam01238 phosphomannose isomerase type I domain*
Score:	132.19
Source:	phosphopeptide-200mM-B2
Scan number:	13463

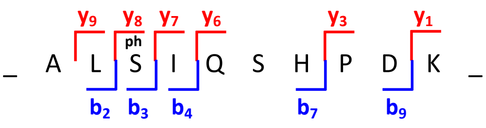

	213.28	282.36	395.46			845.49		1057.63	502.40
	b_2	b_3*	b_4*			b_7*		b_9	
**A**	**L**	**S^{ph}**	**I**	**Q**	**S**	**H**	**P**	**D**	**K**
	y_9	y_8*	y_7	y_6			y_3		y_1
	1132.78	921.66	852.69	739.52			387.43		175.22

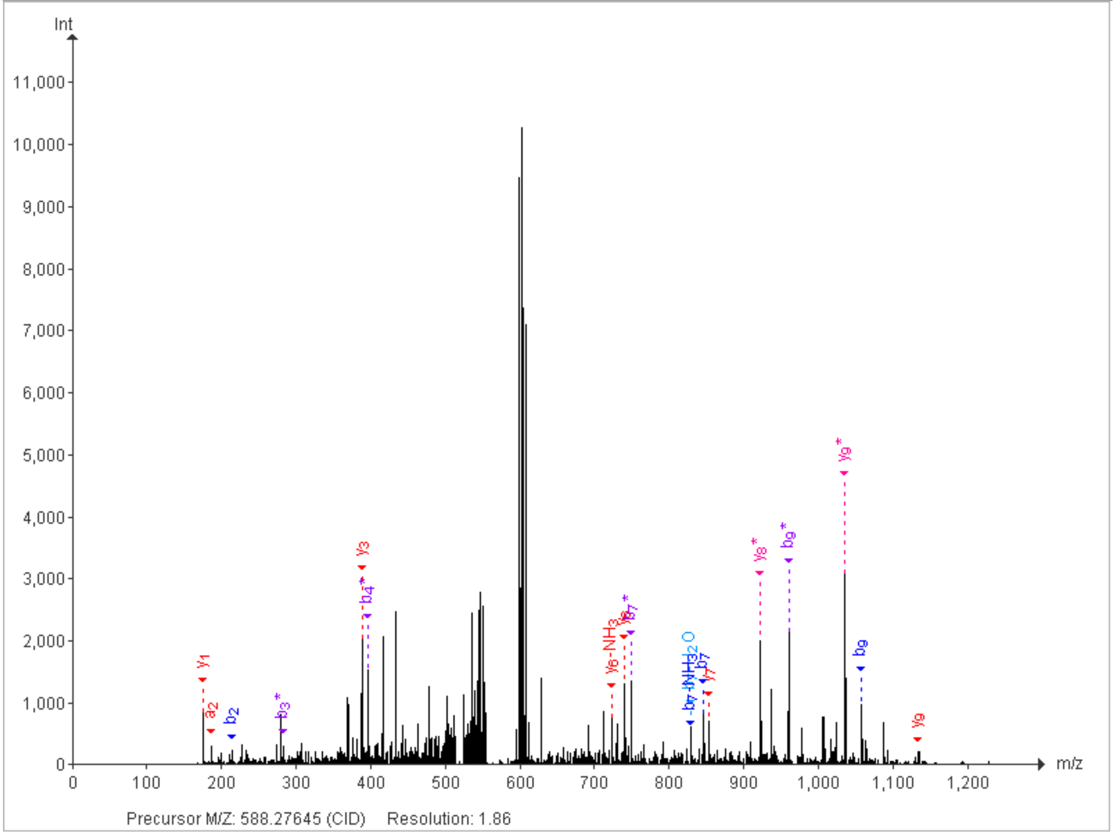

Protein:	gi|333691283|gb|AEF79974.1| chloroplast minor chlorophyll a-b binding protein of photosystem II
Score:	174.36
Source:	phosphopeptide-40mM-2
Scan number:	15266

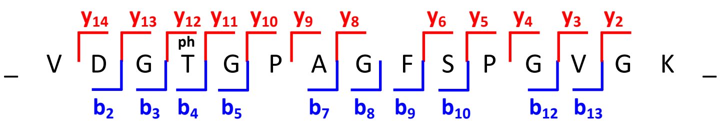

	247.37	304.40	485.24	542.36		612.54	669.51	914.60	1001.62		1155.79	1156.70		
	b_2	b_3	b_4	b_5		b_7	b_8*	b_9	b_{10}		b_{12}	b_{13}*		
**V**	**D**	**G**	**T**^{ph}	**G**	**P**	**A**	**G**	**F**	**S**	**P**	**G**	**V**	**G**	**K**
	y_{14}	y_{13}	y_{12}	y_{11}	y_{10}	y_9	y_8		y_6	y_5	y_4	y_3	y_2	
	1358.79	1243.79	1186.84	1005.73	948.67	851.60	780.64		576.49	489.49	392.46	335.58	236.31	

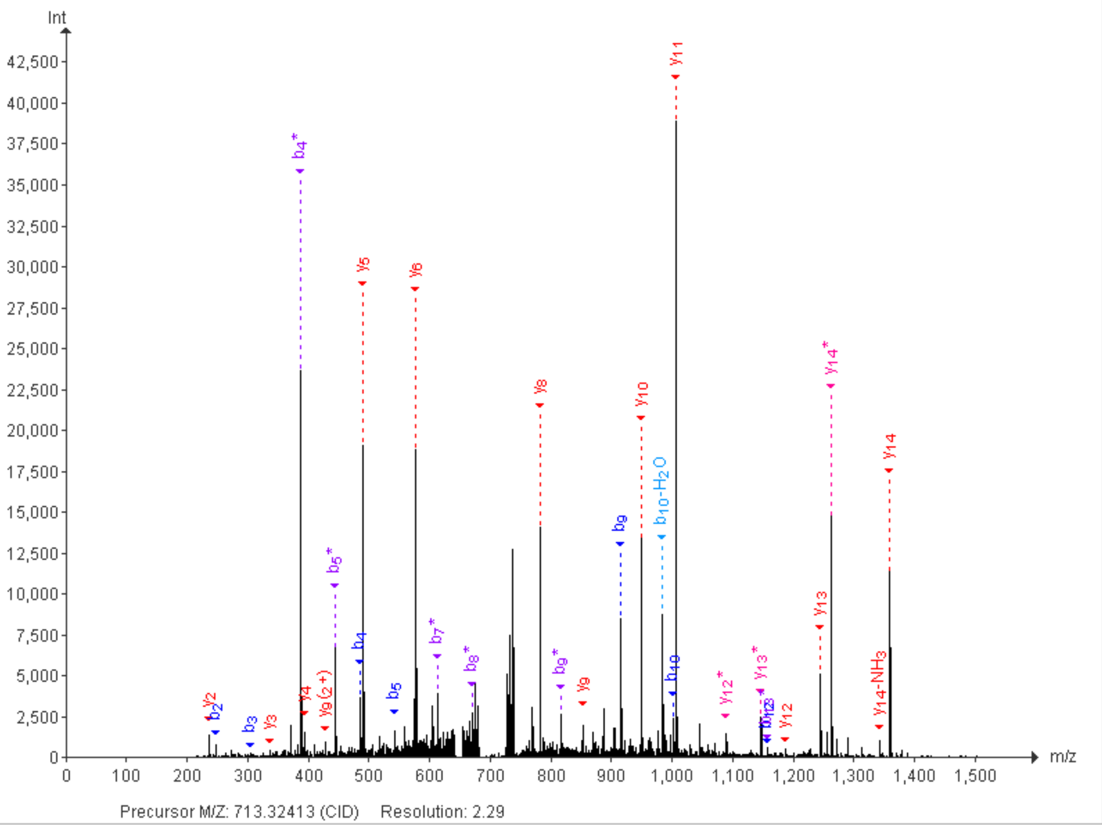

Protein:	gi|333691283|gb|AEF79974.1| chloroplast minor chlorophyll a-b binding protein of photosystem II
Score:	121.94
Source:	phosphopeptide-200mM-B2
Scan number:	21593

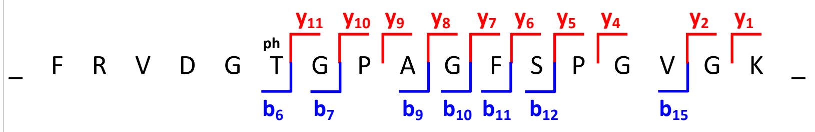

					788.45	845.51		1013.58	1070.57	1217.74	1304.77			779.59		
					b_6	b_7		b_9	b_{10}	b_{11}	b_{12}			b_{15}^{2+}		
**F**	**R**	**V**	**D**	**G**	**T^{ph}**	**G**	**P**	**A**	**G**	**F**	**S**	**P**	**G**	**V**	**G**	**K**
						y_{11}	y_{10}	y_9	y_8	y_7	y_6	y_5	y_4		y_2	y_1
						1005.63	948.65	851.57	780.58	723.53	576.44	489.45	392.44		236.28	179.24

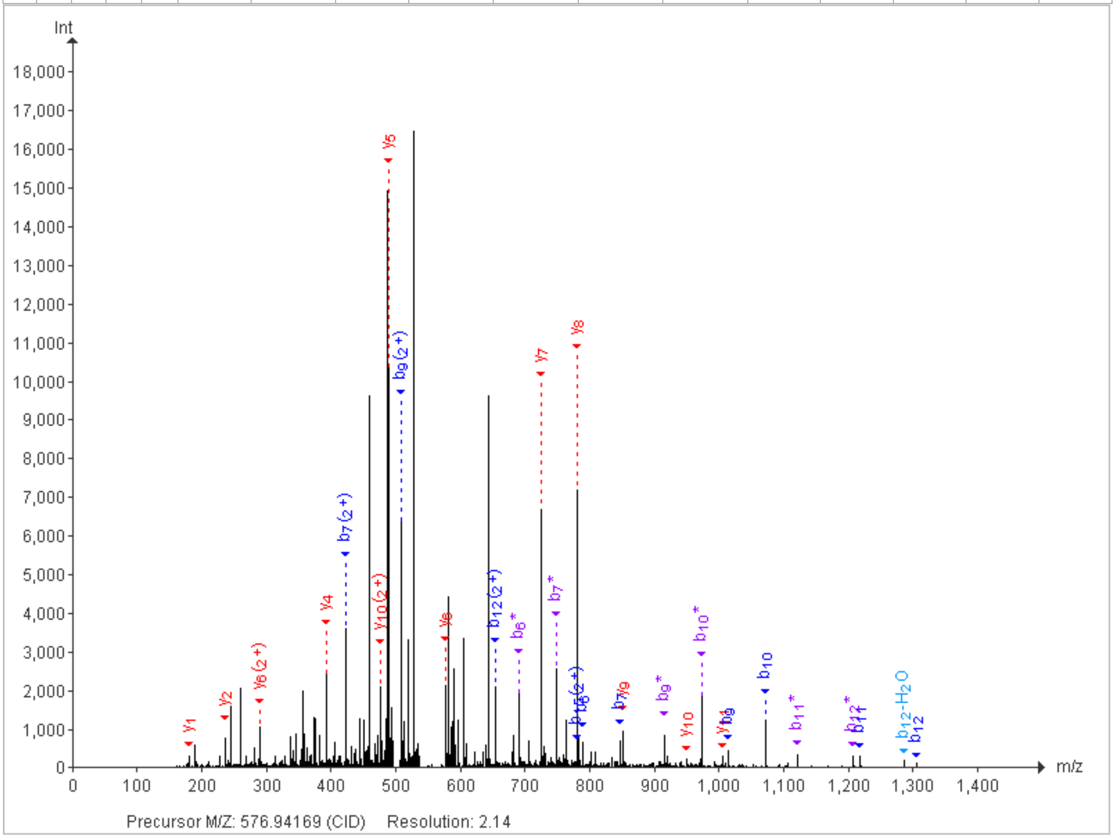

Protein:	gi|246880776|gb|ACS95088.1| photosystem II protein H
Score:	143.91
Source:	phosphopeptide-40mM-2
Scan number:	28738

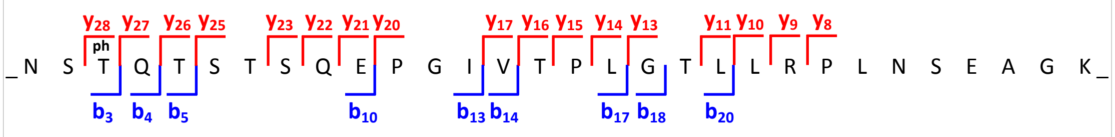

		411.21	539.04	640.32					1172.61			1439.60	1538.85			1850.12	1906.50		1060.99										
		b_3	b_4	b_5					b_{10}			b_{13}	b_{14}			b_{17}	b_{18}		b_{20}^{2+}										
**N**	**S**	**T^{ph}**	**Q**	**T**	**S**	**T**	**S**	**Q**	**E**	**P**	**G**	**I**	**V**	**T**	**P**	**L**	**G**	**T**	**L**	**L**	**R**	**P**	**L**	**N**	**S**	**E**	**A**	**G**	**K**
		y_{28}^{2+}	y_{27}^{2+}	y_{26}^{2+}	y_{25}^{2+}		y_{23}^{2+}	y_{22}^{2+}	y_{21}^{2+}	y_{20}^{2+}			y_{17}	y_{16}	y_{15}	y_{14}^{2+}	y_{13}^{2+}		y_{11}^{2+}	y_{10}^{2+}	y_9	y_8							
		1502.24	1412.11	1348.16	1297.68		1203.49	1160.05	1095.89	1031.35			1794.45	1695.35	1594.09	749.19	692.61		613.55	557.09	999.10	843.60							

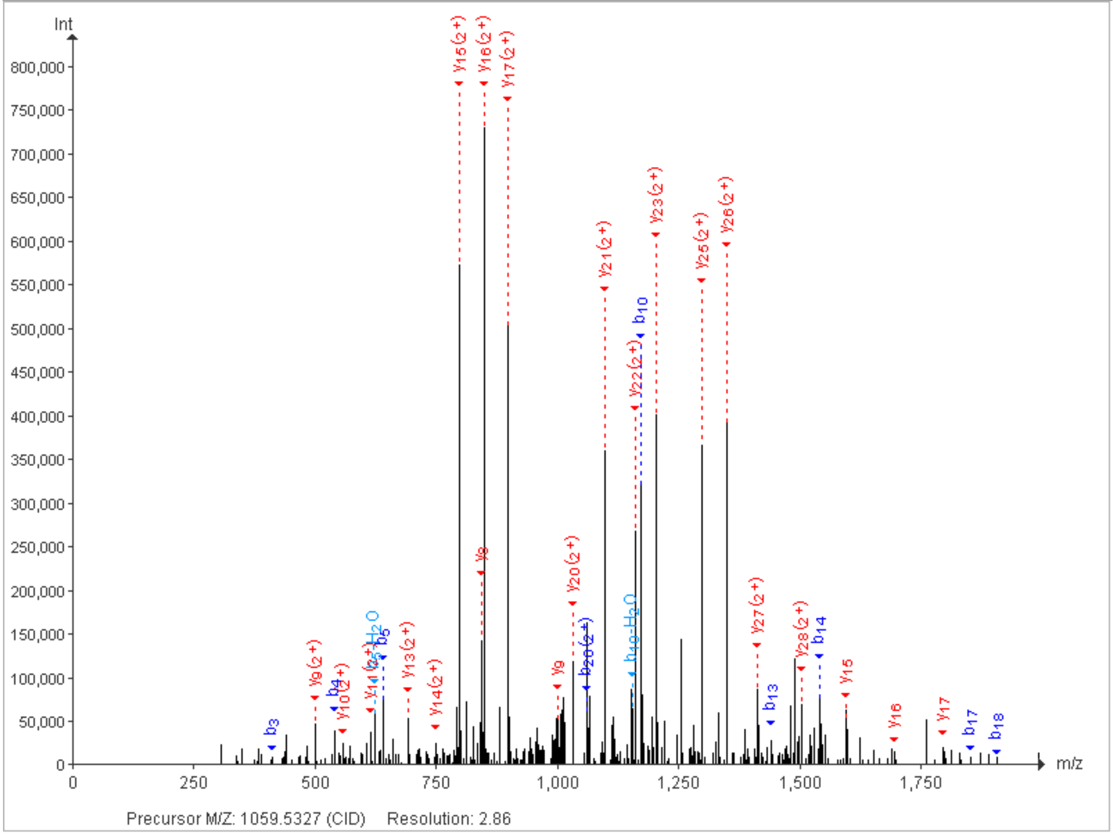

Protein:	gi|158277740|gb|EDP03507.1| pyruvate phosphate dikinase
Score:	186.12
Source:	phosphopeptide-100mM-B2
Scan number:	24555

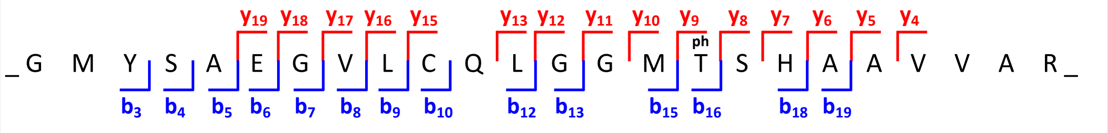

		396.38	483.16	554.31	683.37	740.47	839.55	952.50	1112.68		1353.70	1410.91		1598.87	1681.93		1906.00	1976.97					
		b_3	b_4	b_5	b_6	b_7	b_8	b_9	b_{10}		b_{12}	b_{13}		b_{15}	b_{16}*		b_{18}*	b_{19}*					
**G**	**M**	**Y**	**S**	**A**	**E**	**G**	**V**	**L**	**C**	**Q**	**L**	**G**	**G**	**M**	**T^{ph}**	**S**	**H**	**A**	**A**	**V**	**V**	**A**	**R**
					y_{19}*	y_{18}	y_{17}*	y_{16}	y_{15}*		y_{13}	y_{12}*	y_{11}*	y_{10}*	y_9	y_8	y_7	y_6	y_5	y_4			
					1938.10	1907.14	1751.98	1751.00	1539.91		1349.92	1138.74	1081.76	1024.73	991.60	810.64	723.56	586.50	515.41	444.44			

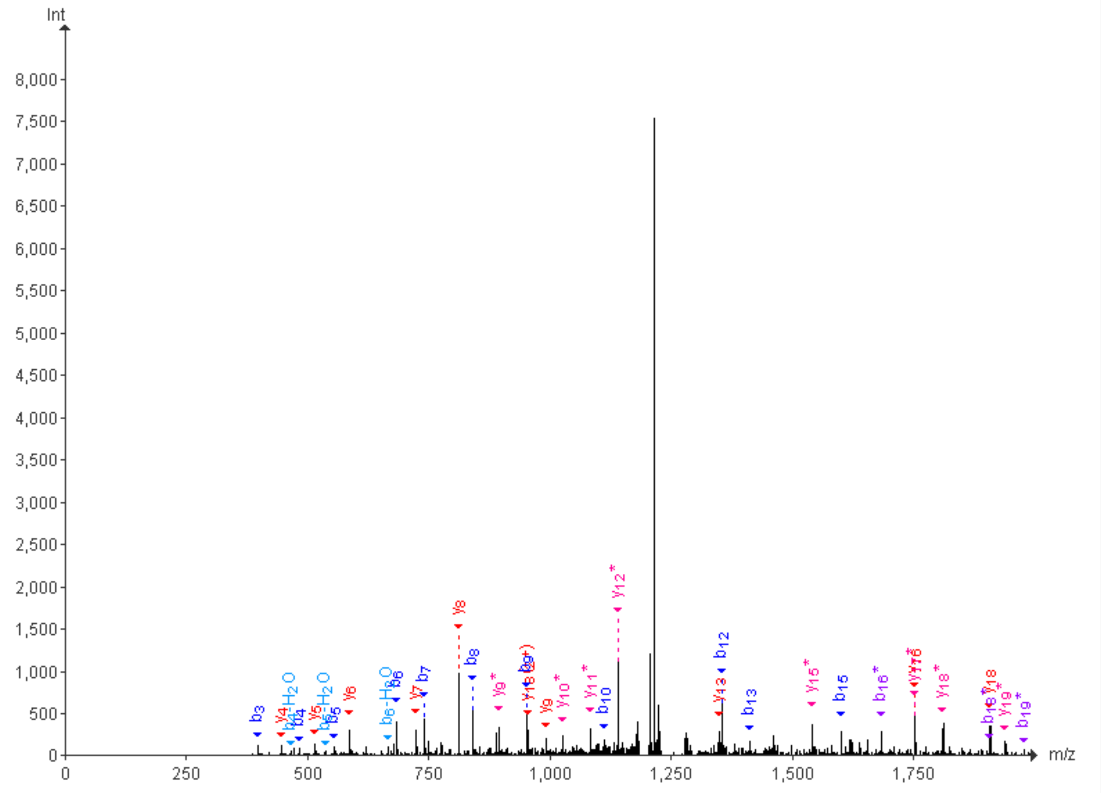

Protein: gi|1173346|sp|P46284.1| sedoheptulose-1,7-bisphosphatase
Score: 83.115
Source: phosphopeptide-40mM-B2
Scan number: 21786

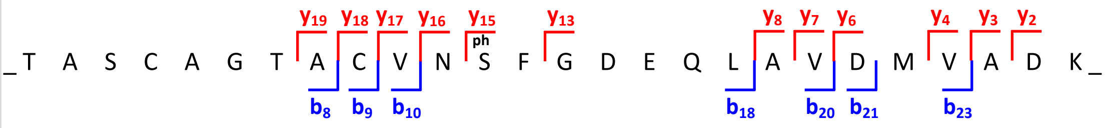

							374.51	455.01	1007.87								989.60		1074.71	1132.40		1254.80			
							b_8^{2+}	b_9^{2+}	b_{10}								b_{18}^{2+}		b_{20}^{2+}	b_{21}^{2+}		b_{23}^{2+}			
T	A	S	C	A	G	T	A	C	V	N	S^{ph}	F	G	D	E	Q	L	A	V	D	M	V	A	D	K
							y_{19}^{2+}	y_{18}^{2+}	y_{17}^{2+}	y_{16}^{2+}	y_{15}^{2+}		y_{13}					y_8	y_7	y_6^{2+}		y_4	y_3	y_2	
							1097.37	1061.80	981.72	932.27	874.96		1434.94					892.67	821.56	361.42		460.26	361.42	290.32	

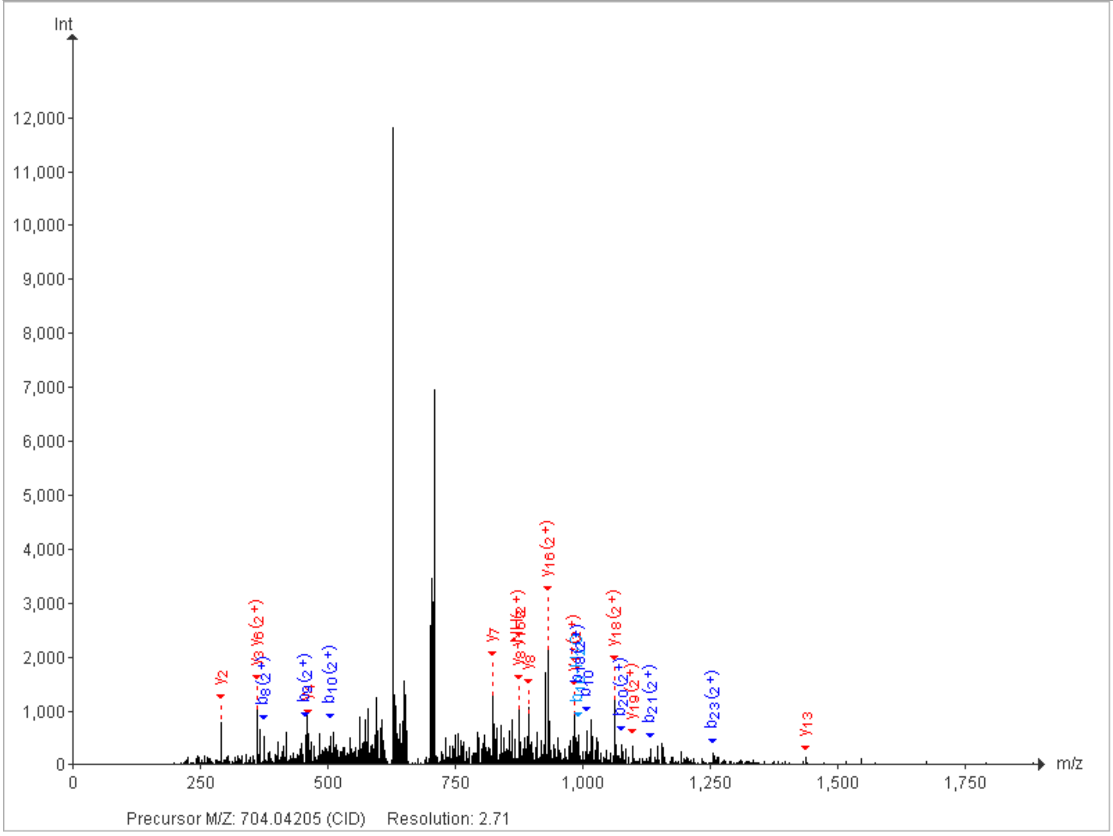

Protein: gi|1173346|sp|P46284.1| sedoheptulose-1,7-bisphosphatase
Score: 103.59
Source: phosphopeptide-72mM-B3
Scan number: 22486

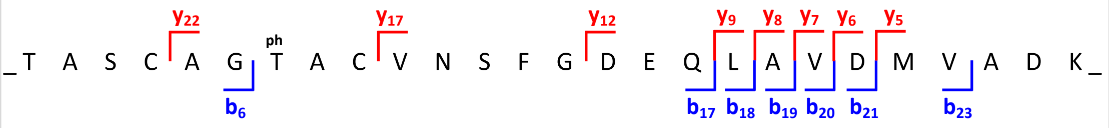

					576.60											1864.99	1978.07	1025.39	1074.92	1131.96		1247.41			
					b_6											b_{17}	b_{18}	b_{19}^{2+}	b_{20}^{2+}	b_{21}^{2+}		b_{23}^{2+}			
T	A	S	C	A	G	T[ph]	A	C	V	N	S	F	G	D	E	Q	L	A	V	D	M	V	A	D	K
				y_{22}^{2+}					y_{17}					y_{12}			y_9	y_8	y_7	y_6	y_5				
				1204.01					1866.13					1362.06			989.80	876.71	805.50	706.53	591.46				

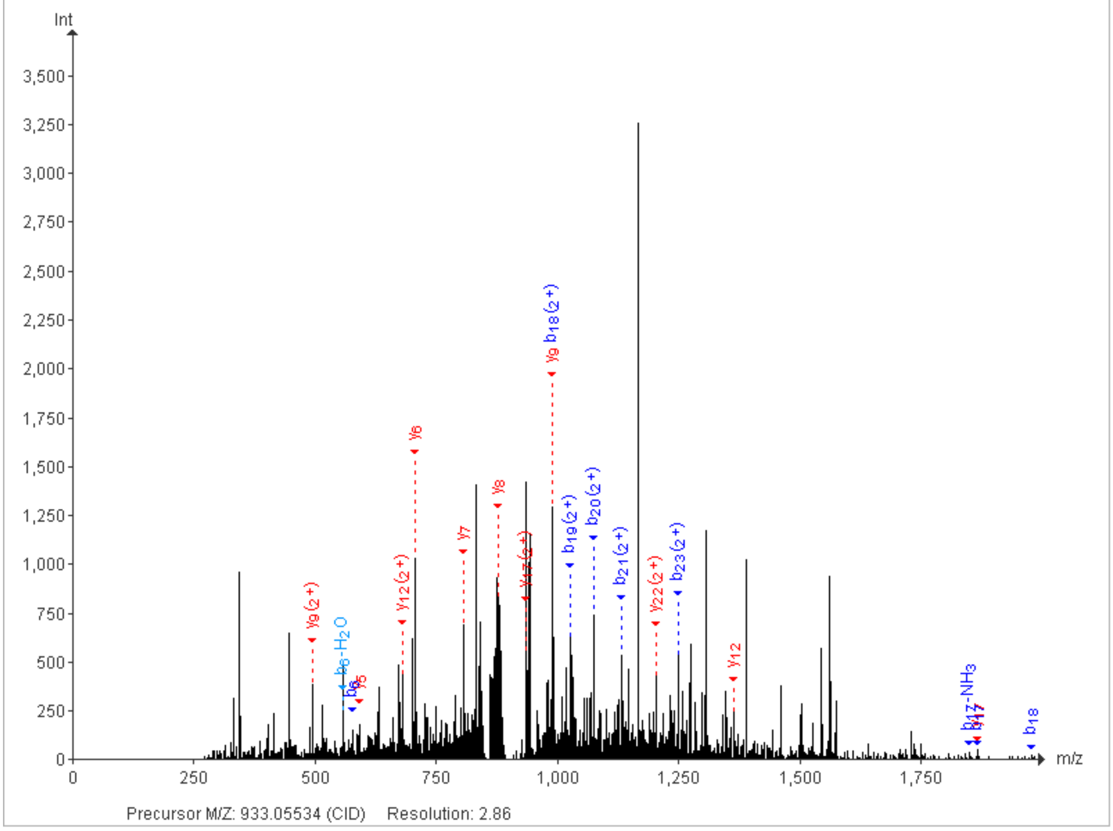

Protein:	gi|246880744|gb|ACS95056.1| ATP synthase CF1 alpha subunit
Score:	44.055
Source:	phosphopeptide-40mM-B2
Scan number:	13706

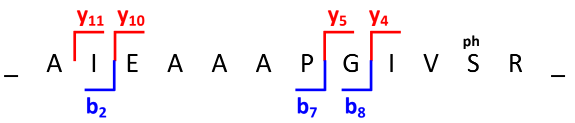

	217.24					656.47	357.36				
	b_2					b_7	b_8^{2+}				
**A**	**I**	**E**	**A**	**A**	**A**	**P**	**G**	**I**	**V**	**S^{ph}**	**R**
	y_{11}	y_{10}					y_5*	y_4			
	1163.75	1050.60					513.39	554.08			

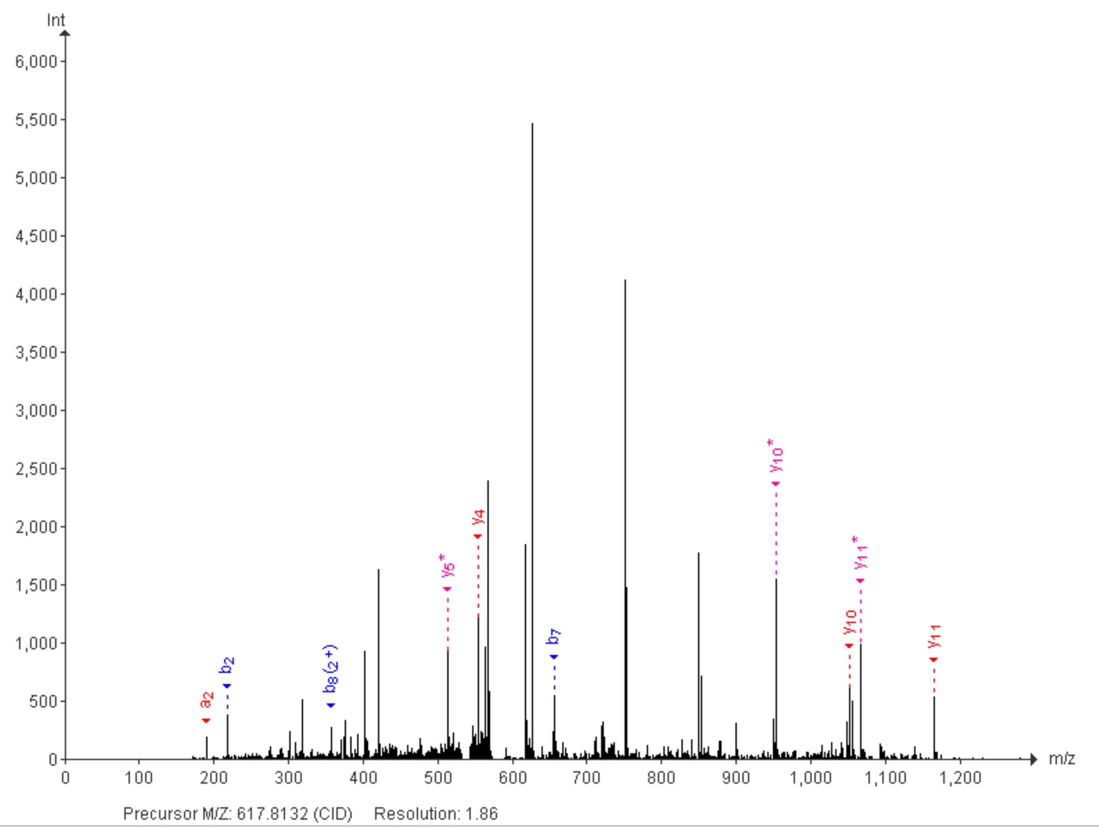

Protein:	gi|307106155|gb|EFN54402.1| hypothetical protein CHLNCDRAFT_48950, containing cd00831 chalcone and stilbene synthases domain*
Score:	110.32
Source:	phosphopeptide-56mM-2
Scan number:	13624

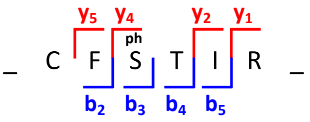

	336.19	503.26	506.29	717.35	
	b_2	b_3	b_4*	b_5	
**C**	**F**	**S^{ph}**	**T**	**I**	**R**
	y_5	y_4		y_2	y_1
	703.42	556.33		288.34	175.19

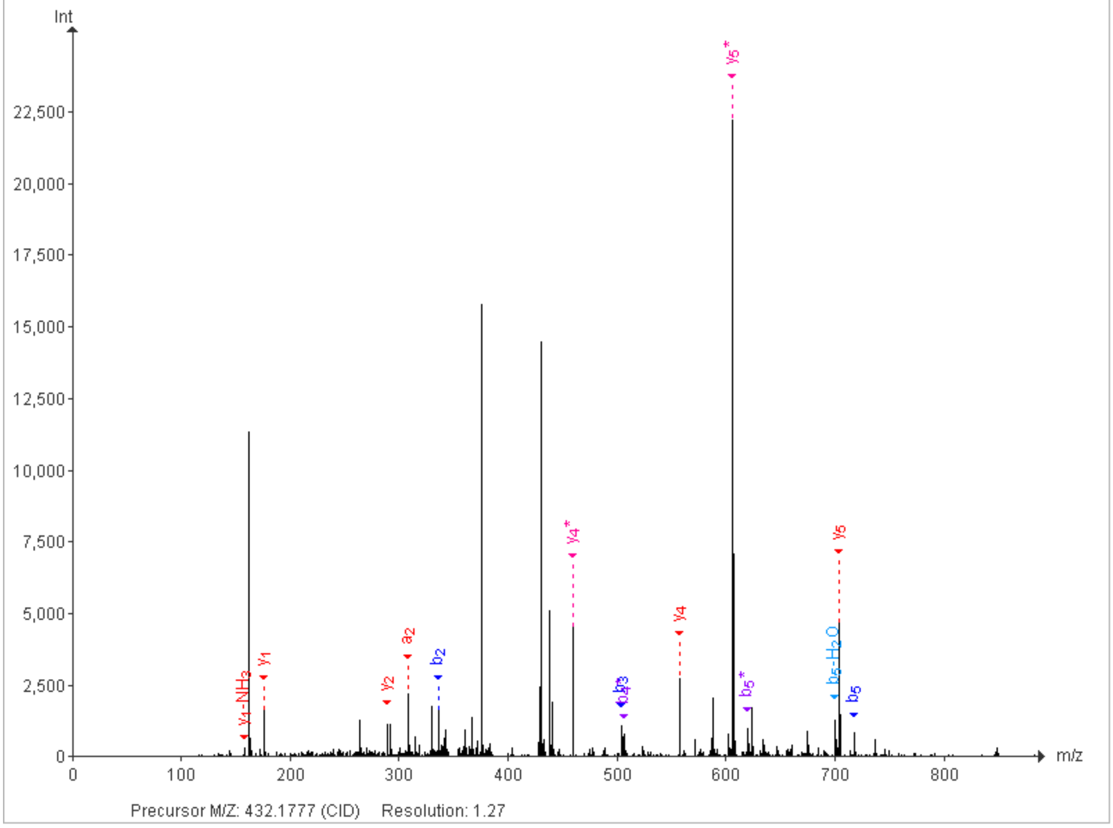

Protein:	gi|227477517|emb|CAY33684.1| unnamed protein product, containing pfam00120 glutamine synthetase domain*
Score:	109.16
Source:	phosphopeptide-1000mM-2
Scan number:	28595

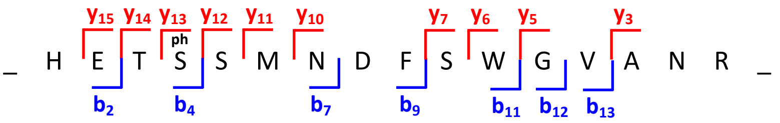

	295.32		563.32			895.50		1157.43		1332.34	1389.09	1586.72			
	b_2		b_4			b_7		b_9		b_{11}*	b_{12}*	b_{13}			
**H**	**E**	**T**	**S^{ph}**	**S**	**M**	**N**	**D**	**F**	**S**	**W**	**G**	**V**	**A**	**N**	**R**
	y_{15}*	y_{14}*	y_{13}*	y_{12}	y_{11}	y_{10}			y_7	y_6	y_5		y_3		
	1682.89	1553.72	1452.95	1383.74	1296.78	1165.59			789.53	702.50	516.49		360.44		

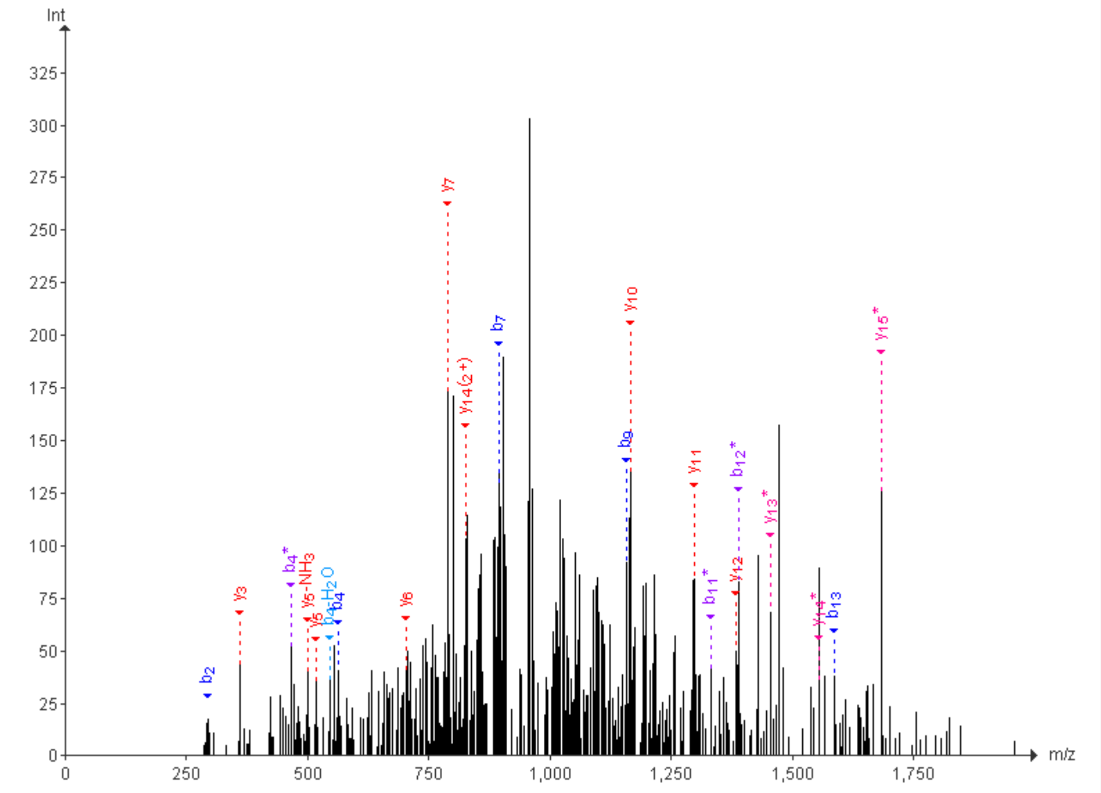

Precursor M/Z: 959.38291 (CID)   Resolution: 2.86

Protein: gi|283139174|gb|ADB12592.1| glutamine synthetase II

Score: 223.1

Source: phosphopeptide-200mM-2

Scan number: 16580

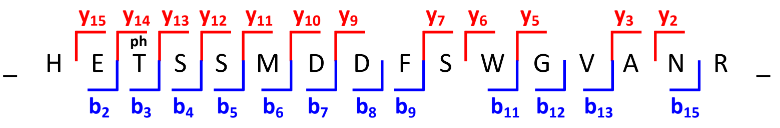

	295.30	476.24	465.39	552.29	781.43	896.41	1011.44	1158.48		1333.69	1390.76	1587.81		1772.71	
	b_2	b_3	b_4*	b_5*	b_6	b_7	b_8	b_9		b_{11}	b_{12}*	b_{13}		b_{15}	
**H**	**E**	**T^{ph}**	**S**	**S**	**M**	**D**	**D**	**F**	**S**	**W**	**G**	**V**	**A**	**N**	**R**
	y_{15}	y_{14}	y_{13}	y_{12}	y_{11}	y_{10}	y_9		y_7	y_6	y_5		y_3	y_2	
	1781.93	1652.85	1471.81	1384.70	1297.70	1166.62	1051.60		789.52	702.52	516.27		360.42	289.36	

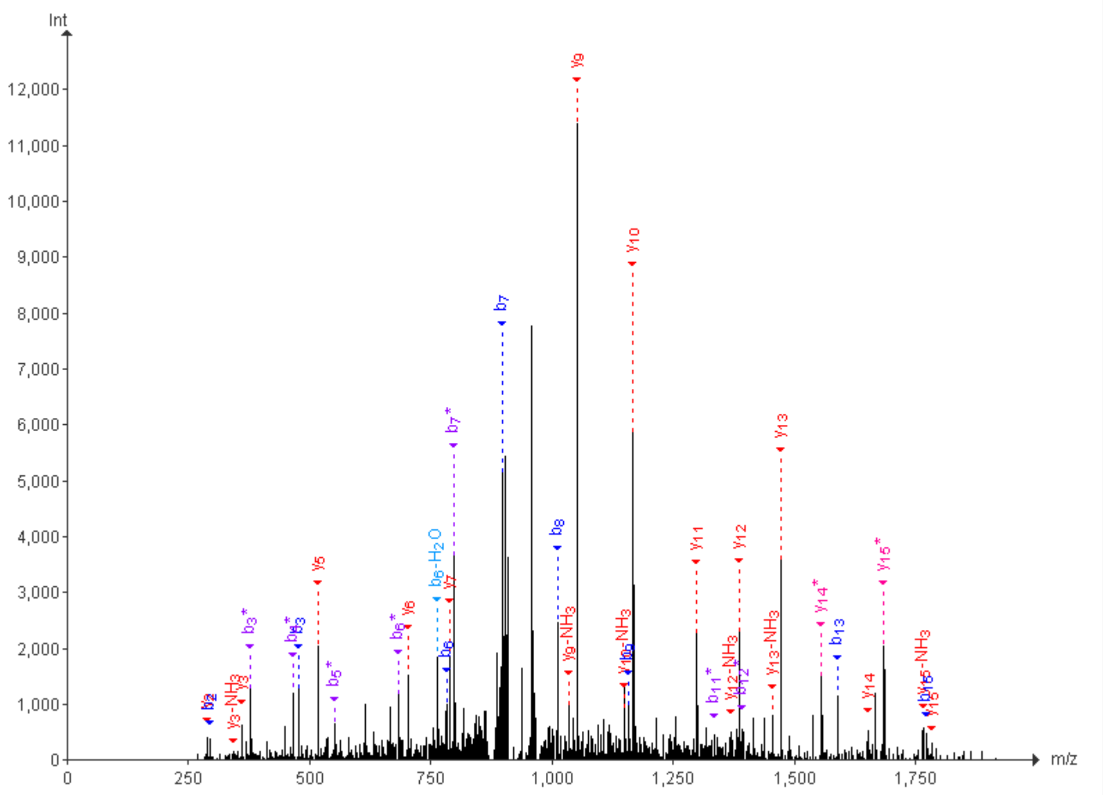

Precursor M/Z: 959.87492 (CID) Resolution: 2.86

Protein:	gi|307109275|gb|EFN57513.1| hypothetical protein CHLNCDRAFT_57286, containing cd04301 N-Acyltransferase superfamily*
Score:	71.394
Source:	phosphopeptide-1000mM-B2
Scan number:	1021

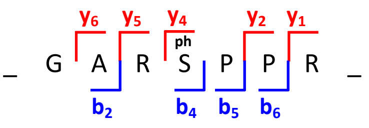

	157.21		480.31	577.46	576.52	
	b_2		b_4	b_5	b_6	
**G**	**A**	**R**	**S^{ph}**	**P**	**P**	**R**
	y_6	y_5	y_4		y_2	y_1
	763.58	692.53	536.29		272.39	175.18

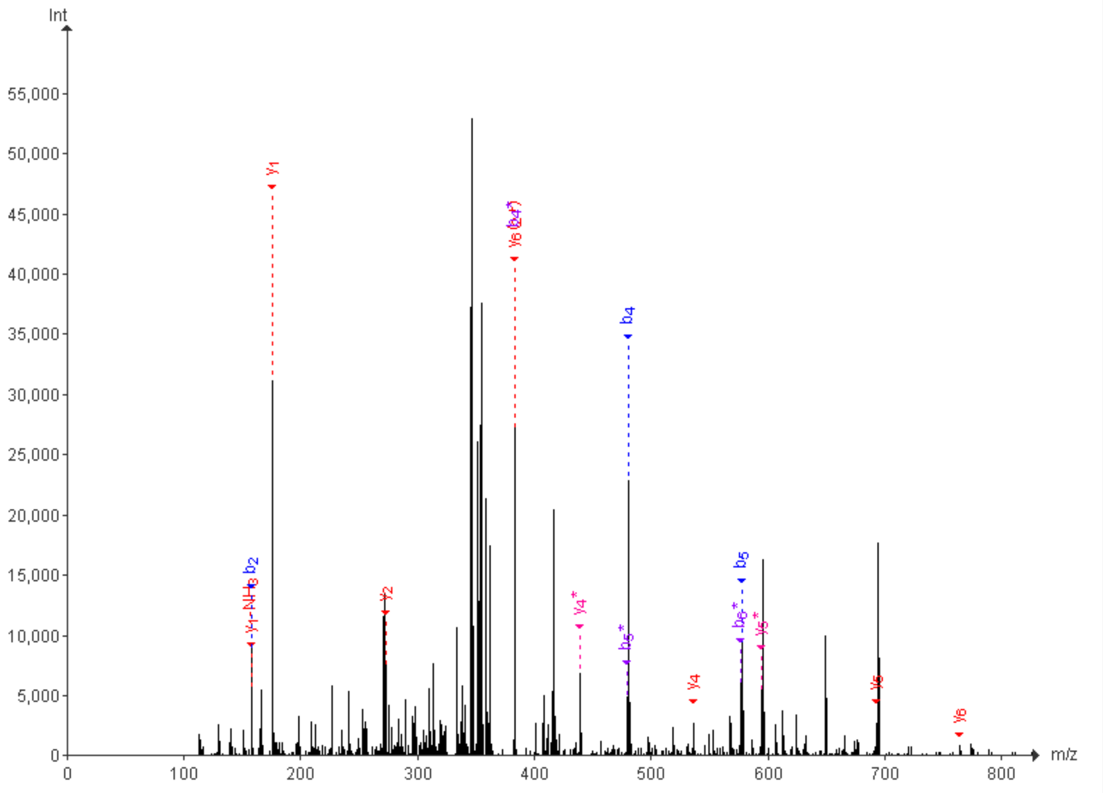

Protein:	gi|300261198|gb|EFJ45412.1| hypothetical protein VOLCADRAFT_82285, containing smart00658 RNA polymerase subunit 8 domain*
Score:	99.013
Source:	phosphopeptide-100mM-2
Scan number:	27359

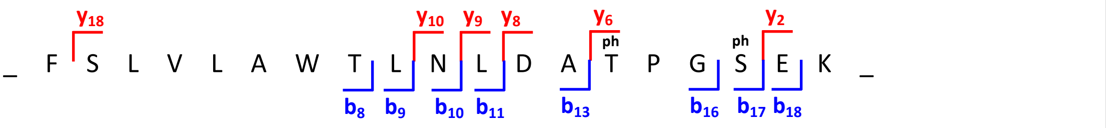

							474.03	1059.62	1173.77	1286.88		1472.92			904.55	988.04	1052.49	
							b_8^{2+}	b_9	b_{10}	b_{11}		b_{13}			b_{16}	b_{17}^{2+}	b_{18}^{2+}	
**F**	**S**	**L**	**V**	**L**	**A**	**W**	**T**	**L**	**N**	**L**	**D**	**A**	**T^{ph}**	**P**	**G**	**S^{ph}**	**E**	**K**
	y_{18}^{2+}								y_{10}	y_9	y_8		y_6				y_2	
	1052.49								1219.75	1105.69	992.56		806.61				304.37	

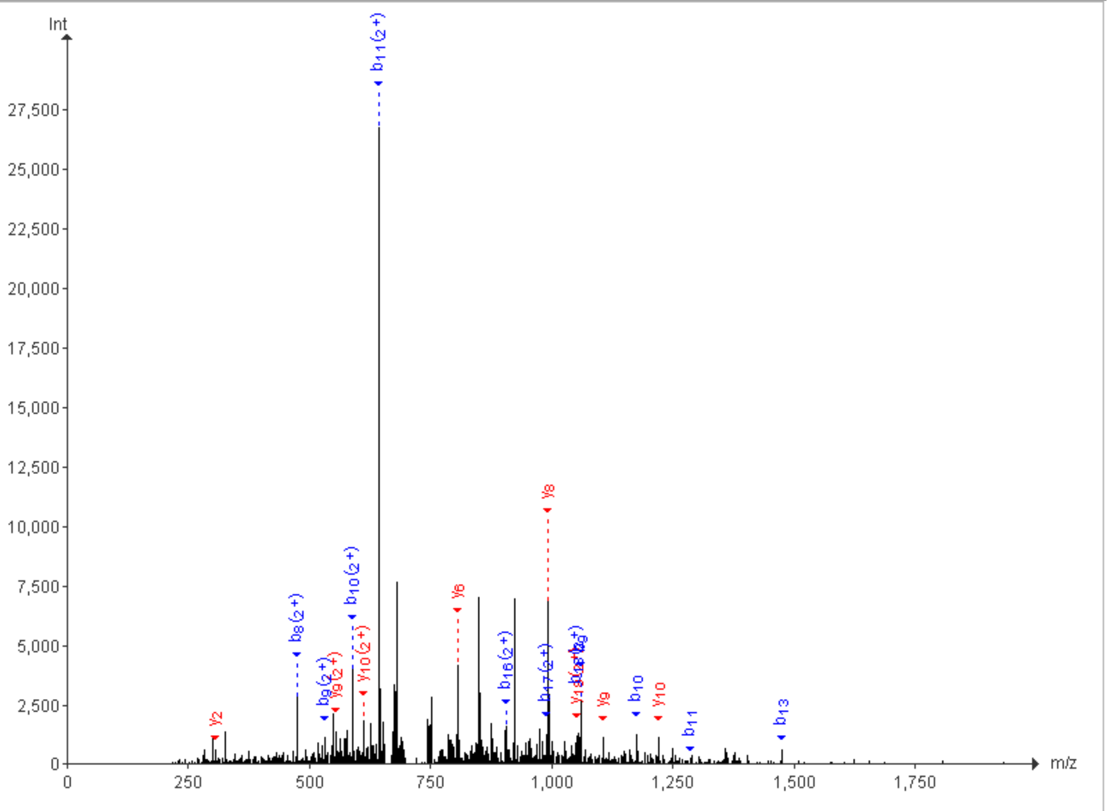

Precursor M/Z: 741.34425 (CID) Resolution: 2.86

Protein:	gi|300263278|gb|EFJ47479.1| hypothetical protein VOLCADRAFT_120953, containing cd00268 DEAD-box helicases domain*
Score:	91.119
Source:	phosphopeptide-1000mM-2
Scan number:	6544

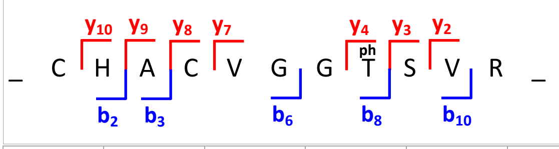

	326.36	397.30			713.42		853.50		1137.68	
	b_2	b_3			b_6		b_8*		b_{10}	
**C**	**H**	**A**	**C**	**V**	**G**	**G**	**T^{ph}**	**S**	**V**	**R**
	y_{10}	y_9	y_8	y_7			y_4*	y_3	y_2	
	1123.66	986.49	915.57	755.44			444.54	361.38	274.40	

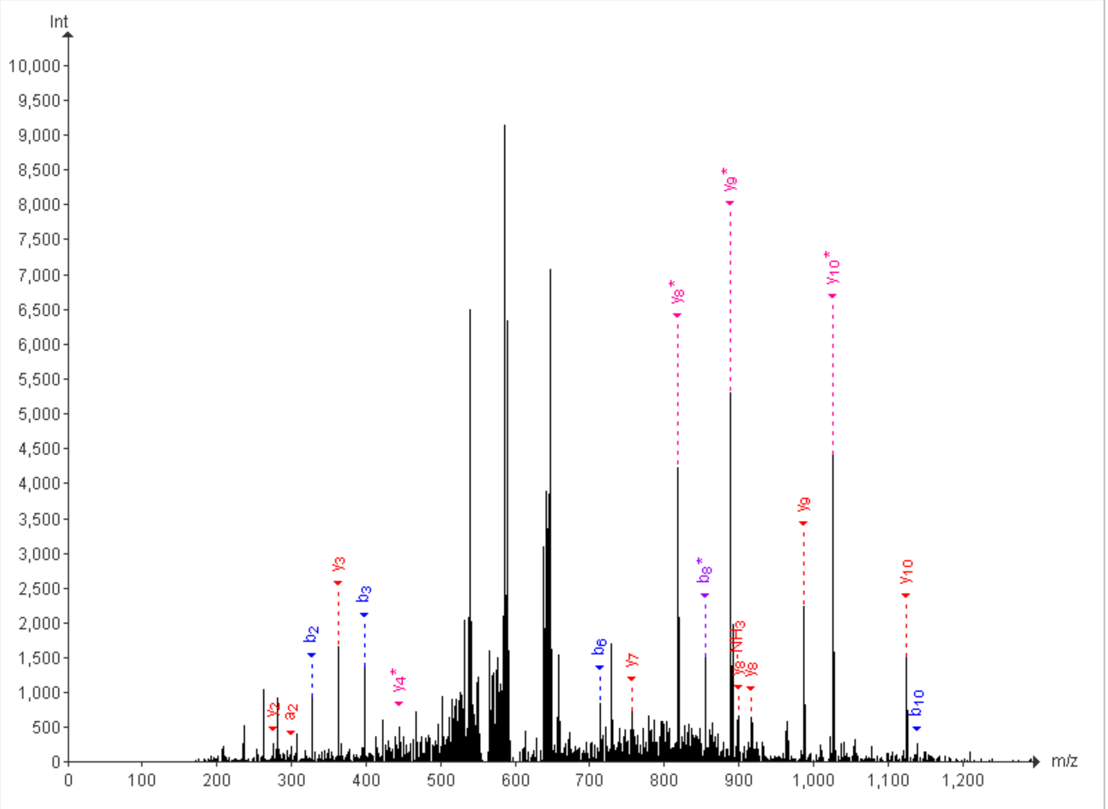

Protein:	gi|307110542|gb|EFN58778.1| hypothetical protein CHLNCDRAFT_34097, containing pfam06424 PRP1 splicing factor domain*
Score:	140.74
Source:	phosphopeptide-40mM-2
Scan number:	18570

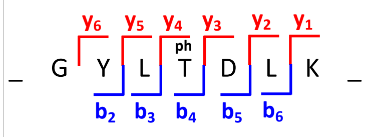

	249.21	362.34	543.33	658.32	771.37	
	b_2	b_3	b_4	b_5	b_6	
**G**	**Y**	**L**	**T^{ph}**	**D**	**L**	**K**
	y_6	y_5	y_4	y_3	y_2	y_1
	860.54	697.51	584.51	403.42	288.34	175.25

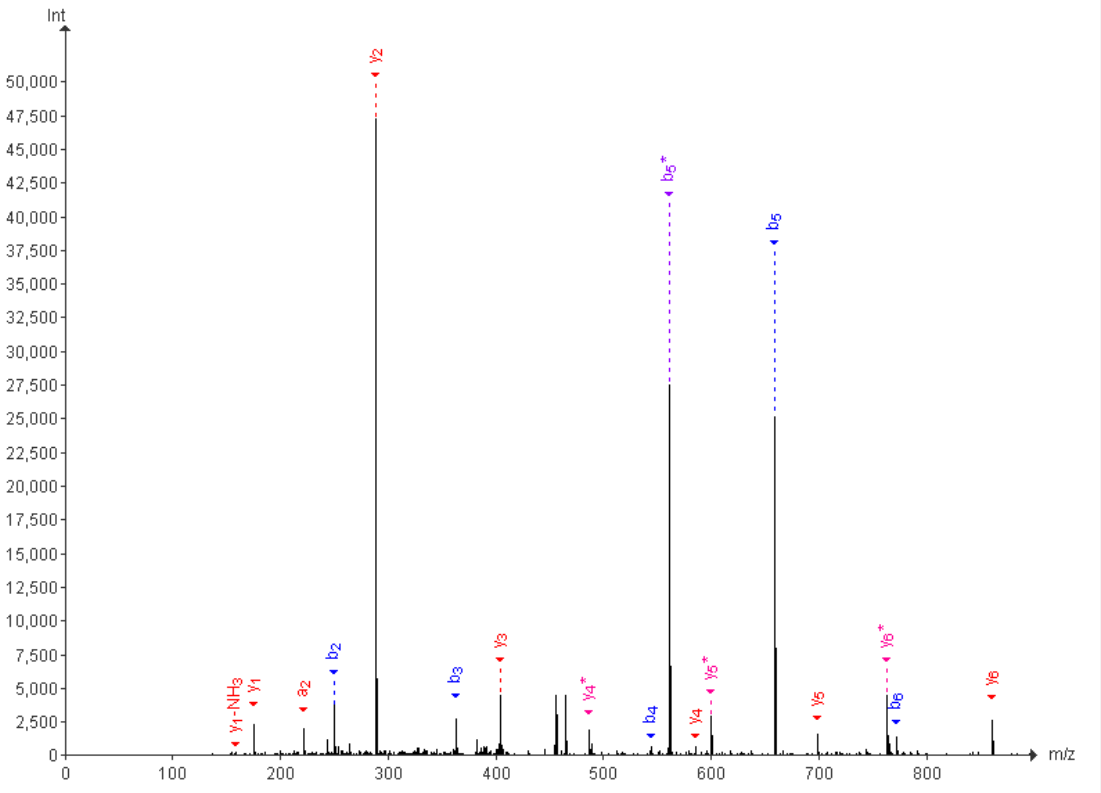

Precursor M/Z: 445.20698 (CID) Resolution: 1.29

Protein:	gi|226455398|gb|EEH52701.1| set domain protein
Score:	83.049
Source:	phosphopeptide-0mM-2
Scan number:	17730

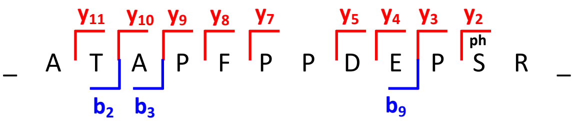

	201.31	272.41						954.66			
	b_2	b_3						b_9			
**A**	**T**	**A**	**P**	**F**	**P**	**P**	**D**	**E**	**P**	**S^{ph}**	**R**
	y_{11}	y_{10}*	y_9	y_8	y_7^{2+}		y_5	y_4*	y_3	y_2	
	1293.74	1094.64	1121.63	1024.69	439.32		683.51	470.62	439.32	342.39	

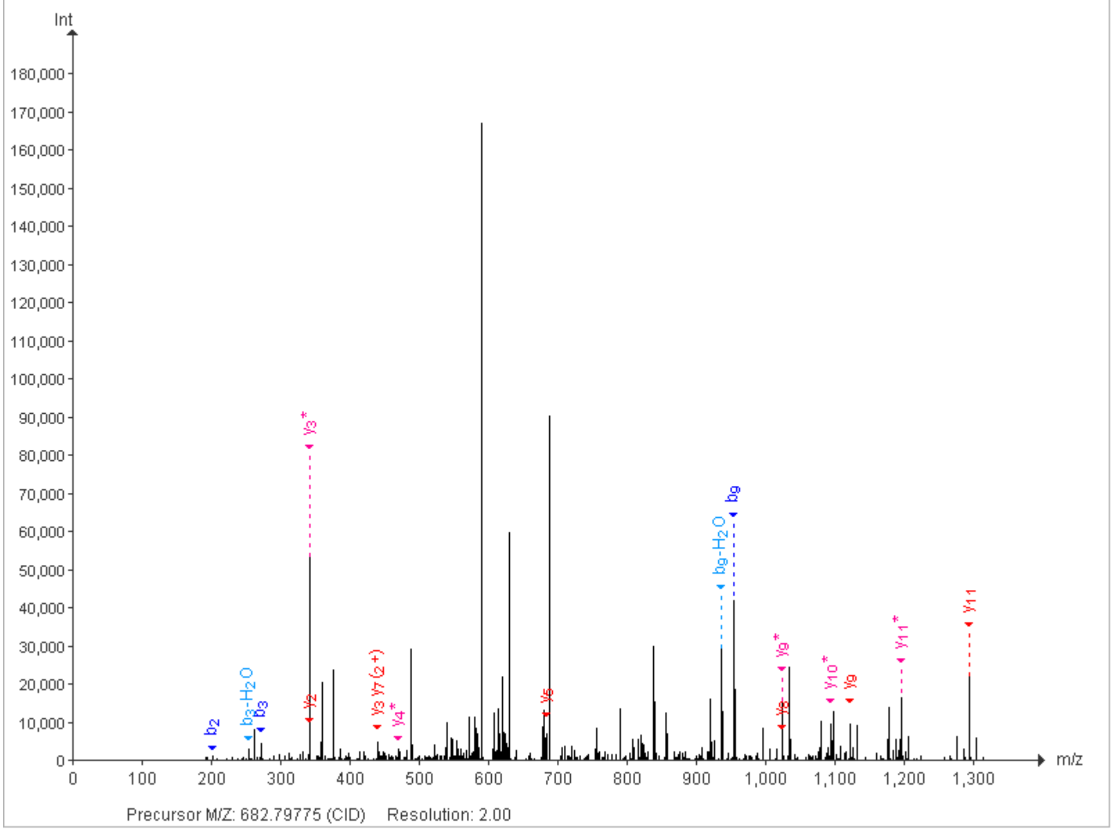

Protein:	gi|158283213|gb|EDP08964.1| ribosomal protein S3a
Score:	118.06
Source:	phosphopeptide-40mM-2
Scan number:	23120

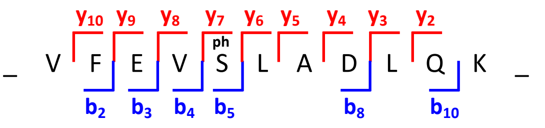

	279.41	408.42	507.50	576.48			973.68		1214.89	
	b_2	b_3	b_4	b_5*			b_8		b_{10}	
**V**	**F**	**E**	**V**	**S^{ph}**	**L**	**A**	**D**	**L**	**Q**	**K**
	y_{10}	y_9	y_8	y_7	y_6	y_5	y_4	y_3	y_2	
	1261.80	1114.78	985.70	886.65	719.53	606.45	535.47	420.47	307.48	

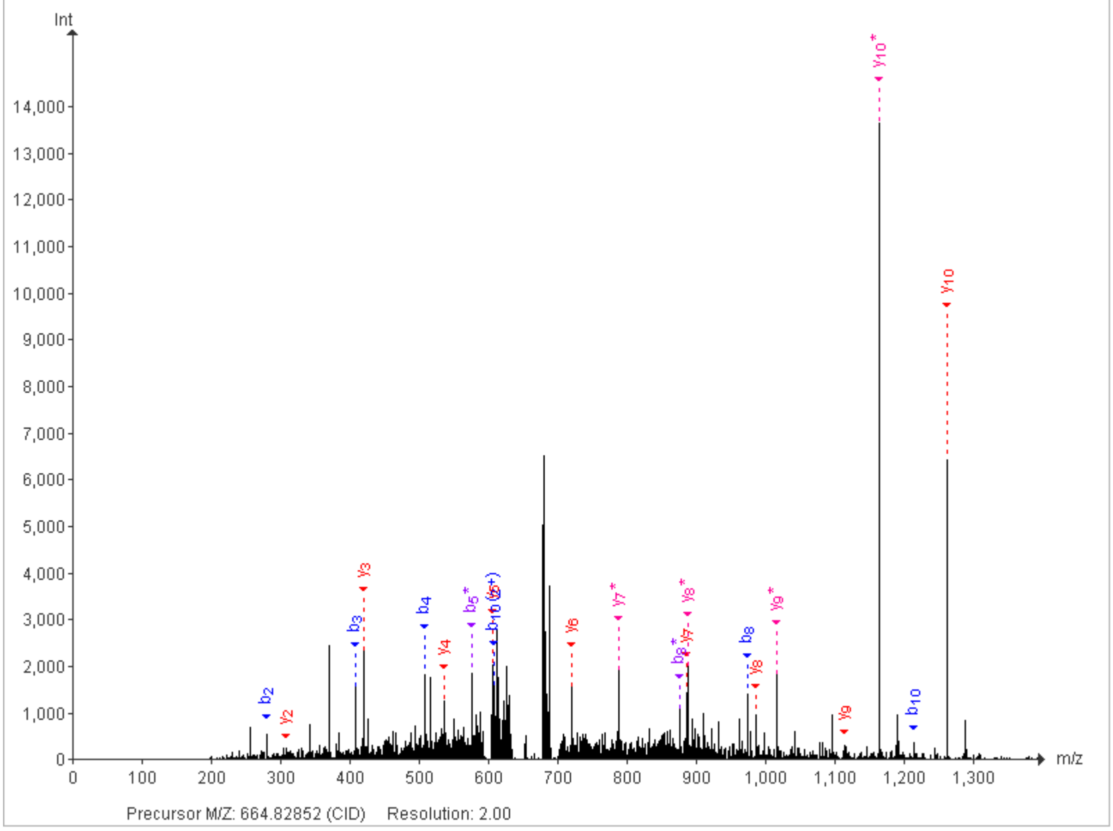

Protein:	gi|158276036|gb|EDP01810.1| ribosomal protein S6
Score:	99.276
Source:	phosphopeptide-1000mM-B2
Scan number:	15772

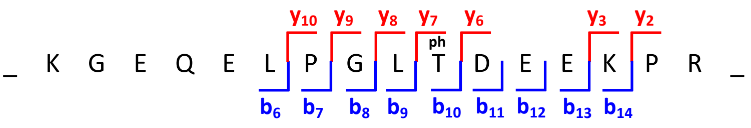

					749.55	846.70	903.70	1016.71	1099.79	1214.81	1441.81	1570.86	1633.16		
					b_6	b_7	b_8	b_9	b_{10}*	b_{11}*	b_{12}	b_{13}	b_{14}*		
**K**	**G**	**E**	**Q**	**E**	**L**	**P**	**G**	**L**	**T^{ph}**	**D**	**E**	**E**	**K**	**P**	**R**
						y_{10}*	y_9	y_8	y_7*	y_6			y_3	y_2	
						1155.78	1156.73	1099.79	888.56	805.52			432.40	272.35	

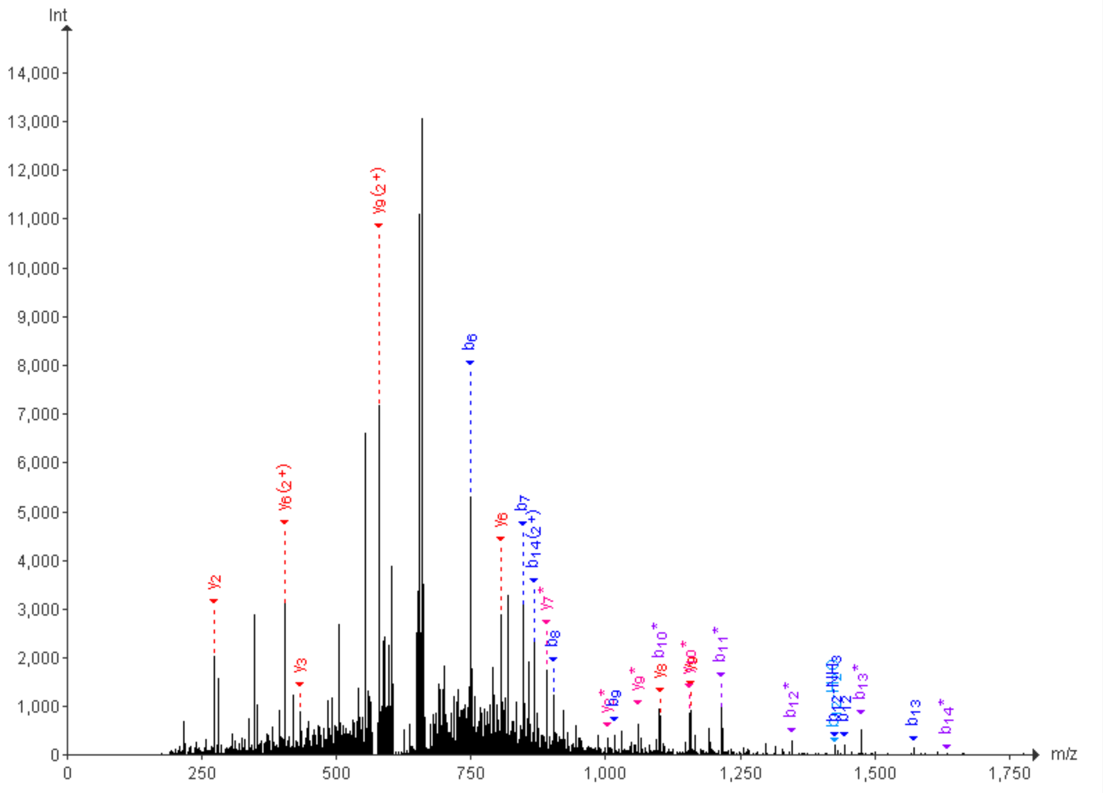

Precursor M/Z: 635.96997 (CID) Resolution: 2.57

Protein:	gi|246880774|gb|ACS95086.1| ribosomal protein S9
Score:	208.81
Source:	phosphopeptide-40mM-2
Scan number:	17582

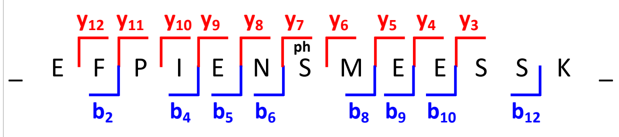

	305.33		515.33	644.43	758.50		958.55	1185.54	1314.56		1488.84	
	b_2		b_4	b_5	b_6		b_8*	b_9	b_{10}		b_{12}	
**E**	**F**	**P**	**I**	**E**	**N**	**S^{ph}**	**M**	**E**	**E**	**S**	**S**	**K**
	y_{12}	y_{11}	y_{10}	y_9	y_8	y_7	y_6	y_5	y_4	y_3		
	1505.77	1358.65	1261.71	1148.56	1019.40	905.54	738.50	607.37	478.43	349.38		

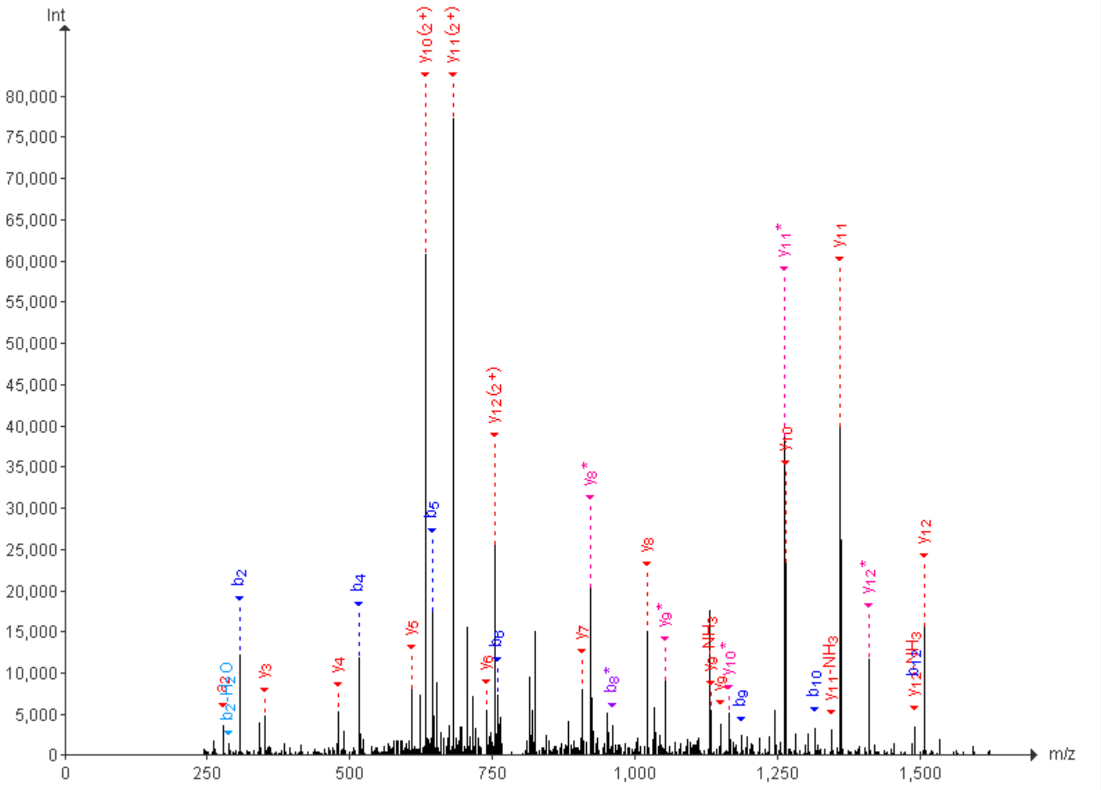

Precursor M/Z: 803.82076 (CID)   Resolution: 2.43

Protein:	gi|158274884|gb|EDP00664.1| ribosomal protein L12
Score:	105.38
Source:	phosphopeptide-24mM-2
Scan number:	12376

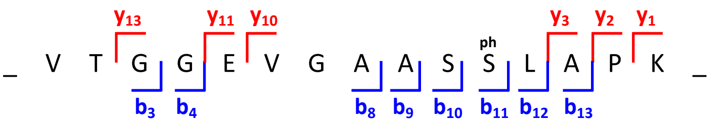

		290.35	347.43				703.51	774.61	861.63	1028.44	571.46	1212.75		
		b_3	b_4				b_8*	b_9	b_{10}	b_{11}	b_{12}^{2+}	b_{13}		
**V**	**T**	**G**	**G**	**E**	**V**	**G**	**A**	**A**	**S**	**S^ph^**	**L**	**A**	**P**	**K**
		y_{13}^{2+}		y_{11}^{2+}	y_{10}							y_3	y_2	y_1
		628.43		571.46	1012.40							347.43	276.35	179.21

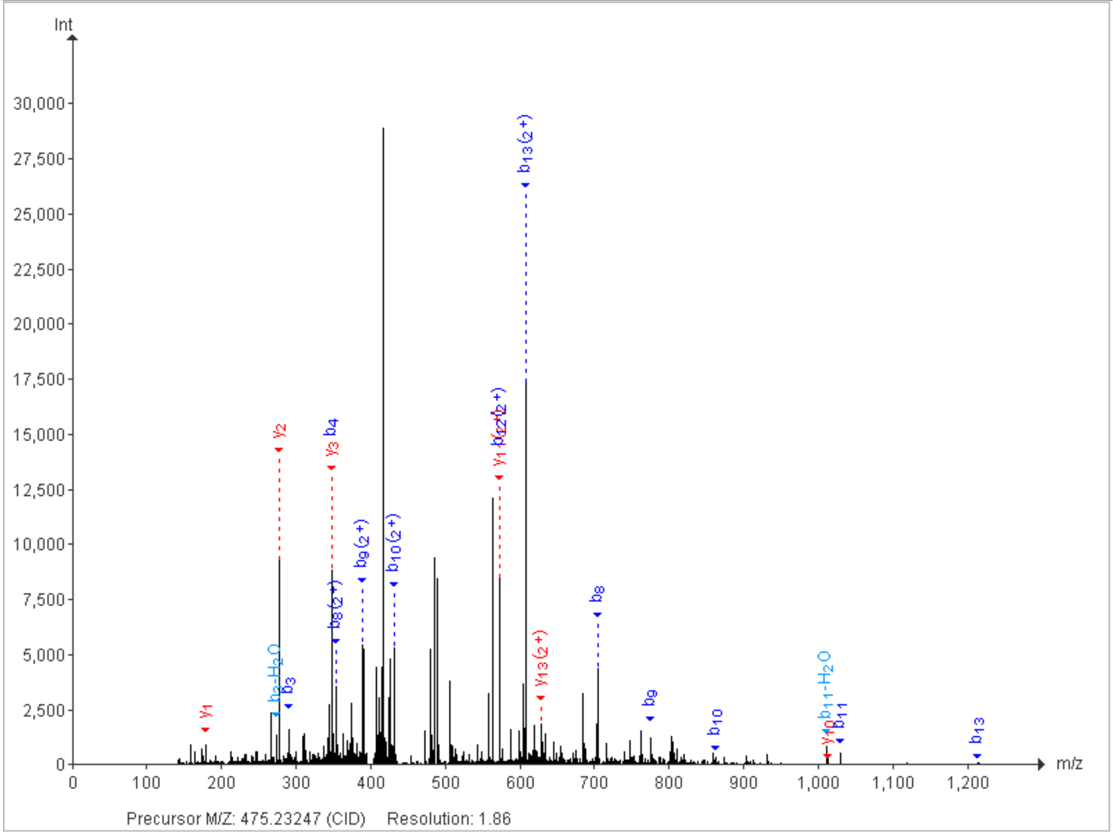

Protein:	gi|300266633|gb|EFJ50819.1| hypothetical protein VOLCADRAFT_120515, containing cd00632 prefoldin beta domain*
Score:	86.882
Source:	phosphopeptide-0mM-b2
Scan number:	9629

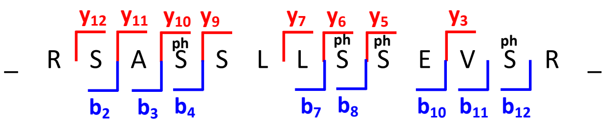

	272.31	343.37	510.29			412.33	892.45		1286.80	1189.61	1552.80	
	b_2	b_3	b_4			b_7^{2+}	b_8*		b_{10}	b_{11}*	b_{12}	
**R**	**S**	**A**	**S^{ph}**	**S**	**L**	**L**	**S^{ph}**	**S^{ph}**	**E**	**V**	**S^{ph}**	**R**
	y_{12}	y_{11}	y_{10}*	y_9		y_7	y_6*	y_5*		y_3*		
	1542.70	1455.81	1286.80	1119.58		1017.59	708.51	639.52		343.37		

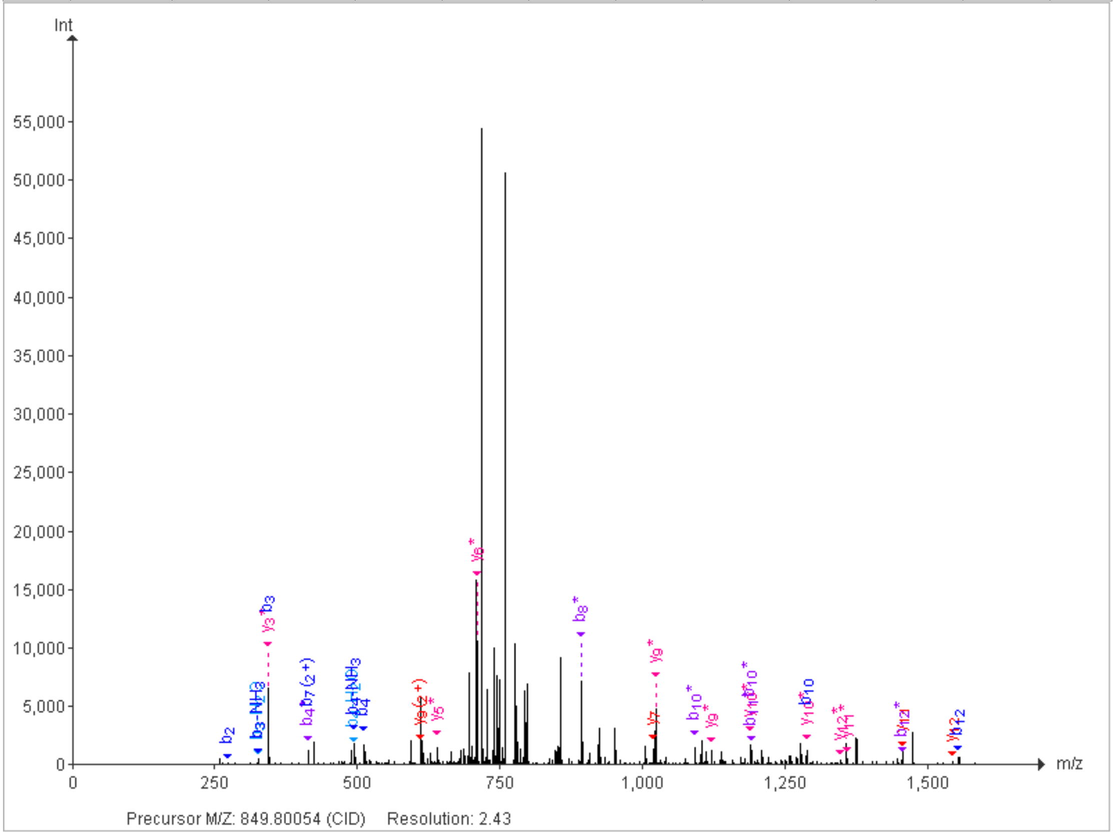

Protein:	gi|307111867|gb|EFN60101.1| hypothetical protein CHLNCDRAFT_133427, containing cd00118 Lysin motif domain*
Score:	86.91
Source:	phosphopeptide-56mM-2
Scan number:	12338

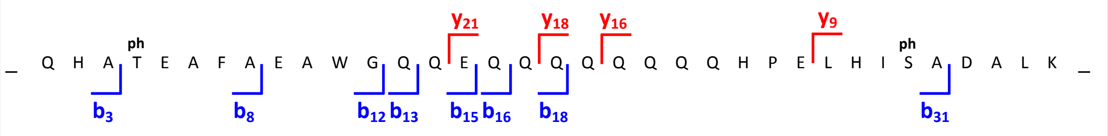

		369.30					484.74				706.68	769.92		898.80	963.27		1091.35													1892.98				
		b_3					b_8^{2+}				b_{12}^{2+}	b_{13}^{2+}		b_{15}^{2+}	b_{16}^{2+}		b_{18}^{2+}													b_{31}^{2+}				
Q	H	A	T^{ph}	E	A	F	A	E	A	W	G	Q	Q	E	Q	Q	Q	Q	Q	Q	Q	Q	H	P	E	L	H	I	S^{ph}	A	D	A	L	K
														y_{21}^{2+}			y_{18}^{2+}		y_{16}^{2+}							y_9^{2+}								
														1298.68			1105.62		977.80							540.28								

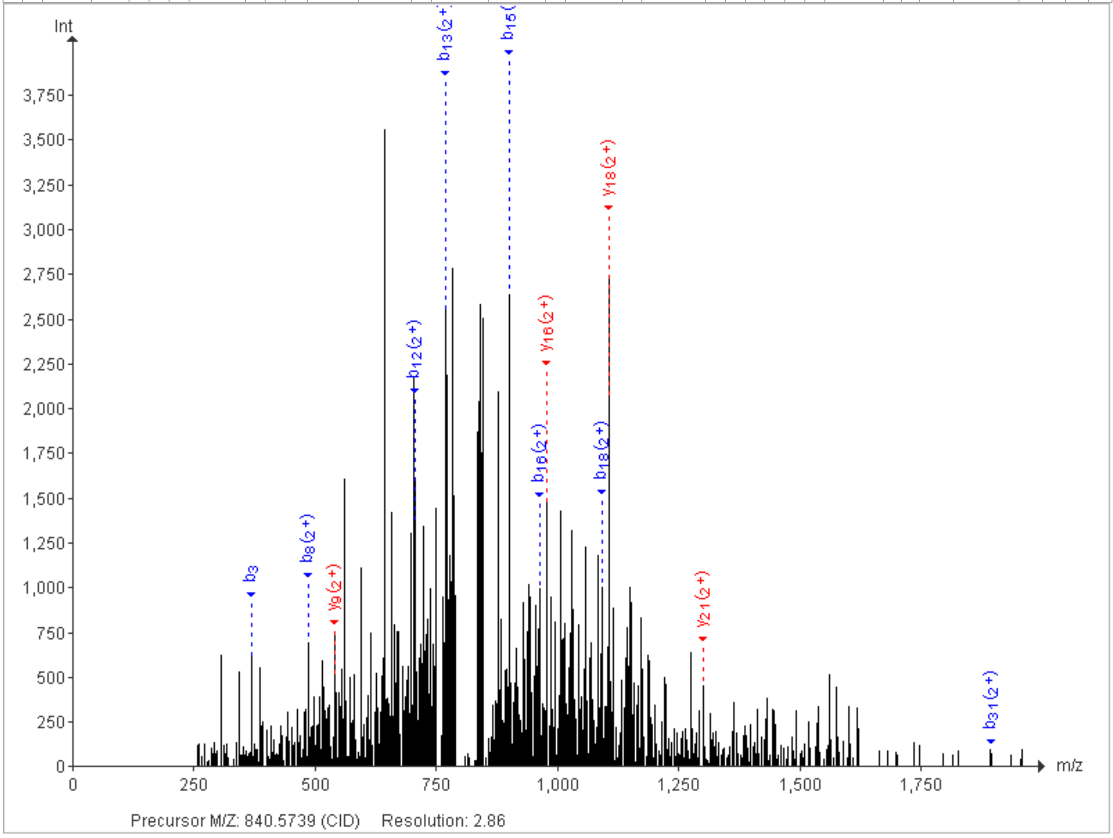

Protein:	gi|300256553|gb|EFJ40816.1| hypothetical protein VOLCADRAFT_109434, containing cd00195 Ubiquitin-conjugating enzyme E2 catalytic domain*
Score:	70.263
Source:	phosphopeptide-1000mM-B2
Scan number:	13455

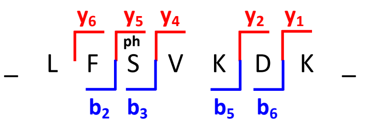

	293.37	362.43		621.64	834.53	
	b_2	b_3*		b_5*	b_6	
**L**	**F**	**S^{ph}**	**V**	**K**	**D**	**K**
	y_6	y_5	y_4		y_2	y_1
	867.57	720.59	553.39		294.39	179.20

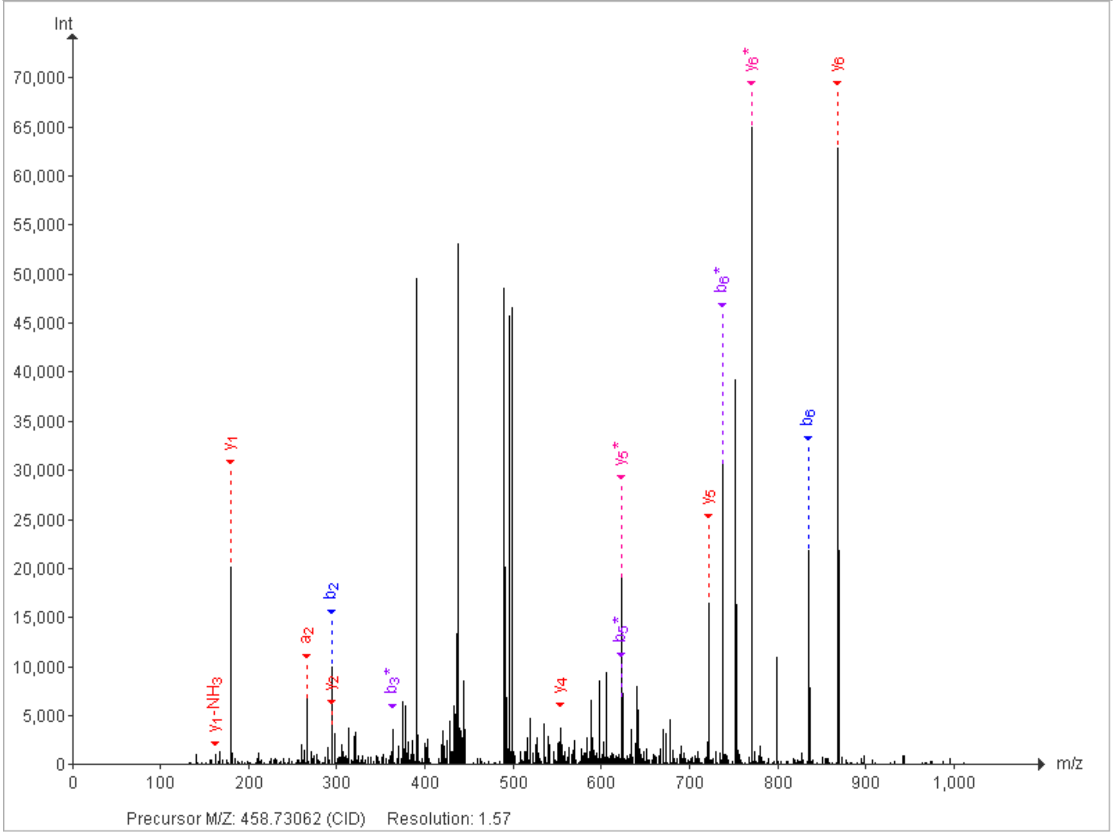

Protein:	gi|158279575|gb|EDP05335.1| membrane AAA-metalloprotease
Score:	174.74
Source:	phosphopeptide-56mM-B2
Scan number:	16725

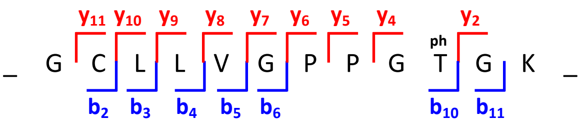

	250.29	363.36	476.39	575.43	632.43				1064.63	1121.71	
	b_2	b_3	b_4	b_5	b_6				b_{10}	b_{11}	
**G**	**C**	**L**	**L**	**V**	**G**	**P**	**P**	**G**	**T^{ph}**	**G**	**K**
	y_{11}	y_{10}	y_9	y_8	y_7	y_6	y_5	y_4*		y_2	
	1210.89	1050.79	937.69	824.57	725.46	668.42	571.42	376.43		236.25	

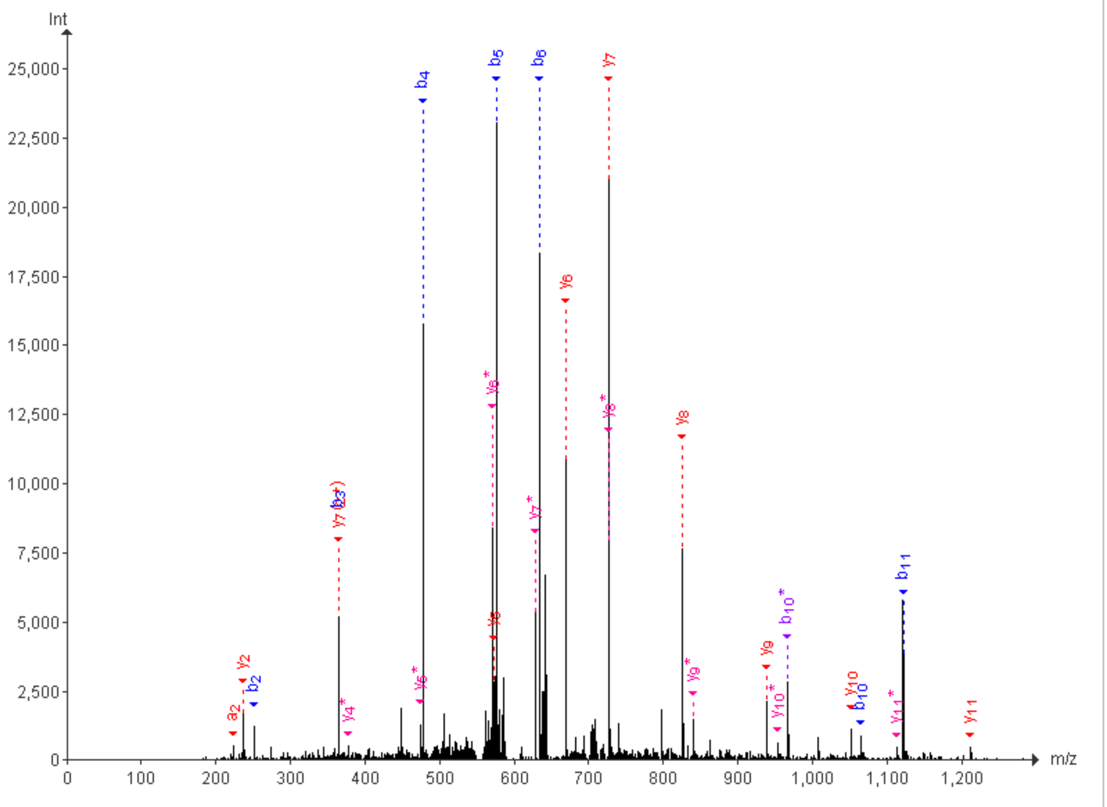

Protein:	gi|300256509|gb|EFJ40773.1| hypothetical protein VOLCADRAFT_84366, containing cd07419 Arabidopsis thaliana Bsu1 phosphatase and related proteins, C-terminal metallophosphatase domain*
Score:	171.43
Source:	phosphopeptide-40mM-B2
Scan number:	15961

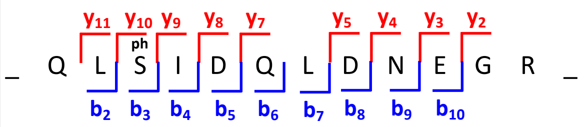

	270.33	339.42	550.35	665.40	793.50	906.49	1021.47	568.44	1166.75		
	b_2	b_3*	b_4	b_5	b_6	b_7	b_8	b_9^{2+}	b_{10}*		
**Q**	**L**	**S^{ph}**	**I**	**D**	**Q**	**L**	**D**	**N**	**E**	**G**	**R**
	y_{11}	y_{10}	y_9	y_8	y_7		y_5	y_4	y_3	y_2	
	1339.71	1226.62	1059.64	946.49	831.47		590.31	475.26	361.36	232.32	

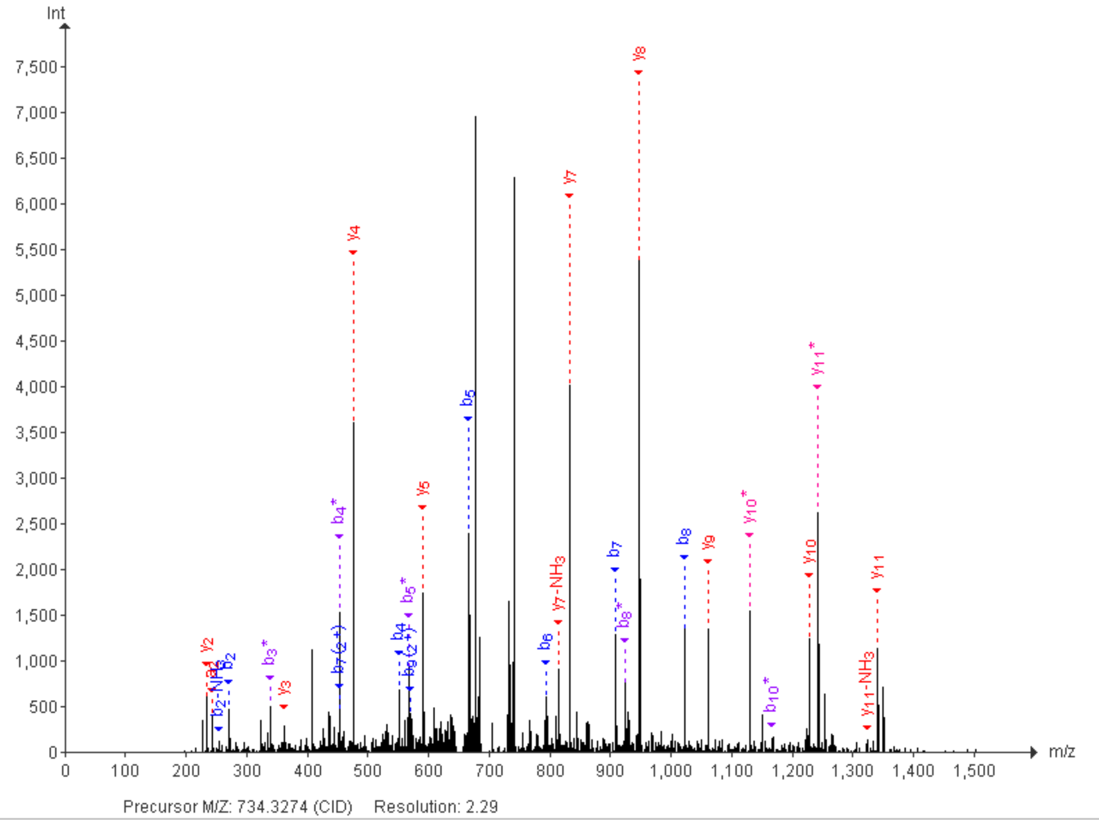

Protein:	gi|158271168|gb|EDO96994.1| Snf1-like protein kinase
Score:	143.63
Source:	phosphopeptide-0mM-B3
Scan number:	10091

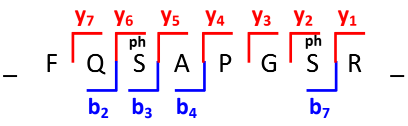

	304.30	373.39	542.40			863.58	
	b_2	b_3*	b_4			b_7	
**F**	**Q**	**S^{ph}**	**A**	**P**	**G**	**S^{ph}**	**R**
	y_7	y_6*	y_5	y_4*	y_3*	y_2*	y_1
	862.38	636.39	567.41	398.38	301.31	244.24	175.19

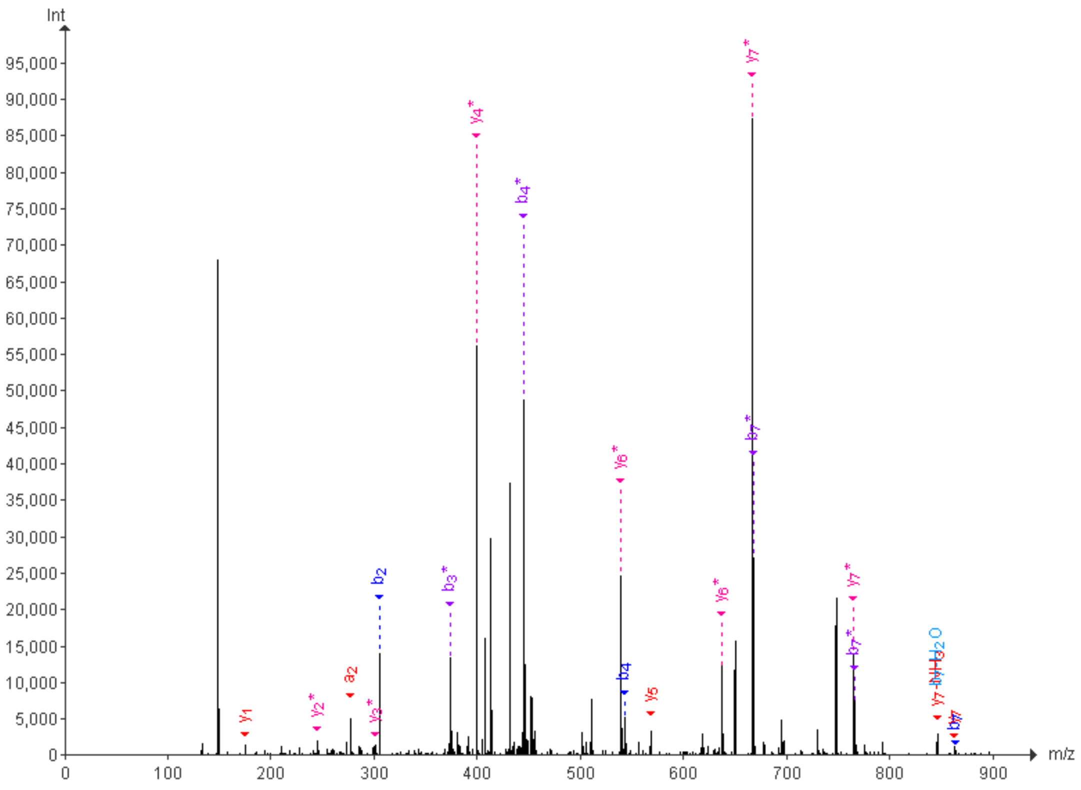

Precursor M/Z: 505.18064 (CID) Resolution: 1.34

Protein:	gi|307105164|gb|EFN53414.1| hypothetical protein CHLNCDRAFT_136605, containing cd14137 glycogen synthase kinase 3*
Score:	46.462
Source:	phosphopeptide-56mM-2
Scan number:	16042

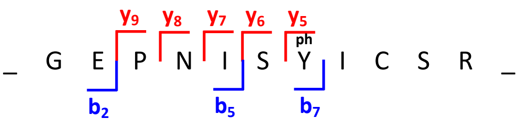

	219.31			543.34		873.36				
	b_2			b_5		b_7				
**G**	**E**	**P**	**N**	**I**	**S**	**Y^{ph}**	**I**	**C**	**S**	**R**
		y_9	y_8	y_7	y_6	y_5				
		1189.62	1092.76	978.57	865.51	778.48				

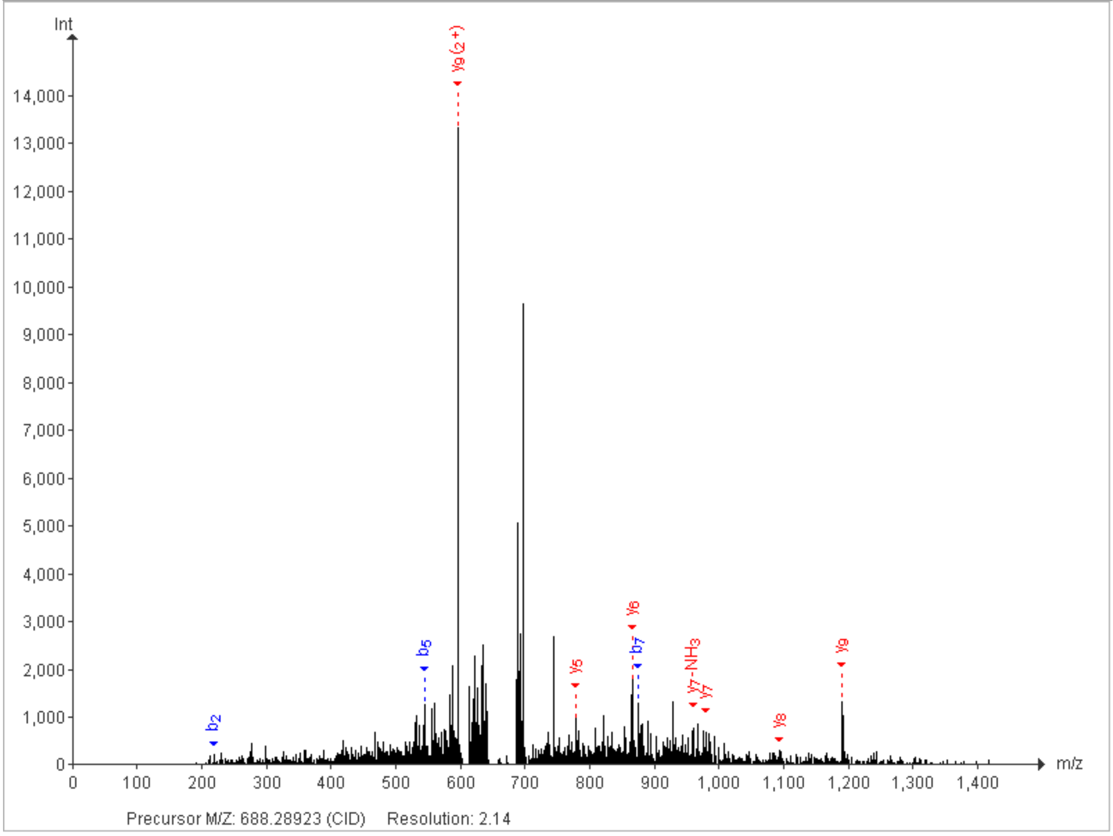

Protein:	gi|300266429|gb|EFJ50616.1| hypothetical protein VOLCADRAFT_116796, containing cd00173 Src homology 2 domain*
Score:	119.33
Source:	phosphopeptide-100mM-2
Scan number:	8054

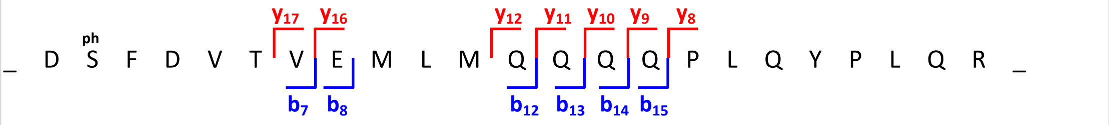

						872.73	501.31				753.22	817.26	880.98	945.31								
						b_7	b_8^{2+}				b_{12}^{2+}	b_{13}^{2+}	b_{14}^{2+}	b_{15}^{2+}								
**D**	**S^{ph}**	**F**	**D**	**V**	**T**	**V**	**E**	**M**	**L**	**M**	**Q**	**Q**	**Q**	**Q**	**P**	**L**	**Q**	**Y**	**P**	**L**	**Q**	**R**
						y_{17}^{2+}	y_{16}^{2+}				y_{12}	y_{11}	y_{10}	y_9	y_8							
						1065.51	1015.54				1526.88	1398.74	1270.66	1142.73	1014.60							

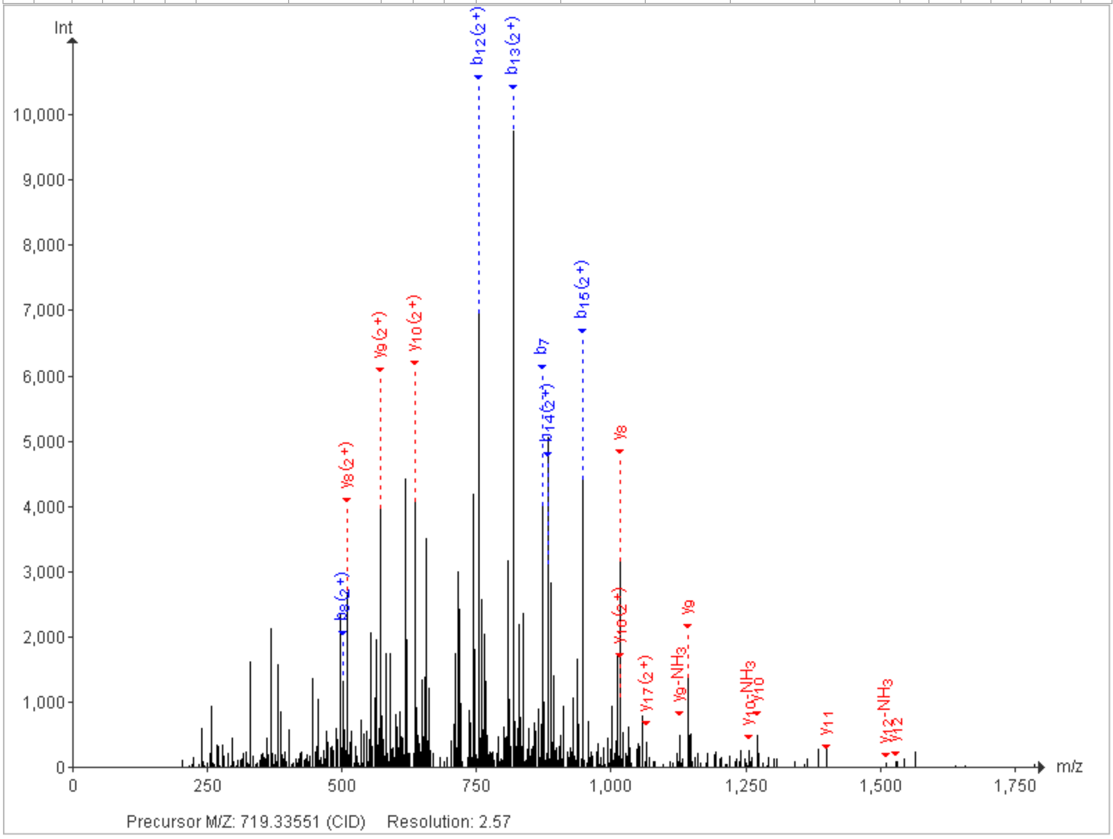

Protein:	gi|226455999|gb|EEH53301.1| predicted protein
Score:	90.814
Source:	phosphopeptide-56mM-B3
Scan number:	13762

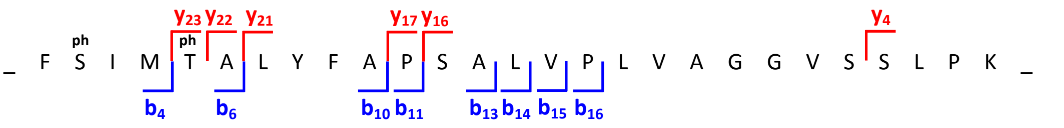

			587.31		420.43				1333.76	1430.80		1668.99	1782.13	1881.16	1978.04											
			b_4		b_6^{2+}				b_{10}	b_{11}		b_{13}	b_{14}	b_{15}	b_{16}											
F	S^{ph}	I	M	T^{ph}	A	L	Y	F	A	P	S	A	L	V	P	L	V	A	G	G	V	S	S	L	P	K
				y_{23}^{2+}	y_{22}^{2+}	y_{21}^{2+}				y_{17}^{2+}	y_{16}^{2+}												y^4			
				1263.96	1173.38	1137.96				890.93	842.32												472.40			

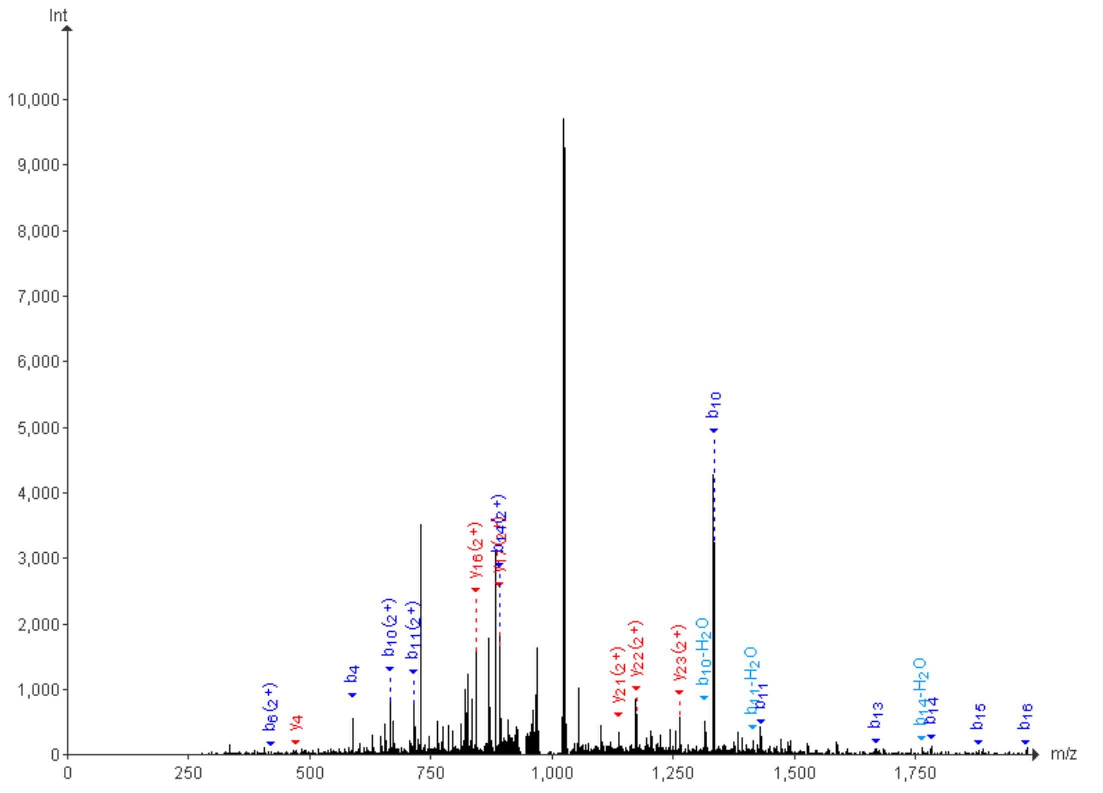

Precursor M/Z: 1019.4614 (CID) Resolution: 2.86

Protein:	gi|226457710|gb|EEH55008.1| predicted protein
Score:	80.584
Source:	phosphopeptide-0mM-B3
Scan number:	2744

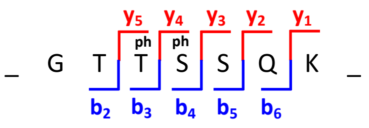

	191.17	274.24	343.34	528.36	754.50	
	b_2	b_3*	b_4*	b_5*	b_6	
**G**	**T**	**T^{ph}**	**S^{ph}**	**S**	**Q**	**K**
		y_5*	y_4	y_3	y_2	y_1
		644.50	561.34	394.43	307.37	179.34

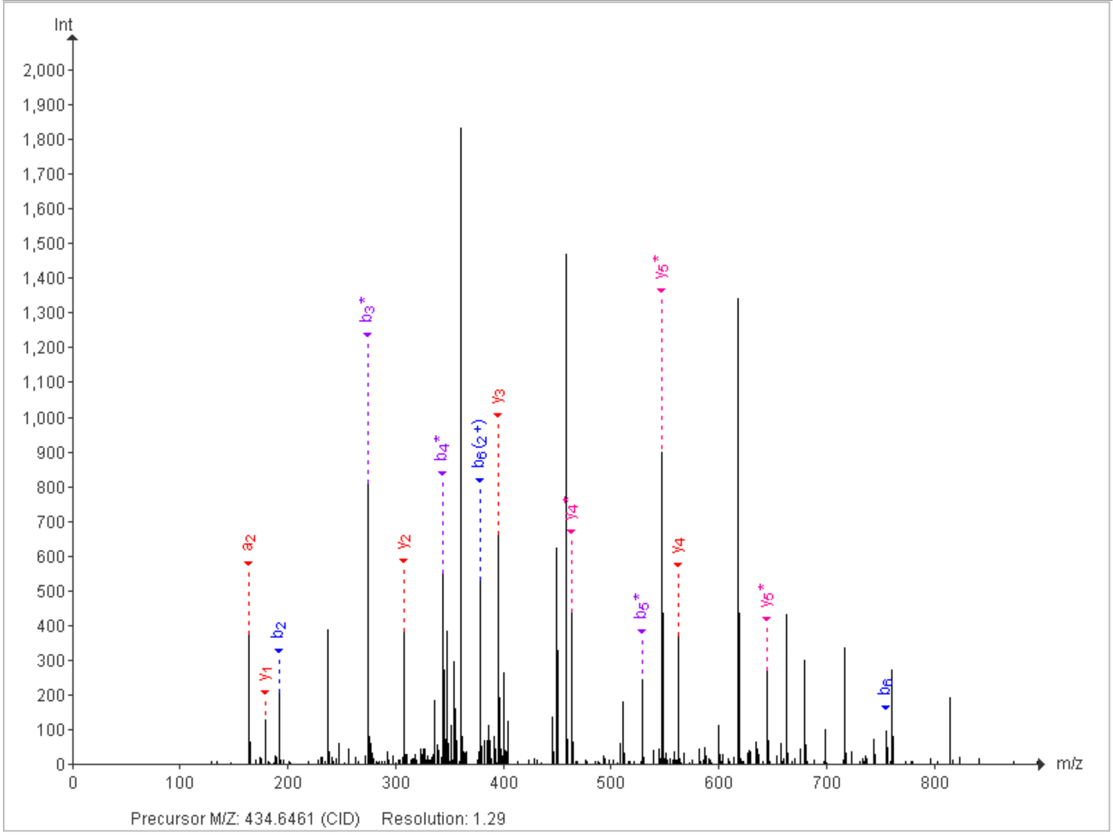

Protein:	gi|300267726|gb|EFJ51908.1| hypothetical protein VOLCADRAFT_86882
Score:	133.47
Source:	phosphopeptide-24mM-B3
Scan number:	12220

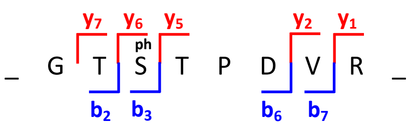

	187.25	354.25			667.46	766.39	
	b_2	b_3			b_6	b_7	
**G**	**T**	**S^{ph}**	**T**	**P**	**D**	**V**	**R**
	y_7	y_6	y_5			y_2	y_1
	855.46	754.47	587.39			274.31	175.17

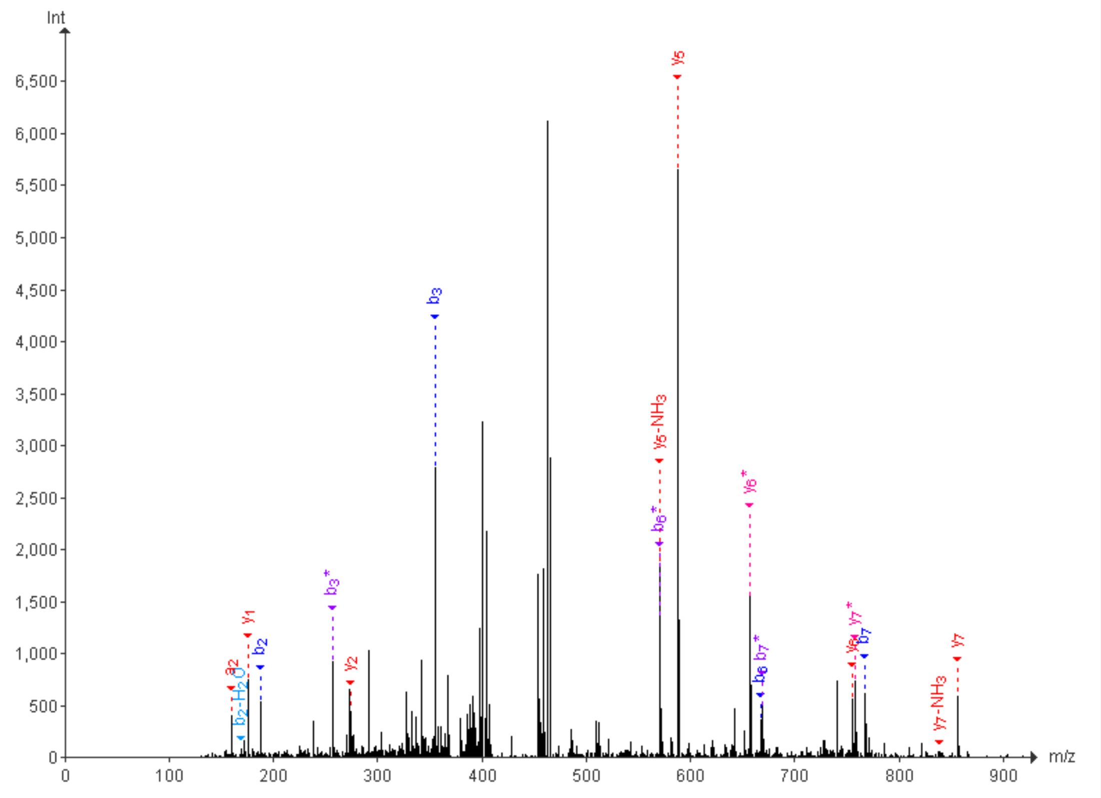

Supplement: Supplementary file 12 [file Image3.PDF]
