# Supplementary material for: Salinity-Induced Palmella Formation Mechanism in Halotolerant Algae Dunaliella salina Revealed by Quantitative Proteomics and Phosphoproteomics
Source: Front Plant Sci. 2017 May 23;8:810. doi: 10.3389/fpls.2017.00810 (PMC5441111; doi:10.3389/fpls.2017.00810)
Supplement: Supplementary file 13 [file Image4.PDF]

```
SsTUA         MRECISIHVGQAGVQIGNACWELYCLEHGIQPDGQMPSDKTIGGGDDSFNTFFSETGAGK
DsTubulin     MREAISIHIGQAGCQIGNTCWELYCLEHGIQPDGQMPSDKTIGGGDDAFNTFFSETGAGK
OsTUA2        MREIISIHIGQAGIQVGNSCWELYCLEHGIQPDGLMPSDTTPGIARDAFNTFFSETSSGK
OsTUA1        MREIISIHIGQAGIQVGNACWELYCLEHGIEPDGTMPSDTTVGVAHDAFNTFFSETGAGK
OsTUA3-1      MRECISIHIGQAGIQVGNACWELYCLEHGIQADGQMPSDRTVGGGDDAFNTFFSETGAGK
OsTUA3-2      MRECISIHIGQAGIQVGNACWELYCLEHGIQPDGQMPGDKTVGGGDDAFNTFFSETGAGK
              *** ****:**** *:**:***********: ** **.* * * . *:********.:**

SsTUA         HVPRAVFVDLEPTVIDEVRTGTYRQLFHPEQLITGKEDAANNYARGHYTIGKEIIDLVLD
DsTubulin     HVPRAIFLDLEPTVIDEVRTGTYRQLFHPEQLISGKEDAANNFARGHYTIGKEIVDLALD
OsTUA2        HVPRALFVDLEPTVIDEVKTGPYRQLFHPEQLISYKEDAANNFARGHYTVGREVVDLCLD
OsTUA1        HVPRAIFVDLEPTVIDEVRTGSYRQLFHPEQLISGKEDAANNFARGHYTVGKEIVDLCLD
OsTUA3-1      HVPRAVFVDLEPTVIDEVRTGCYRQLFHPEQLINGKEDAANNFARGHYTIGKEIVDLCLD
OsTUA3-2      HVPRAVFVDLEPTVIDEVRTGDYRQLFHPEQLISGKEDAANNFARGHYTIGKEIVDLCLD
              *****:*:**********:** ***********. *******:******:*:*::** **

SsTUA         RIRKLADQCTGLQGFSVFHSFGGGTGSGFTSLLMERLSVDYGKKSKLEFSIYPAPQVSTA
DsTubulin     RIRKLADNCTGLQGFLVFNAVGGGTGSGLGSLLLERLSVDYGKKSKLAFTVYPSPQVSTA
OsTUA2        RLRKLADNCTGLQGFLVFNAVGGGTGSGLGSLLLERLSVDYGRKSKLGFTIYPSPQISTA
OsTUA1        RVRKLADNCTGLQGFLVFNAVGGGTGSGLGSLLLERLSVDYGKKSKLGFTIYPSPQVSTA
OsTUA3-1      RIRKLADNCTGLQGFLVFNAVGGGTGSGLGSLLLERLSVDYGKKSKLGFTVYPSPQVSTS
OsTUA3-2      RIRKLADNCTGLQGFLVFNAVGGGTGSGLGSLLLERLSVDYGKKSKLGFTVYPSPQVSTS
              *:*****:******* **.:.*******: ***:********:**** *::**:**:**:

SsTUA         VVEPYNSILTTHTTLEHSDCAFMVDNEAIYDICRRNLDIERPTYTNLNRLIGQIVSSITA
DsTubulin     VVEPYNSVLSTHSLLEHTDVAIMLDNEAIYDICRRSLDIERPTYTNLNRLIAQVISSLTA
OsTUA2        VVEPYNSVLSTHSLIEHTDVVVLLDNEAIYDICKRSLDIERPTYTNLNRLISQVISSLTT
OsTUA1        VVEPYNSVLSTHSLLEHTDVAVLLDNEAIYDICRRSLDIERPTYTNLNRLISQIISSLTT
OsTUA3-1      VVEPYNSVLSTHSLLEHTDVAVLLDNEAIYDICRRSLDIERPTYTNLNRLVSQVISSLTA
OsTUA3-2      AVLLDNEAIYDICRRSTDVAVILLDNEAIYDICRRSLDIERPTYTNLNRLVSQVISSLTA
              .*    *. :        .     ..::*********:*.**************:.*::**:*:

SsTUA         SLRFDGALNVDLTEFQTNLVPYPRAHFPLATYAPVISAEKAYHEQLSVAEITNACFEPAN
DsTubulin     SLRFDGALNVDITEFQTNLVPYPRIHFVLSSYAPIISAEKAYHEQLSVAEITNAVFEPAS
OsTUA2        SLRFDGAINVDITEFQTNLVPYPRIHFMLSSYAPIISVEKAFHEQHSVPEITNSVFEPAS
OsTUA1        SLRFDGAINVDVTEFQTNLVPYPRIHFMLSSYAPVISAEKAYHEQLSVPEITNAVFEPSS
OsTUA3-1      SLRFDGALNVDVNEFQTNLVPYPRIHFMLSSYAPVISAEKAYHEQLSVAEITNSAFEPSS
OsTUA3-2      SLRFDGALNVDVNEFQTNLVPYPRIHFMLSSYAPVISAEKAYHEQLSVAEITNSAFEPSS
              *******:***:.*********** ** *::***:**.***:*** **** ****: ***:.

SsTUA         QMVKCDPRHGKYMACCLLYRGDVVPKDVNAAIATIKTKRTIQFVDWCPTGFKYGINYEPP
DsTubulin     MMVKCDPRHGKYMACCLMYRGDVVPKDVNAAVATIKTKRTIQFVDWCPTGFKCGINYQPP
OsTUA2        VMAKCDPRHGKYMACCLMYRGDVVPKDVNAAVHSIKTRRTVQFVDWCPTGFKCGINYQPP
OsTUA1        MMAKCDPRHGKYMACCLMYRGDVVPKDVNAAVATIKTKRTVQFVDWCPTGFKCGINYQPP
OsTUA3-1      MMAKCDPRHGKYMACCLMYRGDVVPKDVNAAVATIKTKRTIQFVDWCPTGFKCGINYQPP
OsTUA3-2      MMAKCDPRHGKYMACCLMYRGDVVPKDVNAAVATIKTKRTIQFVDWCPTGFKCGINYQPP
               *.***************:**************:  :***:**:*********** ****:**

SsTUA         TVVPGGDLAKVQRAVCMLSNTTAIAEAWARLDHKFDLMYAKRAFVHWYVGEGMEEGEFSE
DsTubulin     TVVPGGDLAKVQRAVCMISNSTAIGEIFSRLDHKFDLMYAKRAFVHWYVGEGMEEGEFSE
OsTUA2        TAVPGGDLAKVRRAVCMISNNTAVAEVFSRIDRKFDLMYAKRAFVHWYVGEGMEEGEFSE
OsTUA1        SVVPGGDLAKVQRAVCMISNNTAVAEVFSRIDHKFDLMYAKRAFVHWYVGEGMEEGEFSE
OsTUA3-1      SVVPSGDLAKVQRAVCMISNSTSVVEVFSRIDHKFDLMYSKRAFVHWYVGEGMEEGEFSE
OsTUA3-2      SVVPGGDLAKVQRAVCMISNSTSVVEVFSRIDIKFDLMYSKRAFVHWYVGEGMEEGEFSE
              :.**.******:*****:**.*:: * ::*:* ******:*********************

SsTUA         AREDMAALEKDYEEVGVDSVEGEGEEEGEEY
DsTubulin     AREDLAALEKDFEEVGAESADGAGEGEGEEY
OsTUA2        AREDLAALEKDYEEVGAEVEDDDEEQ-GE--
OsTUA1        AREDLAALEKDYEEVGAEGADDEN-DDGEDY
OsTUA3-1      AREDLAALEKDYEEVGAESDENEDGDDGDEY
OsTUA3-2      AREDLAALEKDYEEVGSEFDDGDEGDEGDEY
              ****:******:**** :   :       *:
```

Supplemental Figure S4. α-Tubulin homology analysis among *Dunaliella salina*, Rice and *Sus scrofa*. DsTubulin is α-tubulin in *D. salina* form our results, which was match from *Chloromonas* sp. ANT3. OsTUA1, OsTUA2, OsTUA3-1 and OsTUA3-2 are four rice α-tubulin isoforms. This data obtained through Clustal Omega software (http://www.ebi.ac.uk/Tools/msa/clustalo/), which shows that the α-tubulins are highly conserved among alga, plants and *S. scrofa*. Phosphorylation site T349 of α-tubulin is completely conserved. The DsTubulin 3D structure homology model are subjected to SWISS-MODEL database (https://swissmodel.expasy.org). Phosphorylation site T349 is inside the alpha domain interface region of α-tubulin domain. Blue square indicates the identified phosphopeptide in our result, red square showed the phosphorylation site location, and black square indicated the alpha domain interface region. Pink square show the region of alpha domain interface region. An asterisk indicates completely conserved residues; The colon indicates highly conserved residues, the dot indicates a weak conservative district, and the short line indicates the vacancies residues of optimal sequence alignment.
